# Supplementary material for: Culex quinquefasciatus Resistant to the Binary Toxin from Lysinibacillus sphaericus Displays a Consistent Downregulation of Pantetheinase Transcripts
Source: Biomolecules. 2023 Dec 25;14(1):33. doi: 10.3390/biom14010033 (PMC10813629; doi:10.3390/biom14010033)

Table S1. Primers used to perform the RT-qPCR reactions to evaluate the expression of *Culex quinquefasciatus* genes.

Gene ID[1]	Amplicon (bp)	Primers (5'-3')[2]	Blast verification (first and second hits)				
			Description (*Culex quinquefasciatus* taxid:7176)	Acession	% Query cover	E. value	% ID
18S	159	F CGCGGTAATTCCAGCTCCACTA	NA	NA	NA	NA	NA
		R GCATCAAGCGCCACCATATAGG	NA	NA	NA	NA	NA
CPIJ013173 (*cqm1*)	228	F GAACCGGACTCGAAGGACTG	maltase 2 (LOC6046508)	XM_001863660.2	100	4e-04	100
			protein AF-9 (LOC6034974)	XM_038260469.1	80	0.092	100
		R CTCCGGCTGAATATCGTAGAAATC	maltase 2 (LOC6046508)	XM_001863660.2	100	0.14	91.67
			maltase A1 (LOC6037642)	XM_001847477.2	100	0.14	91.67
CPIJ017593 (*pan*)	228	F TCTTTGCCTGCACCGGGACCA	vanin-like protein 1 (LOC6040365)	XM_001849713.2	100	0.027	95.24
			Y+L amino acid transporter 2 (LOC6051251)	XM_038255127.1	71	0.43	100
		R CGAATGGTTTGCCATCCTTCGT	vanin-like protein 1 (LOC6040365)	XM_001849713.2	100	3e-05	100
			LOW QUALITY PROTEIN: (LOC6035613)	XM_038259122.1	68	6.6	100
CPIJ012066 (*NaCl*)	214	F GAAGTTGTGACCTTTGCTTG	sodium-dependent nutrient amino acid transporter1 (LOC6045025)	XM_001862410.2	100	4e-04	100
			peroxisomal membrane protein 11C (LOC6042422)	XM_001851490.2	80	0.092	100
		R ATCCGAATGCGTACATCC	sodium-dependent nutrient amino acid transporter1 (LOC6045025)	XM_001862410.2	100	0.004	100
			sodium-dependent nutrient amino acid transporter1 (LOC6045026)	XM_038254677.1	100	0.96	94.44
CPIJ010801 (*cbB*)	196	F ATTGATGTCGCAAATCGTGG	carboxypeptidase B (LOC6043137)	XM_001856118.2	100	4e-04	100
			carboxypeptidase B (LOC6043138)	XM_001856123.2	95	0.36	94.74
		R CGGCTTCAATTGCTTCAAC	carboxypeptidase B (LOC6043137)	XM_001856118.2	100	19	100
			carboxypeptidase B (LOC6043138)	XM_001856123.2	100	19	100
CPIJ002522 (*far*)	211	F GAGGCTTATCAGTCGATGAA	farnesol dehydrogenase (LOC6033789)	XM_001844139.2	100	4e-04	100
			multidrug resistance-associated protein 7 (LOC6035986)	XM_038253057.1	80	5.7	100
		R GGCTGATGCTGGTAACCTTC	farnesol dehydrogenase (LOC6033789)	XM_001844139.2	100	4e-04	100
			sorting nexin-8 transcript variant X2 (LOC6037692)	XM_038254173.1	80	0.092	100

[1] *18S*: ribossomal protein control gene [1]. *cqm1*: *Culex quinquefasciatus* maltase 1[2]. *pan* : pantetheinase [2]. *NaCl*: sodium/chloride dependent amino acid transporter, putative. *cBb*: carboxypeptidase B precursor. *far*: farnesol dehydrogenase. NA: Not Applicable

[2] Primers forward (F) and reverse (R) were designed using PrimerSelect (DNASTAR) based on the sequences obtained in the VectorBase platform (www.vectorbase.org).

Reference cited

1. Liu, Y.; Zhang, H.; Qiao, C.; Lu, X.; Cui, F. Correlation between carboxylesterase alleles and insecticide resistance in *Culex pipiens* complex from China. *Parasit Vectors* **2011**, *4*, 236, doi:10.1186/1756-3305-4-236.

2. Rezende, T.M.T., Rezende, A.M., Luz Wallau, G., Santos Vasconcelos, C.R., de-Melo-Neto, O.P., Silva-Filha, M., Romao, T.P. A differential transcriptional profile by *Culex quinquefasciatus* larvae resistant to *Lysinibacillus sphaericus* IAB59 highlights genes and pathways associated with the resistance phenotype. *Parasit Vectors* **2019**, 12, 407, doi:10.1186/s13071-019-3661-y.

Tabela S2. Statistics for the sequenced RNA-seq libraries of *Culex quinquefasciatus* larvae from two Bin-resistant strains (REC, REC-2) compared to a susceptible one (S).

	REC		S		REC-2		S	
Reads total	6,256,898		8,275,658		6,230,726		9,220,190	
	Reads (10^6)	%	Reads (10^6)	%	Reads (10^6)	%	Reads (10^6)	%
Mapping results								
Total mapped	6.13	98.1	8.12	98.9	6.17	99.1	9.12	99
Unique mapped	3.23	51.6	5.56	66.1	3.39	53.1	6.44	69.6
Multiple mapped	1.68	26.7	1.92	24.4	1.13	17.3	1.96	21.5
Unmapped	1.21	13.0	0.69	8.30	1.64	18.7	0.72	7.9

Table S3. Dataset Principal Component Analysis (PCA) of the expression profile generated by RNA-seq comparing the *Culex quinquefasciatus* larvae from two Bin-resistant strains (REC and REC-2) and the susceptible strain (S). REC (replicates A11. A12. A13) was compared to S (replicates S1, S2, S3). REC-2 (replicates A21. A22. A23) was compared to S (replicates S1, S2, S3).

Comparison REC X S	Coordinates of the graph					
	PC1	PC2	PC3	PC4	PC5	PC6
A11.counts	-19,008683	7,52328523	1,92914547	10,324726	-4,4878443	4,61E-14
A12.counts	-20,809536	6,56798523	-11,786851	-3,5778055	3,15522284	4,79E-14
A13.counts	-25,631937	2,70355081	10,4716906	-7,0682654	1,66164114	4,50E-14
S1_S4.counts	19,6586423	-7,0430559	1,6491045	5,30234012	9,03386132	2,30E-14
S2_S5.counts	-5,3914479	-17,438324	-2,5314232	-1,6144919	-5,7370166	4,18E-14
S3_S6.counts	51,1829622	7,6865582	0,26833403	-3,3665034	-3,6258644	-2,56E-14
	PCA statistics					
	PC1	PC2	PC3	PC4	PC5	PC6
Standard deviation	29,9552619	10,1963756	7,23197653	6,50254988	5,66618269	4,79E-14
Proportion of Variance	0,79551	0,09217	0,04637	0,03749	0,02846	0
Cumulative Proportion	0,79551	0,88768	0,93405	0,97154	1	1

Comparison REC-2 X S	Coordinates of the graph					
	PC1	PC2	PC3	PC4	PC5	PC6
A21.counts	-48,512778	9,65931203	8,53835823	8,54725255	-0,4947761	1,05E-13
A22.counts	-1,7371684	-6,9022345	-19,698955	6,63621501	2,22477578	6,12E-14
A23.counts	-37,402016	-1,1397208	-3,9574395	-13,409359	-2,1662717	4,40E-14
S1.counts	22,1818052	-0,4888534	7,45212784	-2,6538512	12,3183715	1,20E-14
S2.counts	18,6677042	-16,97695	10,3822533	2,04959256	-6,4584405	-2,31E-14
S3.counts	46,8024537	15,8484469	-2,7163452	-1,16985	-5,4236589	-6,69E-14
	PCA statistics					
	PC1	PC2	PC3	PC4	PC5	PC6
Standard deviation	36,8412909	11,6779382	11,378204	7,86788521	6,82285822	7,29E-14
Proportion of Variance	0,78384	0,07876	0,07477	0,03575	0,02688	0
Cumulative Proportion	0,78384	0,8626	0,93737	0,97312	1	1

Table S4. Differentially expressed genes from the *Culex quinquefasciatus* Bin-resistant strains (REC and REC-2), compared to a susceptible strain (S).

(A) REC X S up and down regulated genes.

ID	Description	Uniprot Annotation	log2FoldChange	padj	baseMean	lfcSE	stat	pvalue
CPIJ017593	pantetheinase precursor		-8,776	0,000	55,426	1,213	-7,236	4,630E-13
CPIJ018744	ankyrin 2,3/unc44		-8,446	0,000	44,418	1,227	-6,882	5,883E-12
CPIJ012700	CHKov1, putative		-7,933	0,000	31,080	1,223	-6,488	8,674E-11
CPIJ017592	Vanin-like protein 1 precursor, putative		-7,866	0,000	29,438	1,221	-6,445	1,159E-10
CPIJ003695	glucosyl/glucuronosyl transferases		-7,752	0,000	53,136	1,216	-6,376	1,819E-10
CPIJ012697	Putative uncharacterized protein	CHK kinase-like domain-containing	-7,455	0,000	22,391	1,234	-6,041	1,536E-09
CPIJ014435	Putative uncharacterized protein	Cuticle protein	-6,947	0,000	15,229	1,260	-5,515	3,491E-08
CPIJ014889	Pyruvate dehydrogenase E1 component subunit alpha		-6,721	0,000	13,540	1,261	-5,331	9,778E-08
CPIJ005154	Nuclear transport factor 2		-6,720	0,000	13,461	1,246	-5,394	6,879E-08
CPIJ012840	39S ribosomal protein L24, mitochondrial precursor		-6,549	0,000	12,008	1,253	-5,226	1,734E-07
CPIJ013173	Neutral and basic amino acid transport protein rBAT	Culex quinquefasciatus maltase 1	-5,840	0,000	712,368	0,309	-18,910	9,354E-80
CPIJ012702	Putative uncharacterized protein	CHK kinase-like domain-containing	-5,686	0,000	6,328	1,280	-4,442	8,903E-06
CPIJ007230	Regucalcin		-5,522	0,000	5,995	1,287	-4,292	1,768E-05
CPIJ019851	Myosin i		-5,219	0,001	4,886	1,288	-4,053	5,067E-05
CPIJ018693	Hyp37.3 putative secreted salivary gland protein		-5,205	0,000	34,821	0,815	-6,390	1,663E-10
CPIJ016041	Putative uncharacterized protein	CsbD family protein	-5,153	0,001	4,684	1,296	-3,976	7,006E-05
CPIJ006734	DNA-directed RNA polymerase II 16 kDa polypeptide		-4,975	0,002	8,130	1,270	-3,918	8,945E-05
CPIJ015938	Putative uncharacterized protein		-4,956	0,003	4,160	1,306	-3,796	1,472E-04
CPIJ008878	prolylcarboxypeptidase, putative		-4,814	0,003	6,985	1,275	-3,776	1,596E-04
CPIJ005644	protein lethal(2)essential for life		-4,809	NA	90,328	1,811	-2,655	NA
CPIJ013539	ankyrin repeat and SOCS box protein 10		-4,747	0,004	3,455	1,290	-3,681	2,326E-04
CPIJ002103	Putative uncharacterized protein		-4,565	0,000	848,780	0,667	-6,844	7,681E-12
CPIJ002989	Oseg1		-4,431	0,007	5,564	1,274	-3,478	5,053E-04
CPIJ006124	prolylcarboxypeptidase, putative		-4,419	0,000	148,476	0,685	-6,448	1,132E-10
CPIJ008807	ficolin-1 precursor		-4,291	0,012	2,452	1,301	-3,299	9,709E-04
CPIJ015947	purine nucleoside phosphorylase		-4,288	0,012	2,516	1,300	-3,298	9,735E-04
CPIJ012061	hyp37.3 putative secreted salivary gland protein		-4,245	0,000	18,738	0,855	-4,963	6,952E-07
CPIJ017634	Disulfide oxidoreductase		-4,158	0,013	4,633	1,280	-3,249	1,160E-03
CPIJ007736	Steroid dehydrogenase		-4,151	0,000	12,782	0,927	-4,478	7,537E-06
CPIJ019567	vegetatible incompatibility protein HET-E-1, putative		-4,067	0,005	8,166	1,125	-3,614	3,012E-04
CPIJ012699	Putative uncharacterized protein	CHK kinase-like domain-containing	-3,905	0,007	7,368	1,121	-3,484	4,934E-04
CPIJ012066	sodium/chloride dependent amino acid transporter, putative		-3,853	0,000	438,976	0,694	-5,554	2,799E-08
CPIJ012180	Putative uncharacterized protein	MD-2-related lipid-recognition dom	-3,837	0,000	920,730	0,311	-12,332	6,083E-35
CPIJ007385	serine-type enodpeptidase, putative		-3,764	0,000	56,310	0,500	-7,520	5,458E-14
CPIJ003858	Pigeon protein		-3,757	0,031	3,572	1,287	-2,919	3,509E-03

CPIJ018694	Ion channel nompc		-3,695	0,001	36,389	0,913	-4,046	5,210E-05
CPIJ015475	Thiamin pyrophosphokinase 1		-3,664	0,038	3,521	1,295	-2,829	4,664E-03
CPIJ009793	Glioma tumor suppressor candidate region gene 2 protein		-3,562	0,001	11,317	0,861	-4,139	3,494E-05
CPIJ008664	Putative uncharacterized protein	Protein TsetseEP domain-containing	-3,478	0,000	92,332	0,797	-4,365	1,270E-05
CPIJ012888	rRNA processing protein Ebp2		-3,446	0,030	5,095	1,173	-2,936	3,321E-03
CPIJ019447	bestrophin 2,3,4		-3,406	0,058	2,968	1,299	-2,623	8,717E-03
CPIJ000540	Putative uncharacterized protein	Neuroguidin	-3,382	0,006	7,640	0,955	-3,541	3,989E-04
CPIJ003753	Methionyl-tRNA formyltransferase		-3,380	0,060	2,735	1,299	-2,602	9,258E-03
CPIJ011728	Serine/threonine protein kinase		-3,370	0,028	4,955	1,141	-2,952	3,157E-03
CPIJ007384	azurocidin precursor		-3,364	0,001	719,258	0,840	-4,003	6,250E-05
CPIJ014772	techylectin-5B precursor		-3,344	0,005	10,153	0,935	-3,578	3,463E-04
CPIJ018626	Glutathione-s-transferase theta, gst		-3,323	0,000	199,246	0,380	-8,757	2,012E-18
CPIJ008485	calponin/transgelin		-3,317	0,032	4,822	1,143	-2,903	3,694E-03
CPIJ016341	alpha-esterase		-3,203	0,000	15,887	0,679	-4,717	2,392E-06
CPIJ008877	prolylcarboxypeptidase, putative		-3,189	0,000	48,072	0,432	-7,390	1,465E-13
CPIJ004872	protein phosphatase 2C isoform beta		-3,086	0,000	14,704	0,717	-4,301	1,701E-05
CPIJ018326	Putative uncharacterized protein	MD-2-related lipid-recognition dom	-3,034	0,018	235,573	0,964	-3,146	1,654E-03
CPIJ007525	Putative uncharacterized protein	Gamma-interferon inducible lysosor	-3,021	0,000	21,224	0,596	-5,072	3,946E-07
CPIJ008659	metalloproteinase, putative		-2,974	0,161	11,848	1,435	-2,072	3,825E-02
CPIJ015241	Alkaline phosphatase		-2,869	0,007	9,129	0,821	-3,494	4,752E-04
CPIJ016853	CYP6N21P: cytochrome P450 6N21P		-2,847	0,000	64,517	0,474	-6,003	1,937E-09
CPIJ007940	Putative uncharacterized protein	E3 ubiquitin-protein ligase trim33	-2,831	0,079	3,671	1,148	-2,467	1,363E-02
CPIJ018624	Glutathione-s-transferase theta, gst		-2,825	0,000	71,136	0,396	-7,129	1,012E-12
CPIJ009980	Eukaryotic translation initiation factor 4 gamma		-2,814	0,056	19,218	1,067	-2,639	8,327E-03
CPIJ004709	Bifunctional protein folD		-2,783	0,000	44,070	0,488	-5,701	1,194E-08
CPIJ003579	Putative uncharacterized protein	DUF4536 domain-containing protei	-2,767	0,037	5,288	0,977	-2,833	4,615E-03
CPIJ010271	Putative uncharacterized protein	C2H2-type domain-containing prote	-2,760	0,012	8,373	0,837	-3,297	9,782E-04
CPIJ014142	nucleotide-binding protein 1		-2,749	0,000	17,291	0,619	-4,438	9,089E-06
CPIJ003007	elongase, putative		-2,739	0,000	15,860	0,624	-4,391	1,127E-05
CPIJ000909	Putative uncharacterized protein	F-box domain-containing protein	-2,736	0,008	9,026	0,801	-3,415	6,388E-04
CPIJ014557	Nitrilase and fragile histidine triad fusion protein NitFhit		-2,721	0,002	12,182	0,695	-3,914	9,076E-05
CPIJ016849	CYP6M12: cytochrome P450 6M12		-2,689	0,000	71,441	0,413	-6,514	7,318E-11
CPIJ011602	NADH-cytochrome b5 reductase		-2,676	0,011	8,197	0,802	-3,337	8,472E-04
CPIJ006909	Putative uncharacterized protein	FHA domain-containing protein	-2,671	0,111	3,408	1,168	-2,286	2,224E-02
CPIJ013880	Heat shock protein 67B2		-2,645	0,003	89,506	0,705	-3,754	1,744E-04
CPIJ019704	CYP6N24: cytochrome P450 6N24		-2,634	0,050	4,947	0,978	-2,693	7,091E-03
CPIJ007346	TTC27 protein		-2,630	0,051	4,701	0,980	-2,682	7,310E-03
CPIJ016731	Putative uncharacterized protein	Macro domain-containing protein	-2,590	0,120	3,180	1,154	-2,244	2,480E-02

CPIJ012829	Fibrinogen and fibronectin		-2,572	0,000	97,643	0,323	-7,961	1,710E-15
CPIJ004285	neural/ectodermal development factor IMP-L2 precursor		-2,513	0,066	4,903	0,982	-2,558	1,053E-02
CPIJ007831	Putative uncharacterized protein	RING-type domain-containing prote	-2,511	0,138	3,066	1,158	-2,169	3,012E-02
CPIJ008422	Serine esterase		-2,500	0,000	74,158	0,365	-6,842	7,823E-12
CPIJ017699	arginyl-tRNA-protein transferase 1		-2,482	0,143	3,072	1,154	-2,151	3,151E-02
CPIJ015882	Putative uncharacterized protein	RRM domain-containing protein	-2,477	0,000	36,107	0,504	-4,916	8,817E-07
CPIJ005656	Oxidoreductase		-2,472	0,000	18,195	0,565	-4,376	1,207E-05
CPIJ005214	Ceramide glucosyltransferase		-2,452	0,000	18,824	0,553	-4,433	9,315E-06
CPIJ003456	Uricase		-2,447	0,002	14,917	0,643	-3,808	1,401E-04
CPIJ001035	Putative uncharacterized protein	Lipase domain-containing protein	-2,444	0,000	252,134	0,358	-6,823	8,893E-12
CPIJ015623	Predicted protein		-2,423	0,014	8,491	0,750	-3,230	1,236E-03
CPIJ007080	trypsin-1 precursor		-2,391	0,005	14,538	0,661	-3,618	2,969E-04
CPIJ002336	Putative uncharacterized protein	Acetyl-coa transporter	-2,372	0,001	19,241	0,588	-4,036	5,427E-05
CPIJ012841	Valacyclovir hydrolase		-2,372	0,170	2,830	1,166	-2,035	4,190E-02
CPIJ009939	Putative uncharacterized protein		-2,360	0,016	8,226	0,743	-3,177	1,489E-03
CPIJ001394	Williams Beuren syndrome chromosome region 22		-2,359	0,018	8,148	0,750	-3,145	1,662E-03
CPIJ002679	Glutathione S-transferase theta-2		-2,329	0,338	115,278	1,526	-1,526	1,270E-01
CPIJ018565	Peptide methionine sulfoxide reductase		-2,316	0,006	12,254	0,660	-3,512	4,451E-04
CPIJ005818	13 kDa ribonucleoprotein-associated protein		-2,314	0,000	26,139	0,538	-4,302	1,693E-05
CPIJ008588	mRNA turnover protein 4		-2,306	0,000	20,313	0,537	-4,295	1,747E-05
CPIJ001371	serine/threonine-protein phosphatase 4 catalytic subunit		-2,304	0,026	7,803	0,768	-3,000	2,698E-03
CPIJ010601	N-terminal acetyltransferase A complex catalytic subunit ARD1		-2,302	0,042	6,657	0,828	-2,782	5,402E-03
CPIJ013923	glucosyl/glucuronosyl transferases		-2,293	0,036	6,820	0,804	-2,853	4,329E-03
CPIJ006965	Putative uncharacterized protein	Protein takeout	-2,292	0,060	5,279	0,879	-2,606	9,170E-03
CPIJ003123	DNA-directed RNA polymerase I 40 kDa polypeptide		-2,284	0,037	6,786	0,806	-2,833	4,610E-03
CPIJ000580	Ladybird homeobox corepressor		-2,280	0,193	2,821	1,171	-1,948	5,147E-02
CPIJ005158	Putative uncharacterized protein	Vesicle transport protein SEC20	-2,268	0,108	3,974	0,985	-2,302	2,135E-02
CPIJ017623	Tetratricopeptide repeat protein 1		-2,265	0,108	3,893	0,984	-2,301	2,137E-02
CPIJ019838	Mitochondrial oxodicarboxylate carrier		-2,264	0,106	4,016	0,980	-2,309	2,095E-02
CPIJ003710	Putative uncharacterized protein	WKF domain-containing protein	-2,247	0,042	6,545	0,808	-2,780	5,429E-03
CPIJ002590	Amsh		-2,236	0,027	7,630	0,753	-2,969	2,983E-03
CPIJ006542	Chymotrypsin-2		-2,215	0,004	13,787	0,609	-3,636	2,772E-04
CPIJ016639	acetyl-coa synthetase		-2,186	0,000	47,386	0,413	-5,293	1,204E-07
CPIJ011433	Brachyurin		-2,186	0,056	76,934	0,829	-2,637	8,364E-03
CPIJ008889	60 kDa heat shock protein, mitochondrial precursor		-2,174	0,103	31,600	0,934	-2,328	1,993E-02
CPIJ003580	cle7, putative		-2,157	0,050	6,287	0,801	-2,691	7,125E-03
CPIJ016855	CYP6N20: cytochrome P450 6N20		-2,154	0,000	135,442	0,280	-7,683	1,558E-14
CPIJ013919	Xanthine dehydrogenase/oxidase		-2,152	0,000	692,409	0,268	-8,038	9,154E-16
CPIJ016847	CYP6CQ1: cytochrome P450 6CQ1		-2,142	0,230	2,592	1,177	-1,819	6,890E-02

CPIJ001505	Putative uncharacterized protein	Malectin domain-containing protein	-2,142	0,007	13,151	0,620	-3,453	5,549E-04
CPIJ006912	Maggie		-2,126	0,036	8,121	0,745	-2,852	4,340E-03
CPIJ013082	Chitinase		-2,124	0,023	9,527	0,699	-3,038	2,382E-03
CPIJ012177	iodotyrosine dehalogenase 1 precursor		-2,102	0,098	4,913	0,893	-2,354	1,859E-02
CPIJ017390	cytosolic Fe-S cluster assembling factor CFD1		-2,099	0,036	8,167	0,735	-2,856	4,287E-03
CPIJ000536	Secreted protein		-2,088	0,152	3,477	0,990	-2,108	3,499E-02
CPIJ001316	6-phosphogluconate dehydrogenase, decarboxylating		-2,082	0,000	33,091	0,422	-4,929	8,282E-07
CPIJ009452	Monocarboxylate transporter		-2,078	0,099	4,692	0,884	-2,351	1,870E-02
CPIJ009924	Putative uncharacterized protein		-2,059	0,039	8,288	0,730	-2,822	4,773E-03
CPIJ001337	Uridine 5-monophosphate synthase		-2,055	0,106	4,898	0,891	-2,307	2,103E-02
CPIJ009935	mannosyl-oligosaccharide alpha-1,2-mannosidase		-2,041	0,039	8,018	0,724	-2,818	4,832E-03
CPIJ012503	gamma-glutamylcysteine synthetase, putative		-2,018	0,042	8,092	0,725	-2,784	5,372E-03
CPIJ013917	esterase B1 precursor		-2,014	0,000	256,649	0,279	-7,213	5,481E-13
CPIJ019868	Heat shock 70 kDa protein 4		-2,007	0,215	17,558	1,072	-1,872	6,124E-02
CPIJ001974	Putative uncharacterized protein	Mitochondrial cytochrome c oxidase	-2,001	0,064	6,746	0,778	-2,572	1,010E-02
CPIJ008943	Sugar transporter		-1,995	0,143	4,618	0,928	-2,149	3,161E-02
CPIJ018627	Glutathione S-transferase 1-1		-1,994	0,000	181,067	0,262	-7,603	2,899E-14
CPIJ011962	Multidrug resistance-associated protein 14		-1,989	0,012	13,336	0,604	-3,292	9,945E-04
CPIJ006688	kinesin heavy chain subunit		-1,978	0,004	18,947	0,542	-3,649	2,633E-04
CPIJ002675	Glutathione S-transferase 1		-1,956	0,000	1094,519	0,338	-5,783	7,351E-09
CPIJ010538	CYP9J46: cytochrome P450 9J46		-1,936	0,000	62,783	0,413	-4,686	2,783E-06
CPIJ019225	Calmodulin		-1,936	0,049	8,407	0,713	-2,714	6,647E-03
CPIJ009657	Putative uncharacterized protein	Nucleolar protein 16	-1,932	0,077	6,667	0,779	-2,479	1,318E-02
CPIJ004369	glucosyl transferase		-1,930	0,000	35,373	0,422	-4,570	4,874E-06
CPIJ007774	Putative uncharacterized protein	VPS9 domain-containing protein	-1,922	0,079	6,907	0,778	-2,469	1,355E-02
CPIJ016672	Putative uncharacterized protein	Mitochondrial import inner membra	-1,907	0,114	5,350	0,839	-2,273	2,305E-02
CPIJ005033	Nucleolar protein NHP2		-1,905	0,005	16,343	0,531	-3,591	3,295E-04
CPIJ007770	DNA-directed RNA polymerase III subunit D		-1,904	0,202	3,390	0,996	-1,912	5,587E-02
CPIJ017540	Coatomer subunit delta		-1,895	0,012	23,050	0,579	-3,275	1,058E-03
CPIJ011644	Ubiquitin-protein ligase		-1,893	0,063	7,385	0,735	-2,576	9,997E-03
CPIJ020069	Methionine aminopeptidase 2		-1,888	0,060	7,412	0,725	-2,604	9,213E-03
CPIJ011687	Inosine-5-monophosphate dehydrogenase		-1,887	0,013	16,714	0,578	-3,265	1,096E-03
CPIJ015011	Putative uncharacterized protein		-1,880	0,031	11,688	0,644	-2,918	3,521E-03
CPIJ011381	serine-type enodpeptidase, putative		-1,873	0,000	187,196	0,335	-5,592	2,245E-08
CPIJ015373	S-formylglutathione hydrolase		-1,871	0,001	23,933	0,468	-3,995	6,467E-05
CPIJ011770	Uncharacterized protein (Fragment)		-1,869	0,062	8,756	0,721	-2,591	9,578E-03
CPIJ015645	Putative uncharacterized protein	non-specific serine/threonine protei	-1,851	0,068	7,103	0,728	-2,543	1,098E-02
CPIJ015106	tonin precursor		-1,845	0,002	29,419	0,476	-3,877	1,057E-04
CPIJ008116	Adenosylhomocysteinase		-1,842	0,368	30,815	1,268	-1,453	1,462E-01

CPIJ010515	Phosphoenolpyruvate carboxykinase		-1,832	0,237	3,319	1,018	-1,801	7,174E-02
CPIJ016097	Peroxisomal membrane protein 70 abcd3		-1,824	0,003	38,812	0,482	-3,782	1,556E-04
CPIJ005595	Fatty acid synthase S-acetyltransferase		-1,815	0,000	136,135	0,371	-4,891	1,003E-06
CPIJ013918	esterase B1 precursor		-1,811	0,000	760,449	0,207	-8,749	2,159E-18
CPIJ014453	Putative uncharacterized protein	Arrestin C-terminal-like domain-cor	-1,780	0,086	6,995	0,733	-2,427	1,524E-02
CPIJ001564	Selenide		-1,775	0,000	47,760	0,388	-4,578	4,699E-06
CPIJ016846	CYP6M13: cytochrome P450 6M13		-1,772	0,421	85,859	1,329	-1,333	1,825E-01
CPIJ016253	transport and Golgi organization, putative		-1,771	0,125	5,878	0,797	-2,221	2,634E-02
CPIJ011032	Putative uncharacterized protein	Small-subunit processome Utp12 do	-1,756	0,079	8,708	0,712	-2,465	1,370E-02
CPIJ005830	glutaredoxin, putative		-1,742	0,016	21,385	0,547	-3,185	1,449E-03
CPIJ011600	Long-chain-fatty-acid coa ligase		-1,742	0,002	64,809	0,448	-3,888	1,010E-04
CPIJ011457	Putative uncharacterized protein	DUF4149 domain-containing protei	-1,741	0,001	34,876	0,420	-4,144	3,419E-05
CPIJ015682	Translation initiation factor if-2		-1,739	0,017	15,505	0,549	-3,167	1,539E-03
CPIJ016852	CYP6N19: cytochrome P450 6N19		-1,739	0,000	179,270	0,381	-4,567	4,953E-06
CPIJ004017	Cyclin l		-1,737	0,080	8,374	0,706	-2,459	1,394E-02
CPIJ019233	Cerebral protein 1		-1,735	0,084	8,323	0,712	-2,438	1,477E-02
CPIJ015759	chondroitin sulfate synthase 1		-1,734	0,083	7,814	0,709	-2,444	1,454E-02
CPIJ004081	protein Peter pan		-1,733	0,096	6,690	0,730	-2,373	1,766E-02
CPIJ010146	Putative uncharacterized protein	VWFA domain-containing protein	-1,733	0,054	11,216	0,652	-2,658	7,852E-03
CPIJ017319	Putative uncharacterized protein		-1,730	0,049	10,695	0,640	-2,703	6,881E-03
CPIJ007014	Splicing factor 3B subunit 3		-1,722	0,015	33,310	0,537	-3,208	1,337E-03
CPIJ001891	Putative uncharacterized protein	CCHC-type domain-containing prot	-1,717	0,000	223,281	0,261	-6,575	4,850E-11
CPIJ019673	CYP6AG15: cytochrome P450 6AG15		-1,714	0,000	38,700	0,389	-4,408	1,044E-05
CPIJ016931	Glutamate-rich WD repeat-containing protein 1		-1,714	0,000	35,901	0,399	-4,295	1,747E-05
CPIJ002591	Mitochondrial solute carrier		-1,713	0,025	13,420	0,569	-3,013	2,587E-03
CPIJ015367	Putative uncharacterized protein		-1,704	0,270	3,075	1,001	-1,703	8,852E-02
CPIJ011261	Cell cycle control protein cwf8		-1,700	0,097	6,799	0,719	-2,363	1,813E-02
CPIJ012065	tryptophan transporter		-1,698	0,002	33,167	0,445	-3,818	1,344E-04
CPIJ009821	RpI12		-1,698	0,102	6,940	0,729	-2,331	1,977E-02
CPIJ006732	Gamma-tubulin complex component		-1,688	0,219	4,023	0,909	-1,857	6,333E-02
CPIJ015262	Heterogeneous nuclear ribonucleoprotein 27C		-1,688	0,011	25,556	0,507	-3,330	8,672E-04
CPIJ002063	Mitochondrial ribosomal protein L42		-1,685	0,016	15,936	0,531	-3,173	1,510E-03
CPIJ005510	Putative uncharacterized protein	rRNA-processing protein UTP23	-1,674	0,222	3,858	0,906	-1,847	6,474E-02
CPIJ009240	Ganglioside-induced differentiation-associated protein 1		-1,672	0,005	21,254	0,465	-3,593	3,268E-04
CPIJ012125	FK506 binding protein		-1,672	0,280	2,969	1,000	-1,672	9,445E-02
CPIJ005348	lipase 3 precursor		-1,670	0,050	10,589	0,618	-2,702	6,903E-03
CPIJ001915	WD repeat domain 50		-1,662	0,078	8,429	0,672	-2,473	1,339E-02
CPIJ005505	Putative uncharacterized protein		-1,660	0,005	26,419	0,461	-3,602	3,153E-04
CPIJ008595	ran		-1,659	0,022	22,095	0,542	-3,063	2,189E-03

CPIJ010842	Transmembrane trafficking protein		-1,657	0,044	15,281	0,602	-2,754	5,879E-03
CPIJ008647	Atlastin		-1,652	0,027	15,483	0,555	-2,974	2,937E-03
CPIJ000418	Putative uncharacterized protein	J domain-containing protein	-1,651	0,151	5,811	0,781	-2,114	3,451E-02
CPIJ000710	Putative uncharacterized protein	C3H1-type domain-containing prote	-1,649	0,090	8,340	0,685	-2,406	1,612E-02
CPIJ001862	Sodium/solute symporter		-1,648	0,125	6,825	0,742	-2,221	2,634E-02
CPIJ006182	Zinc finger protein		-1,648	0,229	3,970	0,903	-1,824	6,809E-02
CPIJ016145	Predicted protein		-1,646	0,094	8,722	0,691	-2,382	1,722E-02
CPIJ009725	Putative uncharacterized protein	Pre-rRNA-processing protein RIX1	-1,638	0,124	6,713	0,735	-2,227	2,594E-02
CPIJ008477	Acyl-CoA-binding domain-containing protein 6		-1,635	0,090	10,686	0,680	-2,406	1,612E-02
CPIJ000795	H/ACA ribonucleoprotein complex subunit 4		-1,634	0,000	82,017	0,325	-5,036	4,755E-07
CPIJ000770	trypsin precursor		-1,632	0,050	1822,467	0,606	-2,693	7,072E-03
CPIJ012747	uridine cytidine kinase i		-1,632	0,077	9,031	0,658	-2,480	1,314E-02
CPIJ014193	Putative uncharacterized protein	Chitin-binding type-2 domain-conta	-1,631	0,000	173,914	0,323	-5,050	4,416E-07
CPIJ014398	Host cell factor		-1,624	0,162	5,433	0,785	-2,069	3,850E-02
CPIJ005438	exocyst complex component 6		-1,623	0,017	20,057	0,513	-3,163	1,561E-03
CPIJ005168	dolichyl-diphosphooligosaccharide protein glycotransferase		-1,618	0,023	29,980	0,530	-3,051	2,277E-03
CPIJ001361	proteasome subunit beta type 3		-1,615	0,012	20,635	0,490	-3,295	9,837E-04
CPIJ019364	apolipoprotein D, putative		-1,610	0,058	11,515	0,613	-2,626	8,637E-03
CPIJ010392	PET191 polypeptide, putative		-1,610	0,071	9,908	0,639	-2,521	1,171E-02
CPIJ000668	Putative uncharacterized protein	RUN domain-containing protein	-1,610	0,245	3,735	0,904	-1,780	7,510E-02
CPIJ007556	Regulator of ribosome biosynthesis		-1,606	0,012	20,247	0,487	-3,300	9,661E-04
CPIJ010629	Membrane associated progesterone receptor		-1,603	0,002	27,270	0,417	-3,846	1,200E-04
CPIJ009620	Serine/threonine-protein phosphatase 5		-1,603	0,007	24,309	0,463	-3,460	5,393E-04
CPIJ017671	Ubiquitin thioesterase OTU1		-1,603	0,019	17,286	0,514	-3,119	1,814E-03
CPIJ020078	Putative uncharacterized protein	VWFC domain-containing protein	-1,599	0,183	5,625	0,806	-1,984	4,720E-02
CPIJ005674	Cold shock domain-containing protein E1		-1,599	0,114	7,084	0,703	-2,274	2,297E-02
CPIJ009285	Ribosome production factor 1		-1,598	0,042	12,587	0,575	-2,780	5,440E-03
CPIJ001085	Low molecular weight protein-tyrosine-phosphatase		-1,593	0,315	2,878	1,007	-1,581	1,140E-01
CPIJ001476	Shwachman-Bodian-Diamond syndrome protein		-1,579	0,109	7,946	0,687	-2,297	2,160E-02
CPIJ008729	4-aminobutyrate aminotransferase, mitochondrial precursor		-1,572	0,027	14,456	0,530	-2,968	2,997E-03
CPIJ018255	Mitochondrial 28S ribosomal protein S33		-1,569	0,096	8,726	0,662	-2,368	1,788E-02
CPIJ010003	T-complex protein 1 subunit gamma		-1,567	0,098	7,996	0,665	-2,358	1,838E-02
CPIJ013588	Mitochondrial 28S ribosomal protein S2		-1,564	0,049	12,496	0,578	-2,707	6,795E-03
CPIJ005488	Putative uncharacterized protein		-1,562	0,138	6,881	0,720	-2,168	3,017E-02
CPIJ019817	Cytochrome c oxidase assembly protein COX19		-1,561	0,043	13,131	0,564	-2,767	5,656E-03
CPIJ013899	40S ribosomal protein SA		-1,558	0,215	123,026	0,833	-1,869	6,158E-02
CPIJ010586	Transcription elongation factor SPT4		-1,553	0,175	5,322	0,771	-2,014	4,398E-02
CPIJ007179	Prosalpha4: 26S proteasome alpha 4 subunit		-1,552	0,004	32,521	0,420	-3,697	2,184E-04

CPIJ010029	Suppressor of profilin 2		-1,550	0,002	59,859	0,399	-3,884	1,028E-04
CPIJ015054	Putative uncharacterized protein	Anaphase-promoting complex subur	-1,550	0,133	6,951	0,707	-2,191	2,842E-02
CPIJ003661	alpha-1,2-Mannosidase		-1,544	0,047	12,401	0,566	-2,728	6,377E-03
CPIJ020199	CYP6N20: cytochrome P450 6N20		-1,544	0,002	34,559	0,401	-3,847	1,197E-04
CPIJ006617	Putative uncharacterized protein	ascorbate ferrireductase (transmemb	-1,542	0,001	146,903	0,362	-4,256	2,086E-05
CPIJ009724	presenilin sel-12		-1,538	0,103	7,917	0,660	-2,329	1,984E-02
CPIJ015556	RpS28: 40S ribosomal protein S28		-1,532	0,267	47,552	0,894	-1,713	8,663E-02
CPIJ005845	Uncharacterized protein (Fragment)		-1,529	0,350	2,685	1,022	-1,495	1,348E-01
CPIJ004400	ornithine aminotransferase, mitochondrial precursor		-1,525	0,008	59,251	0,443	-3,447	5,672E-04
CPIJ014454	L-lactate dehydrogenase		-1,524	0,080	9,969	0,619	-2,460	1,388E-02
CPIJ000670	24-dehydrocholesterol reductase precursor		-1,522	0,000	514,623	0,237	-6,423	1,339E-10
CPIJ007035	SalLip1: salivary lipase		-1,519	0,541	14,379	1,426	-1,066	2,866E-01
CPIJ016453	Acyl-coa dehydrogenase		-1,519	0,014	24,767	0,471	-3,224	1,262E-03
CPIJ003159	Ribosomal protein P0		-1,512	0,058	19,493	0,577	-2,620	8,782E-03
CPIJ006724	tubulin alpha-1 chain		-1,511	0,000	418,276	0,238	-6,351	2,139E-10
CPIJ007327	protein disulfide-isomerase A6 precursor		-1,510	0,000	202,881	0,349	-4,325	1,524E-05
CPIJ001314	WD repeat protein 46		-1,508	0,062	13,189	0,583	-2,585	9,729E-03
CPIJ019912	Tumor suppressor candidate 4		-1,505	0,173	5,871	0,745	-2,022	4,322E-02
CPIJ007196	Putative uncharacterized protein	Hypoxia up-regulated protein 1	-1,505	0,001	129,796	0,362	-4,155	3,251E-05
CPIJ007404	Guanine nucleotide-binding protein subunit beta 1		-1,505	0,027	16,243	0,507	-2,969	2,991E-03
CPIJ002678	Glutathione transferase I		-1,505	0,022	26,763	0,492	-3,059	2,224E-03
CPIJ016781	Putative uncharacterized protein	H/ACA ribonucleoprotein complex	-1,496	0,193	6,413	0,767	-1,951	5,109E-02
CPIJ005170	GTP-binding protein		-1,491	0,072	11,350	0,593	-2,514	1,192E-02
CPIJ007177	nucleoporin, Nup153, putative		-1,488	0,109	10,008	0,648	-2,297	2,165E-02
CPIJ012068	Sucrose transport protein		-1,488	0,000	166,346	0,310	-4,797	1,609E-06
CPIJ018377	T-complex protein 1 subunit epsilon		-1,487	0,000	97,520	0,316	-4,706	2,522E-06
CPIJ009280	Pgk: Phosphoglycerate kinase		-1,486	0,001	60,445	0,369	-4,030	5,574E-05
CPIJ003558	Deoxyhypusine hydroxylase		-1,486	0,096	11,848	0,628	-2,367	1,793E-02
CPIJ010611	Dual specificity mitogen-activated protein kinase kinase 4 MAPKK4		-1,483	0,135	8,247	0,680	-2,182	2,908E-02
CPIJ012519	E3 ubiquitin-protein ligase RNF123		-1,479	0,221	5,424	0,799	-1,852	6,399E-02
CPIJ019318	Putative uncharacterized protein	N-acetyltransferase domain-containi	-1,478	0,163	6,639	0,716	-2,065	3,891E-02
CPIJ006359	Putative uncharacterized protein		-1,477	0,010	22,438	0,441	-3,350	8,068E-04
CPIJ006932	Ebna2 binding protein P100		-1,477	0,005	100,439	0,411	-3,594	3,252E-04
CPIJ009142	coagulation factor VII precursor		-1,471	0,250	4,412	0,835	-1,761	7,818E-02
CPIJ003399	Peroxiredoxins, prx-1, prx-2, prx-3		-1,467	0,004	41,522	0,397	-3,693	2,217E-04
CPIJ015771	Kinesin light chain 1 and		-1,467	0,063	14,768	0,568	-2,581	9,848E-03
CPIJ017118	Putative uncharacterized protein	CHK kinase-like domain-containing	-1,463	0,072	15,286	0,581	-2,519	1,176E-02
CPIJ004362	Lethal 87Df		-1,462	0,160	7,296	0,703	-2,080	3,750E-02

CPIJ005675	NBP2b protein, putative		-1,462	0,254	4,419	0,836	-1,749	8,031E-02
CPIJ000199	Putative uncharacterized protein		-1,461	0,259	4,218	0,843	-1,733	8,316E-02
CPIJ001395	Translocase of outer membrane 34		-1,456	0,105	11,564	0,629	-2,314	2,068E-02
CPIJ012815	Mannose-1-phosphate guanyltransferase		-1,451	0,039	23,534	0,516	-2,813	4,910E-03
CPIJ013872	Putative uncharacterized protein		-1,450	0,206	5,796	0,763	-1,899	5,751E-02
CPIJ014050	Predicted protein		-1,449	0,002	36,359	0,377	-3,838	1,242E-04
CPIJ011444	Putative uncharacterized protein	tRNA (adenine(58)-N(1))-methyltra	-1,445	0,162	6,887	0,698	-2,069	3,853E-02
CPIJ010410	tRNA-dihydrouridine synthase		-1,443	0,161	7,297	0,697	-2,071	3,836E-02
CPIJ009388	Thermosome subunit alpha		-1,443	0,000	53,636	0,335	-4,312	1,616E-05
CPIJ000616	clip-domain serine protease, putative		-1,442	0,191	6,061	0,737	-1,957	5,037E-02
CPIJ001688	39S ribosomal protein L43, mitochondrial precursor		-1,441	0,161	8,169	0,695	-2,073	3,821E-02
CPIJ016037	Pre-mRNA-splicing factor ISY1		-1,439	0,267	4,190	0,840	-1,713	8,663E-02
CPIJ001706	small nuclear ribonucleoprotein sm d2		-1,439	0,024	18,548	0,476	-3,020	2,526E-03
CPIJ015389	Proliferation-associated 2g4		-1,437	0,001	60,291	0,357	-4,025	5,703E-05
CPIJ008642	disp: protein dispatched (segment polarity protein)		-1,433	0,259	4,198	0,827	-1,733	8,301E-02
CPIJ006520	Putative uncharacterized protein	TM2 domain-containing protein	-1,424	0,246	5,022	0,802	-1,775	7,589E-02
CPIJ011645	Hyperplastic discs protein		-1,422	0,062	19,516	0,550	-2,587	9,675E-03
CPIJ002726	lipase 3 precursor		-1,420	0,117	10,058	0,628	-2,261	2,377E-02
CPIJ003799	protein suppressor of forked		-1,419	0,089	10,930	0,589	-2,410	1,596E-02
CPIJ008450	Peroxiredoxin-6		-1,418	0,003	58,600	0,376	-3,769	1,641E-04
CPIJ007895	mitochondrial dimethyladenosine transferase 1		-1,416	0,111	14,666	0,620	-2,286	2,227E-02
CPIJ007223	Metaxin		-1,415	0,189	7,285	0,720	-1,965	4,936E-02
CPIJ017386	proteasome subunit beta type 8 precursor		-1,413	0,035	26,086	0,494	-2,860	4,242E-03
CPIJ007228	heat shock protein, putative		-1,406	0,001	75,504	0,334	-4,212	2,536E-05
CPIJ019503	DNA cross-link repair 1A protein		-1,402	0,231	5,006	0,771	-1,818	6,909E-02
CPIJ002858	Putative uncharacterized protein	GRAM domain-containing protein	-1,402	0,000	135,472	0,306	-4,587	4,489E-06
CPIJ008204	Microtubule associated protein xmap215		-1,399	0,257	4,735	0,805	-1,738	8,218E-02
CPIJ006139	mitochondrial ribosomal protein, L53, putative		-1,399	0,128	9,199	0,633	-2,211	2,704E-02
CPIJ005330	60S ribosome subunit biogenesis protein NIP7 homolog		-1,399	0,054	14,026	0,526	-2,661	7,797E-03
CPIJ014522	lava lamp protein		-1,397	0,000	76,406	0,324	-4,312	1,619E-05
CPIJ006361	Equilibrative nucleoside transporter		-1,396	0,037	23,921	0,491	-2,843	4,471E-03
CPIJ016450	Leucine carboxyl methyltransferase		-1,393	0,287	4,002	0,843	-1,653	9,832E-02
CPIJ002176	Phosphatidylserine synthase		-1,393	0,038	22,402	0,493	-2,828	4,679E-03
CPIJ010145	Midasin		-1,390	0,028	26,669	0,470	-2,955	3,126E-03
CPIJ010674	Proliferating-cell nucleolar antigen p120		-1,388	0,007	32,866	0,402	-3,454	5,526E-04
CPIJ005010	Fibrillarin		-1,386	0,001	123,008	0,340	-4,074	4,614E-05
CPIJ008264	proteasome subunit beta type 7 precursor		-1,384	0,018	29,445	0,440	-3,143	1,675E-03
CPIJ005257	transmembrane protein 85		-1,375	0,017	22,778	0,436	-3,156	1,599E-03
CPIJ008623	Flotillin-1		-1,375	0,042	20,101	0,495	-2,775	5,515E-03

CPIJ017039	cd36 antigen, putative		-1,374	0,002	51,500	0,356	-3,859	1,138E-04
CPIJ002676	Glutathione S-transferase D7		-1,374	0,000	60,553	0,322	-4,268	1,970E-05
CPIJ001404	Voltage-gated ion channel		-1,370	0,241	5,745	0,766	-1,790	7,352E-02
CPIJ004062	Putative uncharacterized protein	Protein UXT	-1,370	0,294	3,994	0,837	-1,637	1,016E-01
CPIJ005468	Adenosine diphosphatase		-1,368	0,003	36,763	0,369	-3,710	2,075E-04
CPIJ004373	UDP-glucuronosyltransferase		-1,365	0,029	23,495	0,463	-2,947	3,206E-03
CPIJ018234	translocon-associated protein subunit alpha precursor		-1,364	0,002	106,294	0,354	-3,849	1,185E-04
CPIJ015408	branched-chain amino acid aminotransferase		-1,360	0,001	90,419	0,327	-4,158	3,217E-05
CPIJ011806	Superoxide dismutase, Mn		-1,360	0,210	6,253	0,721	-1,888	5,909E-02
CPIJ001666	protein catecholamines up		-1,358	0,063	13,796	0,527	-2,578	9,932E-03
CPIJ017966	Putative uncharacterized protein	Stambha A	-1,356	0,005	38,058	0,380	-3,569	3,589E-04
CPIJ005588	Small nuclear ribonucleoprotein SM D3		-1,351	0,085	12,053	0,556	-2,431	1,505E-02
CPIJ011526	Elongation factor 2		-1,348	0,160	9,276	0,648	-2,079	3,760E-02
CPIJ016848	CYP6M14: cytochrome P450 6M14		-1,347	0,000	829,065	0,300	-4,488	7,181E-06
CPIJ019550	Predicted protein		-1,346	0,049	18,890	0,497	-2,709	6,745E-03
CPIJ002909	Far upstream binding protein		-1,346	0,027	29,522	0,453	-2,968	2,993E-03
CPIJ010503	Ankyrin 2,3/unc44		-1,345	0,075	16,684	0,538	-2,497	1,253E-02
CPIJ007524	Putative uncharacterized protein	Gamma-interferon inducible lysosor	-1,343	0,007	143,450	0,384	-3,498	4,696E-04
CPIJ008352	Putative uncharacterized protein	N-acetyltransferase domain-containi	-1,343	0,049	16,027	0,495	-2,715	6,629E-03
CPIJ016922	farnesol dehydrogenase		-1,338	0,023	40,369	0,440	-3,043	2,341E-03
CPIJ002334	Importin-4		-1,337	0,050	31,545	0,496	-2,693	7,092E-03
CPIJ019789	Putative uncharacterized protein		-1,336	0,007	33,823	0,384	-3,479	5,036E-04
CPIJ009811	Mitochondrial ribosomal protein L38		-1,335	0,168	10,384	0,653	-2,044	4,095E-02
CPIJ009807	Bhlhzip transcription factor bigmax		-1,332	0,115	12,740	0,587	-2,269	2,327E-02
CPIJ014505	Putative uncharacterized protein	NADH dehydrogenase [ubiquinone]	-1,331	0,193	7,588	0,683	-1,948	5,136E-02
CPIJ004642	Trypsin		-1,330	0,062	14,982	0,513	-2,593	9,507E-03
CPIJ005015	ICLn protein		-1,330	0,311	3,910	0,835	-1,593	1,112E-01
CPIJ006926	protein phosphatase 1 regulatory subunit 12b		-1,324	0,282	4,590	0,794	-1,669	9,514E-02
CPIJ009933	Adenylate kinase isoenzyme 6 homolog		-1,323	0,138	10,459	0,610	-2,169	3,006E-02
CPIJ004871	Putative uncharacterized protein	DNA-directed RNA polymerase	-1,320	0,189	8,791	0,671	-1,966	4,933E-02
CPIJ011274	Putative uncharacterized protein	Cytochrome oxidase complex assem	-1,320	0,256	5,826	0,757	-1,742	8,144E-02
CPIJ016095	mediator complex, subunit, putative		-1,317	0,096	12,476	0,556	-2,367	1,793E-02
CPIJ008693	Putative uncharacterized protein	Neurochondrin/leucine-rich protein	-1,316	0,166	8,680	0,641	-2,053	4,007E-02
CPIJ003160	Pre-mRNA splicing factor ATP-dependent RNA helicase PRP16		-1,313	0,169	7,877	0,644	-2,040	4,134E-02
CPIJ007989	Porphobilinogen deaminase		-1,313	0,204	6,958	0,689	-1,906	5,670E-02
CPIJ003826	serine proteases 1/2 precursor		-1,307	0,149	1046,153	0,616	-2,123	3,377E-02
CPIJ009956	D-amino acid oxidase		-1,304	0,046	17,909	0,477	-2,736	6,227E-03
CPIJ020105	apolipoprotein D, putative		-1,302	0,212	7,490	0,692	-1,882	5,985E-02

CPIJ002644	28S ribosomal protein S18b, mitochondrial precursor		-1,302	0,132	12,230	0,594	-2,194	2,824E-02
CPIJ015650	Sulfotransferase 1A1		-1,301	0,056	17,932	0,493	-2,638	8,338E-03
CPIJ000730	Arginyl-tRNA synthetase		-1,296	0,006	40,461	0,368	-3,526	4,221E-04
CPIJ011934	Succinyl-CoA:3-ketoacid-coenzyme A transferase		-1,295	0,051	19,891	0,482	-2,686	7,221E-03
CPIJ009344	Putative uncharacterized protein	Transmembrane protein 186	-1,295	0,159	8,783	0,621	-2,084	3,712E-02
CPIJ002450	chaperonin		-1,293	0,004	71,554	0,352	-3,678	2,352E-04
CPIJ007145	Neurobeachin		-1,292	0,026	34,923	0,432	-2,989	2,800E-03
CPIJ006776	Hematopoietic signal peptide-containing membrane domain-containing 1		-1,292	0,027	23,230	0,434	-2,976	2,920E-03
CPIJ011727	Translocon-associated protein subunit gamma		-1,291	0,000	160,047	0,244	-5,299	1,164E-07
CPIJ007468	Putative uncharacterized protein	Translation machinery-associated pr	-1,289	0,026	23,005	0,431	-2,990	2,793E-03
CPIJ014285	3-oxoacyl-[acyl-carrier-protein] reductase		-1,286	0,149	10,721	0,606	-2,124	3,371E-02
CPIJ009348	Neurotransmitter gated ion channel		-1,286	0,202	7,025	0,671	-1,915	5,545E-02
CPIJ013728	GM130		-1,285	0,130	11,532	0,583	-2,203	2,756E-02
CPIJ007217	speckle-type poz protein		-1,285	0,191	8,449	0,657	-1,956	5,045E-02
CPIJ012182	Putative uncharacterized protein	CCAAT-binding factor domain-con	-1,282	0,178	9,103	0,640	-2,003	4,517E-02
CPIJ005886	polyribonucleotide nucleotidyltransferase 1, mitochondrial precursor		-1,282	0,259	5,466	0,740	-1,733	8,308E-02
CPIJ017452	lysyl-tRNA synthetase		-1,275	0,021	43,392	0,414	-3,081	2,064E-03
CPIJ011412	M-phase phosphoprotein 11		-1,272	0,101	14,034	0,544	-2,339	1,936E-02
CPIJ005347	26S proteasome non-ATPase regulatory subunit 13		-1,272	0,090	16,984	0,529	-2,406	1,612E-02
CPIJ015730	apolipoprotein D, putative		-1,270	0,000	358,133	0,257	-4,950	7,433E-07
CPIJ011993	sodium/potassium-dependent atpase beta-2 subunit		-1,268	0,398	3,150	0,914	-1,388	1,652E-01
CPIJ015102	anionic trypsin-2 precursor		-1,268	0,008	48,589	0,370	-3,427	6,106E-04
CPIJ014590	Putative uncharacterized protein	ER membrane protein complex subu	-1,267	0,002	58,454	0,325	-3,896	9,772E-05
CPIJ014972	protein FAM20B precursor		-1,266	0,273	5,431	0,747	-1,694	9,032E-02
CPIJ016525	Histidyl-tRNA synthetase		-1,261	0,044	26,102	0,457	-2,760	5,783E-03
CPIJ010121	Copper-transporting ATPase 1		-1,260	0,258	6,511	0,726	-1,737	8,246E-02
CPIJ008476	Putative uncharacterized protein	RRM domain-containing protein	-1,259	0,033	22,841	0,436	-2,889	3,866E-03
CPIJ017753	activin receptor type ii		-1,258	0,310	4,527	0,789	-1,595	1,107E-01
CPIJ006024	Putative uncharacterized protein	Macoilin	-1,254	0,303	4,633	0,777	-1,613	1,067E-01
CPIJ000861	protein shuttle craft		-1,251	0,012	210,012	0,382	-3,276	1,054E-03
CPIJ009707	Putative uncharacterized protein	Nuclear envelope integral membran	-1,245	0,356	3,912	0,841	-1,481	1,387E-01
CPIJ011854	CD98hc amino acid transporter protein		-1,243	0,049	37,288	0,460	-2,703	6,863E-03
CPIJ006730	ATP-dependent RNA helicase DDX55		-1,242	0,130	11,425	0,564	-2,203	2,759E-02
CPIJ003821	Sel1l protein		-1,241	0,000	114,328	0,278	-4,462	8,110E-06
CPIJ007629	splicing factor, putative		-1,241	0,378	39,108	0,867	-1,431	1,524E-01
CPIJ014438	Sex-determining protein fem-1		-1,241	0,357	3,752	0,840	-1,478	1,395E-01
CPIJ005754	B230208H17Rik protein		-1,240	0,355	3,926	0,835	-1,485	1,376E-01
CPIJ002958	sterol o-acyltransferase		-1,239	0,089	16,805	0,514	-2,414	1,579E-02

CPIJ018833	H/ACA ribonucleoprotein complex subunit 3		-1,239	0,024	25,258	0,410	-3,020	2,526E-03
CPIJ016096	Ciao1: protein CIAO1		-1,236	0,202	9,835	0,647	-1,912	5,594E-02
CPIJ000243	otefin, putative		-1,235	0,325	4,704	0,793	-1,558	1,193E-01
CPIJ011259	Eukaryotic translation initiation factor		-1,234	0,001	67,382	0,302	-4,091	4,303E-05
CPIJ012635	protein bicaudal D		-1,234	0,331	4,539	0,799	-1,544	1,225E-01
CPIJ006357	Putative uncharacterized protein	C2H2-type domain-containing prote	-1,233	0,425	3,152	0,931	-1,324	1,853E-01
CPIJ000513	Nuclear pore complex protein Nup155		-1,230	0,179	9,978	0,615	-2,000	4,555E-02
CPIJ000716	ion channel nompc		-1,228	0,015	47,061	0,384	-3,198	1,382E-03
CPIJ003795	Oxidoreductase		-1,227	0,094	18,366	0,515	-2,383	1,719E-02
CPIJ014970	niemann-Pick C1 protein precursor		-1,223	0,000	164,799	0,241	-5,083	3,718E-07
CPIJ013678	Putative uncharacterized protein		-1,222	0,421	3,260	0,917	-1,333	1,824E-01
CPIJ003550	78 kDa glucose-regulated protein precursor		-1,222	0,010	517,779	0,364	-3,352	8,030E-04
CPIJ002873	Breast cancer metastasis-suppressor 1		-1,220	0,324	4,566	0,782	-1,561	1,185E-01
CPIJ004173	Juvenile hormone-inducible protein, putative		-1,219	0,001	68,760	0,305	-3,994	6,490E-05
CPIJ003850	splicing factor pTSR1, putative		-1,218	0,212	7,621	0,648	-1,880	6,014E-02
CPIJ014085	Nucleoporin-17		-1,217	0,300	5,213	0,750	-1,623	1,046E-01
CPIJ009629	Zinc finger X-linked protein ZXDA		-1,216	0,218	7,544	0,653	-1,861	6,270E-02
CPIJ003711	E3 ubiquitin-protein ligase MARCH6		-1,215	0,218	8,742	0,653	-1,861	6,271E-02
CPIJ002043	Translocon-associated protein, delta subunit		-1,214	0,031	24,998	0,417	-2,911	3,601E-03
CPIJ006601	Putative uncharacterized protein		-1,214	0,075	17,827	0,486	-2,497	1,252E-02
CPIJ019470	3-demethylubiquinone-9 3-methyltransferase		-1,213	0,007	72,945	0,347	-3,498	4,689E-04
CPIJ015476	Aspartyl-tRNA synthetase		-1,213	0,015	71,849	0,380	-3,196	1,392E-03
CPIJ017662	Peroxisome biogenesis factor 1		-1,210	0,026	27,115	0,404	-2,993	2,765E-03
CPIJ008679	Oligosaccharyl transferase		-1,210	0,000	130,191	0,282	-4,296	1,736E-05
CPIJ005062	Alpha-amylase		-1,210	0,182	1002,886	0,607	-1,991	4,643E-02
CPIJ016740	Putative uncharacterized protein	Thioesterase domain-containing pro	-1,209	0,147	11,716	0,567	-2,133	3,296E-02
CPIJ017177	Utp14a protein		-1,208	0,228	7,541	0,661	-1,828	6,762E-02
CPIJ002941	high choriolytic enzyme 1 precursor		-1,207	0,002	189,132	0,309	-3,908	9,321E-05
CPIJ013995	Putative uncharacterized protein		-1,205	0,259	6,769	0,696	-1,731	8,351E-02
CPIJ002337	Ribonucleoside-diphosphate reductase small chain		-1,202	0,025	33,096	0,400	-3,004	2,661E-03
CPIJ017344	Cathepsin l		-1,201	0,294	5,869	0,734	-1,637	1,017E-01
CPIJ019193	Dihydroceramide delta(4)-desaturase		-1,201	0,002	70,170	0,314	-3,828	1,290E-04
CPIJ015513	Putative uncharacterized protein	Cation/H+ exchanger domain-contai	-1,200	0,439	3,282	0,927	-1,294	1,955E-01
CPIJ013401	Mical		-1,196	0,047	22,165	0,439	-2,727	6,398E-03
CPIJ001411	histone h3 methyltransferase		-1,195	0,313	5,273	0,753	-1,587	1,125E-01
CPIJ003659	peptidyl-tRNA hydrolase 2, mitochondrial precursor		-1,194	0,189	9,522	0,608	-1,965	4,936E-02
CPIJ001197	Golgi-specific brefeldin a-resistance factor		-1,192	0,021	37,487	0,388	-3,073	2,118E-03
CPIJ015212	Aspartyl-tRNA synthetase		-1,192	0,008	59,575	0,346	-3,449	5,634E-04
CPIJ012341	Malic enzyme		-1,192	0,000	121,755	0,276	-4,311	1,624E-05

CPIJ012085	SF21		-1,187	0,063	22,688	0,461	-2,577	9,976E-03
CPIJ012130	Putative uncharacterized protein	Signal recognition particle 19 kDa p	-1,187	0,120	13,243	0,529	-2,245	2,476E-02
CPIJ006918	Putative uncharacterized protein		-1,186	0,043	22,866	0,429	-2,765	5,700E-03
CPIJ009186	Complement component		-1,185	0,005	63,177	0,328	-3,607	3,097E-04
CPIJ011590	Putative uncharacterized protein	DUF4780 domain-containing protei	-1,184	0,079	23,914	0,480	-2,467	1,363E-02
CPIJ011246	Heat shock protein 83		-1,183	0,007	69,131	0,338	-3,506	4,556E-04
CPIJ013940	60S ribosomal protein L6		-1,183	0,198	535,843	0,612	-1,932	5,340E-02
CPIJ006470	NADH:ubiquinone dehydrogenase, putative		-1,181	0,198	10,092	0,611	-1,932	5,341E-02
CPIJ011714	laminin subunit beta-1 precursor		-1,179	0,167	13,209	0,576	-2,049	4,051E-02
CPIJ006316	Putative uncharacterized protein	Interferon-related developmental re	-1,178	0,106	15,484	0,509	-2,313	2,072E-02
CPIJ006169	WD repeat protein 74		-1,177	0,179	11,110	0,589	-2,001	4,544E-02
CPIJ013525	Putative uncharacterized protein	SNF2 N-terminal domain-containing	-1,177	0,056	26,914	0,446	-2,642	8,239E-03
CPIJ003817	Putative uncharacterized protein	Sugar phosphate transporter domain	-1,177	0,096	18,037	0,495	-2,375	1,755E-02
CPIJ011857	Putative uncharacterized protein	Cytochrome b5 heme-binding doma	-1,175	0,348	4,408	0,782	-1,502	1,331E-01
CPIJ001183	39S ribosomal protein L41, mitochondrial precursor		-1,173	0,202	9,272	0,612	-1,915	5,547E-02
CPIJ005508	DNA-J/hsp40		-1,171	0,001	101,565	0,288	-4,060	4,901E-05
CPIJ004492	sodium/potassium/calcium exchanger 3 precursor		-1,169	0,061	27,700	0,450	-2,600	9,328E-03
CPIJ017446	Transmembrane protein 129		-1,168	0,171	12,273	0,575	-2,033	4,209E-02
CPIJ002784	eukaryotic translation initiation factor 3 subunit 10		-1,168	0,009	94,814	0,343	-3,402	6,680E-04
CPIJ003608	glycerol-3-phosphate dehydrogenase, mitochondrial precursor		-1,164	0,032	32,468	0,400	-2,908	3,638E-03
CPIJ009320	Ethanolamine-phosphate cytidylyltransferase		-1,164	0,068	33,916	0,458	-2,544	1,097E-02
CPIJ015964	Brain protein 16		-1,163	0,324	5,224	0,745	-1,562	1,184E-01
CPIJ008453	Putative uncharacterized protein		-1,162	0,275	6,512	0,688	-1,689	9,125E-02
CPIJ007376	Calreticulin		-1,160	0,016	516,016	0,364	-3,188	1,430E-03
CPIJ000771	trypsin-2 precursor		-1,159	0,018	546,584	0,369	-3,138	1,701E-03
CPIJ003279	Tryptophanyl-tRNA synthetase		-1,159	0,002	80,430	0,299	-3,883	1,032E-04
CPIJ040862	Putative uncharacterized protein	Citrate synthase	-1,159	0,028	44,429	0,391	-2,962	3,061E-03
CPIJ006575	cue: low-density lipoprotein receptor		-1,157	0,044	28,285	0,420	-2,754	5,886E-03
CPIJ000318	cytochrome b5, putative		-1,157	0,457	25,215	0,923	-1,253	2,101E-01
CPIJ010762	glucosyl/glucuronosyl transferases		-1,156	0,192	10,432	0,591	-1,955	5,058E-02
CPIJ004817	Sodium-dependent phosphate transporter		-1,155	0,005	70,065	0,319	-3,617	2,981E-04
CPIJ008317	TBC1 domain family member 22B		-1,155	0,227	8,639	0,631	-1,830	6,719E-02
CPIJ014041	Putative uncharacterized protein	DRBM domain-containing protein	-1,155	0,100	18,444	0,493	-2,341	1,922E-02
CPIJ001621	molybdenum cofactor synthesis protein 3		-1,154	0,096	15,945	0,486	-2,373	1,766E-02
CPIJ008786	Arginine/serine-rich splicing factor		-1,151	0,087	18,022	0,476	-2,421	1,547E-02
CPIJ004077	3-hydroxy-3-methylglutaryl-coenzyme A reductase		-1,151	0,209	10,557	0,609	-1,890	5,879E-02
CPIJ012560	Leucine-rich repeat serine/threonine-protein kinase 1		-1,149	0,372	4,257	0,795	-1,445	1,486E-01
CPIJ009498	Adrenodoxin		-1,146	0,005	76,084	0,316	-3,622	2,922E-04

CPIJ015641	RNA-binding motif protein		-1,145	0,259	7,943	0,660	-1,734	8,283E-02
CPIJ009790	Putative uncharacterized protein	Tetratricopeptide repeat protein 39E	-1,144	0,077	20,817	0,461	-2,480	1,316E-02
CPIJ008180	Putative uncharacterized protein		-1,142	0,065	35,035	0,446	-2,564	1,036E-02
CPIJ000736	chromatin assembly factor-I p150 subunit, putative		-1,141	0,217	9,306	0,612	-1,864	6,234E-02
CPIJ012850	Splicing factor u2af large subunit		-1,137	0,049	28,996	0,421	-2,704	6,860E-03
CPIJ019926	Uncharacterized protein (Fragment)		-1,137	0,204	9,404	0,596	-1,907	5,647E-02
CPIJ005163	Putative uncharacterized protein	5-formyltetrahydrofolate cyclo-ligas	-1,136	0,116	20,209	0,501	-2,266	2,347E-02
CPIJ004697	Putative uncharacterized protein		-1,134	0,247	7,956	0,640	-1,771	7,661E-02
CPIJ000466	Putative uncharacterized protein	TLDc domain-containing protein	-1,132	0,075	20,635	0,453	-2,497	1,254E-02
CPIJ002542	Mitochondrial import receptor subunit tom40		-1,129	0,063	25,967	0,438	-2,576	9,994E-03
CPIJ004982	Rho guanine exchange factor		-1,129	0,335	5,801	0,735	-1,535	1,248E-01
CPIJ001445	eukaryotic translation initiation factor 3 subunit		-1,129	0,100	25,799	0,481	-2,346	1,898E-02
CPIJ014581	aldehyde dehydrogenase, mitochondrial		-1,128	0,000	952,940	0,223	-5,053	4,342E-07
CPIJ001894	Prolyl endopeptidase		-1,128	0,152	15,424	0,534	-2,110	3,482E-02
CPIJ001612	heterogeneous nuclear ribonucleoprotein, putative		-1,127	0,007	69,557	0,323	-3,487	4,890E-04
CPIJ006465	Smap1		-1,123	0,246	9,703	0,632	-1,777	7,551E-02
CPIJ013753	Small glutamine-rich tetratricopeptide repeat-containing protein A		-1,121	0,069	23,946	0,442	-2,535	1,125E-02
CPIJ005390	Putative uncharacterized protein	TP53-regulated inhibitor of apoptosi	-1,121	0,186	11,525	0,568	-1,973	4,845E-02
CPIJ003856	Zinc finger protein		-1,120	0,444	3,809	0,873	-1,282	1,998E-01
CPIJ014132	CGI-07 protein		-1,119	0,164	20,426	0,543	-2,061	3,929E-02
CPIJ009684	Mitochondrial 28S ribosomal protein S14		-1,117	0,231	9,864	0,615	-1,817	6,919E-02
CPIJ005690	ASNA1: arsenical pump-driving ATPase		-1,117	0,042	26,529	0,401	-2,784	5,371E-03
CPIJ019666	tRNA pseudouridine synthase A		-1,116	0,380	4,367	0,783	-1,426	1,539E-01
CPIJ005084	Coiled-coil domain-containing protein 124		-1,116	0,164	12,706	0,541	-2,062	3,925E-02
CPIJ008812	Sodium-dependent phosphate transporter		-1,115	0,046	76,991	0,407	-2,740	6,146E-03
CPIJ008853	Maltose phosphorylase		-1,115	0,000	172,841	0,262	-4,263	2,018E-05
CPIJ009445	DEAD box ATP-dependent RNA helicase		-1,115	0,008	287,446	0,325	-3,432	5,984E-04
CPIJ017609	CYP6CD3: cytochrome P450 6CD3		-1,115	0,092	19,811	0,466	-2,393	1,673E-02
CPIJ004309	Nucleolar essential protein 1		-1,113	0,262	7,856	0,645	-1,725	8,449E-02
CPIJ009003	Putative uncharacterized protein	Sulfiredoxin	-1,111	0,136	14,250	0,510	-2,178	2,938E-02
CPIJ008447	Amine oxidase		-1,111	0,052	31,187	0,415	-2,679	7,381E-03
CPIJ003754	Putative uncharacterized protein	FIP-RBD domain-containing proteir	-1,111	0,190	11,246	0,566	-1,962	4,977E-02
CPIJ002430	Cytidine deaminase		-1,110	0,016	53,272	0,350	-3,173	1,509E-03
CPIJ005137	Sec61 protein complex gamma subunit, putative		-1,110	0,000	273,315	0,223	-4,970	6,679E-07
CPIJ006347	actin binding protein, putative		-1,106	0,037	38,003	0,389	-2,845	4,437E-03
CPIJ006777	Putative uncharacterized protein	Large ribosomal subunit protein mL	-1,105	0,158	16,524	0,530	-2,087	3,692E-02
CPIJ000204	Putative uncharacterized protein	Folliculin	-1,104	0,336	5,786	0,722	-1,530	1,259E-01
CPIJ005349	lysosomal acid lipase, putative		-1,102	0,338	5,946	0,723	-1,525	1,274E-01

CPIJ012005	Putative uncharacterized protein		-1,102	0,225	10,348	0,599	-1,839	6,594E-02
CPIJ013961	GPI mannosyltransferase 3		-1,102	0,145	14,793	0,515	-2,140	3,232E-02
CPIJ014059	NADP-dependent leukotriene B4 12-hydroxydehydrogenase		-1,102	0,154	14,992	0,524	-2,101	3,566E-02
CPIJ009515	Gp210		-1,101	0,141	14,705	0,511	-2,156	3,104E-02
CPIJ001140	Rhomboid-like protein		-1,099	0,483	2,954	0,921	-1,193	2,330E-01
CPIJ010486	glycyl-tRNA synthetase		-1,098	0,049	45,879	0,406	-2,702	6,889E-03
CPIJ002096	Importin-7		-1,096	0,018	70,193	0,349	-3,141	1,682E-03
CPIJ003570	Putative uncharacterized protein	Mitochondrial import inner membra	-1,095	0,189	12,499	0,557	-1,966	4,928E-02
CPIJ011277	Vacuolar protein sorting-associated protein 26B-B		-1,093	0,193	11,843	0,561	-1,948	5,144E-02
CPIJ003395	Reductase protein		-1,092	0,001	167,734	0,258	-4,232	2,321E-05
CPIJ011244	Heat shock protein 83		-1,091	0,051	32,946	0,406	-2,685	7,242E-03
CPIJ019831	Putative uncharacterized protein	Methyltransferase type 11 domain-c	-1,091	0,291	7,170	0,664	-1,643	1,003E-01
CPIJ018629	Glutathione-s-transferase theta, gst		-1,091	0,000	315,815	0,246	-4,426	9,619E-06
CPIJ004406	Coatomer		-1,090	0,057	31,508	0,414	-2,634	8,429E-03
CPIJ015801	mitogen activated protein kinase kinase 2, mapkk2, mek2		-1,087	0,298	6,867	0,668	-1,626	1,039E-01
CPIJ000241	Ubiquitin carboxyl-terminal hydrolase 43		-1,087	0,221	10,459	0,586	-1,853	6,383E-02
CPIJ020331	Oxidoreductase (Fragment)		-1,087	0,186	18,853	0,550	-1,977	4,805E-02
CPIJ015685	3-oxoacyl-[acyl-carrier-protein] reductase		-1,084	0,492	3,014	0,923	-1,175	2,398E-01
CPIJ009423	WD repeat protein		-1,084	0,275	8,446	0,643	-1,687	9,156E-02
CPIJ011366	Mitochondrial ribosomal protein S31		-1,081	0,154	14,884	0,514	-2,102	3,556E-02
CPIJ007987	ATP-dependent RNA helicase DRS1		-1,079	0,054	29,572	0,406	-2,657	7,888E-03
CPIJ013673	mRpL11: 39S ribosomal protein L11, mitochondrial		-1,079	0,172	14,386	0,532	-2,027	4,264E-02
CPIJ006946	Prosalpha2: 26S proteasome alpha 2 subunit		-1,079	0,199	13,344	0,560	-1,927	5,400E-02
CPIJ017520	U3 small nucleolar ribonucleoprotein protein IMP3		-1,079	0,104	18,849	0,464	-2,322	2,021E-02
CPIJ005343	glucosylceramidase precursor		-1,077	0,160	13,333	0,518	-2,079	3,765E-02
CPIJ009281	Sec24B protein, putative		-1,077	0,071	41,726	0,427	-2,523	1,162E-02
CPIJ010002	T-complex protein 1 subunit gamma		-1,075	0,096	24,134	0,454	-2,367	1,793E-02
CPIJ013616	trypsin 5 precursor		-1,075	0,246	14,020	0,605	-1,775	7,585E-02
CPIJ004080	T-complex protein 1 subunit alpha		-1,071	0,026	71,844	0,357	-3,000	2,701E-03
CPIJ006599	mannosyl-oligosaccharide alpha-1,2-mannosidase, putative		-1,068	0,059	24,886	0,408	-2,616	8,907E-03
CPIJ014804	ATP-dependent RNA helicase A		-1,067	0,226	10,527	0,581	-1,836	6,638E-02
CPIJ002452	ubiquitin thioesterase OTUB1		-1,066	0,161	14,987	0,514	-2,073	3,818E-02
CPIJ016405	protein FAM18B		-1,066	0,172	13,876	0,526	-2,028	4,261E-02
CPIJ015634	Putative uncharacterized protein		-1,065	0,217	13,765	0,572	-1,863	6,249E-02
CPIJ012476	Putative uncharacterized protein	N-acetyltransferase domain-containi	-1,065	0,289	7,642	0,646	-1,649	9,917E-02
CPIJ002123	Rsl1d1 protein		-1,065	0,144	19,603	0,496	-2,145	3,195E-02
CPIJ001807	Carbonic anhydrase		-1,065	0,018	49,623	0,339	-3,143	1,675E-03
CPIJ002098	chymotrypsin BI precursor		-1,064	0,365	5,376	0,728	-1,461	1,439E-01

CPIJ014696	Four way stop		-1,063	0,251	8,958	0,605	-1,759	7,861E-02
CPIJ008184	60S ribosomal protein L35a		-1,063	0,109	52,711	0,463	-2,297	2,162E-02
CPIJ007348	Nuclear movement protein nudC		-1,063	0,249	9,593	0,601	-1,767	7,720E-02
CPIJ011127	CYP4H34: cytochrome P450 4H34		-1,062	0,058	83,729	0,404	-2,626	8,638E-03
CPIJ004890	Putative uncharacterized protein	Ribosomal protein S4/S9 N-terminal	-1,061	0,492	34,447	0,903	-1,175	2,401E-01
CPIJ008163	Skeletal muscle/kidney enriched inositol 5-phosphatase		-1,061	0,212	12,456	0,564	-1,882	5,985E-02
CPIJ013965	cation efflux protein/ zinc transporter, putative		-1,061	0,104	33,066	0,456	-2,324	2,011E-02
CPIJ004664	Putative uncharacterized protein	6-phosphofructo-2-kinase domain-co	-1,060	0,044	35,919	0,384	-2,759	5,796E-03
CPIJ004572	O-linked N-acetylglucosamine transferase, ogt		-1,057	0,201	11,185	0,551	-1,919	5,497E-02
CPIJ018608	Putative uncharacterized protein	KH homology domain-containing pr	-1,057	0,457	3,545	0,843	-1,253	2,101E-01
CPIJ013329	peptidyl-prolyl cis-trans isomerase NIMA-interacting 4		-1,055	0,160	15,089	0,508	-2,077	3,776E-02
CPIJ003176	Putative uncharacterized protein		-1,055	0,160	15,998	0,508	-2,077	3,779E-02
CPIJ010578	Putative uncharacterized protein	C2H2-type domain-containing prote	-1,053	0,175	14,980	0,522	-2,017	4,374E-02
CPIJ000602	sulfate transporter, putative		-1,053	0,367	6,323	0,723	-1,457	1,451E-01
CPIJ008675	Diphthine synthase		-1,050	0,253	12,200	0,599	-1,752	7,973E-02
CPIJ015409	Cleavage and polyadenylation specificity factor		-1,050	0,204	11,997	0,551	-1,908	5,641E-02
CPIJ002953	FACT complex subunit SSRP1		-1,050	0,246	9,541	0,592	-1,775	7,591E-02
CPIJ001676	CDP-diacylglycerol-glycerol-3-phosphate 3-phosphatidyltransferase		-1,050	0,302	7,465	0,650	-1,616	1,062E-01
CPIJ017562	39S ribosomal protein L22, mitochondrial precursor		-1,049	0,232	12,245	0,578	-1,814	6,974E-02
CPIJ001516	Putative uncharacterized protein		-1,048	0,339	6,671	0,688	-1,522	1,279E-01
CPIJ007073	protein l(2)37Cc		-1,048	0,002	94,856	0,270	-3,882	1,036E-04
CPIJ013454	Sec63		-1,047	0,076	41,912	0,421	-2,485	1,295E-02
CPIJ005564	26S proteasome non-ATPase regulatory subunit 9		-1,046	0,301	7,725	0,646	-1,619	1,054E-01
CPIJ012539	Organic cation transporter protein		-1,045	0,096	26,185	0,442	-2,367	1,792E-02
CPIJ007229	tyrosyl-tRNA synthetase		-1,041	0,037	38,536	0,367	-2,839	4,532E-03
CPIJ009363	U3 small nucleolar ribonucleoprotein protein MPP10		-1,041	0,197	14,296	0,537	-1,937	5,279E-02
CPIJ006480	40S ribosomal protein S26		-1,041	0,043	402,710	0,377	-2,763	5,725E-03
CPIJ016441	Ubiquitin carboxyl-terminal hydrolase		-1,039	0,017	59,324	0,328	-3,165	1,553E-03
CPIJ013752	Acyl-CoA Delta(11) desaturase		-1,039	0,000	188,310	0,240	-4,328	1,506E-05
CPIJ000912	Peptidyl-prolyl cis-trans isomerase cyp6		-1,039	0,254	10,825	0,594	-1,748	8,044E-02
CPIJ003981	15 kDa selenoprotein precursor		-1,037	0,124	20,712	0,465	-2,230	2,578E-02
CPIJ012743	60S ribosomal protein L7		-1,037	0,168	24,744	0,508	-2,042	4,117E-02
CPIJ017243	CYP304B4: cytochrome P450 304B4		-1,037	0,049	82,997	0,383	-2,709	6,743E-03
CPIJ000857	Putative uncharacterized protein		-1,036	0,398	4,764	0,748	-1,384	1,663E-01
CPIJ017744	Sterol desaturase		-1,035	0,400	4,966	0,750	-1,380	1,677E-01
CPIJ005612	whd: protein withered, carnitine O-palmitoyltransferase		-1,035	0,037	85,244	0,364	-2,839	4,524E-03
CPIJ007693	Putative uncharacterized protein	DUF3456 domain-containing protein	-1,034	0,041	113,505	0,370	-2,794	5,198E-03
CPIJ002780	Sur-8: leucine-rich repeat protein SHOC-2		-1,034	0,300	7,786	0,637	-1,622	1,049E-01

CPIJ010328	39S ribosomal protein L51, mitochondrial precursor		-1,033	0,275	8,998	0,612	-1,688	9,135E-02
CPIJ005552	thioredoxin reductase 1, mitochondrial precursor		-1,032	0,027	74,678	0,348	-2,970	2,974E-03
CPIJ011652	Multidrug resistance-associated protein 1		-1,032	0,231	13,705	0,568	-1,817	6,928E-02
CPIJ003802	NADP-dependent leukotriene B4 12-hydroxydehydrogenase		-1,030	0,030	217,856	0,351	-2,936	3,320E-03
CPIJ013113	Putative uncharacterized protein	Small integral membrane protein 8	-1,026	0,417	4,889	0,763	-1,343	1,791E-01
CPIJ009694	tRNA (guanine-N(7)-)-methyltransferase		-1,025	0,346	7,216	0,680	-1,508	1,316E-01
CPIJ010781	secreted ferritin G subunit precursor, putative		-1,025	0,285	14,026	0,618	-1,659	9,721E-02
CPIJ016574	Geranylgeranyl transferase type-1 subunit beta		-1,023	0,238	12,615	0,570	-1,796	7,256E-02
CPIJ010187	Sodium/solute symporter		-1,022	0,671	6,791	1,288	-0,793	4,275E-01
CPIJ003512	defender against cell death 1		-1,022	0,023	45,535	0,336	-3,039	2,373E-03
CPIJ012562	26S proteasome non-ATPase regulatory subunit 10		-1,022	0,446	4,210	0,802	-1,274	2,028E-01
CPIJ005868	Multisynthetase complex auxiliary component p43		-1,021	0,096	28,774	0,430	-2,374	1,761E-02
CPIJ012803	Folate carrier protein		-1,020	0,078	26,921	0,413	-2,473	1,339E-02
CPIJ016471	Signal transducer and activator of transcription		-1,019	0,185	17,396	0,515	-1,979	4,781E-02
CPIJ010680	Putative uncharacterized protein	Mitochondrial 28s ribosomal proteir	-1,019	0,166	18,073	0,497	-2,050	4,037E-02
CPIJ002088	Ubiquitin-related modifier 1 homolog		-1,018	0,157	16,816	0,487	-2,091	3,654E-02
CPIJ003552	Putative uncharacterized protein	Nicalin	-1,018	0,007	70,816	0,294	-3,463	5,338E-04
CPIJ010738	protein FAM96A		-1,018	0,125	22,174	0,458	-2,222	2,631E-02
CPIJ011856	E3 ubiquitin-protein ligase MARCH5		-1,017	0,185	20,524	0,514	-1,981	4,761E-02
CPIJ005483	Putative uncharacterized protein	EGF-like domain-containing protein	-1,017	0,156	16,844	0,486	-2,093	3,638E-02
CPIJ004548	mitochondrial carrier protein, putative		-1,013	0,330	7,274	0,654	-1,548	1,216E-01
CPIJ002691	Small nuclear ribonucleoprotein F		-1,011	0,171	14,585	0,497	-2,032	4,217E-02
CPIJ002169	Adenine phosphoribosyltransferase		-1,009	0,041	34,735	0,361	-2,796	5,172E-03
CPIJ007213	Putative uncharacterized protein	Major royal jelly protein	-1,009	0,175	19,291	0,500	-2,017	4,372E-02
CPIJ007047	Serine/arginine rich splicing factor		-1,008	0,109	24,251	0,439	-2,294	2,179E-02
CPIJ008648	Yemanuclein		-1,008	0,186	15,646	0,510	-1,976	4,814E-02
CPIJ007855	Zinc finger protein 667		-1,007	0,352	6,913	0,675	-1,492	1,358E-01
CPIJ011327	Putative uncharacterized protein	HTH CENPB-type domain-containi	-1,006	0,422	4,799	0,755	-1,332	1,828E-01
CPIJ000119	Kelch domain-containing protein 4		-1,006	0,249	18,178	0,569	-1,767	7,723E-02
CPIJ006563	UDP-sugar transporter UST74c		-1,005	0,254	10,962	0,574	-1,750	8,015E-02
CPIJ011219	Vacuolar protein sorting-associated protein 29		-1,004	0,269	9,915	0,588	-1,708	8,756E-02
CPIJ007234	AdoMet-dependent rRNA methyltransferase spb1		-1,004	0,141	27,308	0,466	-2,155	3,120E-02
CPIJ010373	F-actin capping protein subunit beta		-1,004	0,036	37,935	0,352	-2,854	4,317E-03
CPIJ003173	Putative uncharacterized protein	COMM domain-containing protein [illegible]	-1,003	0,440	5,669	0,776	-1,292	1,963E-01
CPIJ014053	Glutathione-s-transferase theta, gst		-1,003	0,079	28,687	0,406	-2,470	1,350E-02
CPIJ019500	Putative uncharacterized protein	ADF-H domain-containing protein	-1,003	0,063	31,224	0,388	-2,585	9,751E-03
CPIJ003801	NADP-dependent leukotriene B4 12-hydroxydehydrogenase		-1,002	0,031	44,302	0,343	-2,919	3,507E-03
CPIJ017179	scaffold attachment factor B, putative		-1,002	0,132	19,811	0,456	-2,197	2,805E-02

CPIJ001998	1-acyl-sn-glycerol-3-phosphate acyltransferase		1,002	0,050	31,342	0,372	2,696	7,016E-03
CPIJ002130	kallikrein-7 precursor		1,005	0,243	19,065	0,563	1,785	7,421E-02
CPIJ007311	Putative uncharacterized protein	5-oxoprolinase	1,011	0,001	119,770	0,255	3,972	7,122E-05
CPIJ000050	Carboxylesterase		1,012	0,015	70,484	0,315	3,211	1,325E-03
CPIJ015919	Putative uncharacterized protein	CHK kinase-like domain-containing	1,014	0,051	56,901	0,377	2,689	7,163E-03
CPIJ009225	Aquaporin		1,017	0,035	49,659	0,354	2,872	4,081E-03
CPIJ000863	6-pyruvoyl tetrahydrobiopterin synthase		1,018	0,056	49,980	0,385	2,644	8,201E-03
CPIJ000317	Putative uncharacterized protein		1,020	0,205	13,531	0,536	1,903	5,706E-02
CPIJ015334	serine/threonine-protein kinase NIM1		1,021	0,202	11,186	0,533	1,915	5,544E-02
CPIJ008797	Putative uncharacterized protein	gamma-glutamylcyclotransferase	1,021	0,065	29,693	0,399	2,561	1,043E-02
CPIJ005974	Putative uncharacterized protein	Alpha 2-macroglobulin receptor-ass	1,022	0,043	31,317	0,369	2,771	5,596E-03
CPIJ007727	Fibrinogen and fibronectin		1,024	0,000	179,131	0,236	4,332	1,475E-05
CPIJ008952	Putative uncharacterized protein		1,025	0,080	25,139	0,416	2,462	1,382E-02
CPIJ009478	CYP4D42: cytochrome P450 4D42		1,027	0,005	280,149	0,287	3,576	3,484E-04
CPIJ004086	Angiotensin-converting enzyme		1,030	0,001	1698,396	0,243	4,240	2,238E-05
CPIJ017918	pseudouridylate synthase		1,031	0,202	11,523	0,538	1,916	5,541E-02
CPIJ015662	Ecto ADP-ribosylhydrolase		1,032	0,026	45,290	0,345	2,988	2,804E-03
CPIJ002536	CYP6AG10: cytochrome P450 6AG10		1,032	0,015	271,214	0,321	3,212	1,317E-03
CPIJ011266	Putative uncharacterized protein	Tetraspanin	1,033	0,200	10,701	0,537	1,923	5,445E-02
CPIJ000017	Putative uncharacterized protein	C3H1-type domain-containing prote	1,035	0,307	5,966	0,646	1,603	1,090E-01
CPIJ019219	Putative uncharacterized protein		1,037	0,176	11,705	0,515	2,013	4,408E-02
CPIJ003945	three prime repair exonuclease 1, putative		1,037	0,020	200,697	0,336	3,091	1,995E-03
CPIJ002140	chymotrypsin BI precursor		1,039	0,006	590,293	0,295	3,520	4,321E-04
CPIJ013934	Xanthine dehydrogenase/oxidase		1,039	0,090	30,887	0,432	2,404	1,623E-02
CPIJ003432	Aldehyde dehydrogenase		1,040	0,005	486,171	0,292	3,562	3,677E-04
CPIJ009306	neutral alpha-glucosidase ab precursor		1,044	0,148	28,341	0,490	2,129	3,326E-02
CPIJ003335	Putative uncharacterized protein	SAM domain-containing protein	1,045	0,178	12,892	0,522	2,003	4,521E-02
CPIJ001939	Putative uncharacterized protein		1,046	0,228	8,885	0,573	1,826	6,783E-02
CPIJ001052	aminopeptidase 2, mitochondrial precursor		1,047	0,006	73,374	0,296	3,533	4,108E-04
CPIJ011721	Putative uncharacterized protein	MIF4G domain-containing protein	1,047	0,121	18,325	0,468	2,240	2,509E-02
CPIJ005824	Maternal tudor protein		1,049	0,019	43,022	0,337	3,112	1,860E-03
CPIJ020219	Putative uncharacterized protein	chloroplast protein-transporting ATI	1,051	0,106	18,667	0,454	2,313	2,075E-02
CPIJ002825	Predicted protein		1,053	0,056	23,888	0,398	2,642	8,248E-03
CPIJ014232	Putative uncharacterized protein	Hemolymph juvenile hormone bindi	1,054	0,141	17,729	0,489	2,155	3,113E-02
CPIJ002537	CYP6AG11: cytochrome P450 6AG11		1,055	0,002	2685,013	0,269	3,923	8,739E-05
CPIJ015049	Putative uncharacterized protein		1,056	0,277	7,634	0,628	1,680	9,288E-02
CPIJ016398	GTP-binding protein (I) alpha subunit		1,056	0,296	6,659	0,647	1,632	1,027E-01
CPIJ005191	leucine-rich repeat-containing protein 15 precursor		1,058	0,001	645,958	0,250	4,228	2,361E-05

CPIJ008005	annexin x		1,058	0,106	20,650	0,458	2,309	2,094E-02
CPIJ008195	Putative uncharacterized protein	RanBP2-type domain-containing pro	1,060	0,120	17,494	0,472	2,246	2,468E-02
CPIJ014576	Putative uncharacterized protein	Cysteine-rich domain-containing pro	1,062	0,019	36,610	0,341	3,114	1,845E-03
CPIJ019722	GTP binding protein		1,063	0,104	16,303	0,457	2,324	2,010E-02
CPIJ017227	phospholipase D3		1,063	0,082	20,150	0,433	2,452	1,422E-02
CPIJ008065	Caspase-1		1,064	0,007	306,393	0,306	3,474	5,122E-04
CPIJ009553	Putative uncharacterized protein		1,065	0,135	14,055	0,489	2,179	2,931E-02
CPIJ012751	apl5 protein		1,071	0,034	31,303	0,372	2,880	3,979E-03
CPIJ008235	Putative uncharacterized protein		1,072	0,294	6,854	0,654	1,638	1,015E-01
CPIJ012628	endophilin a, putative		1,073	0,076	22,595	0,431	2,488	1,284E-02
CPIJ003277	U1 small nuclear ribonucleoprotein C		1,073	0,172	11,359	0,529	2,029	4,250E-02
CPIJ019524	Putative uncharacterized protein		1,073	0,250	8,964	0,610	1,761	7,828E-02
CPIJ010939	Putative uncharacterized protein	Cyclin	1,074	0,042	36,006	0,386	2,783	5,388E-03
CPIJ018151	Phosphatidylinositol 3-kinase regulatory subunit		1,076	0,297	6,418	0,660	1,630	1,032E-01
CPIJ007015	Putative uncharacterized protein	BHLH domain-containing protein	1,076	0,133	13,628	0,492	2,189	2,863E-02
CPIJ005959	CYP6AA7: cytochrome P450 6AA7		1,076	0,000	244,261	0,221	4,872	1,104E-06
CPIJ003134	Formin 1,2/cappuccino		1,078	0,225	8,485	0,586	1,839	6,596E-02
CPIJ000999	Putative uncharacterized protein	Leptin receptor gene-related protein	1,078	0,126	16,107	0,486	2,219	2,646E-02
CPIJ005020	LRIM18: leucine-rich immune protein (Coil-less)		1,078	0,139	13,044	0,498	2,163	3,051E-02
CPIJ009443	Autophagy-specific protein, putative		1,082	0,297	6,702	0,664	1,629	1,033E-01
CPIJ016727	Muskelin		1,082	0,026	33,633	0,361	2,995	2,745E-03
CPIJ006031	Putative uncharacterized protein	BPL/LPL catalytic domain-containi	1,083	0,001	123,457	0,273	3,962	7,425E-05
CPIJ012685	CYP315A1: cytochrome P450 315A1		1,083	0,007	647,069	0,311	3,484	4,948E-04
CPIJ003102	protein croquemort		1,084	0,005	259,542	0,301	3,606	3,114E-04
CPIJ007824	esterase B1 precursor		1,089	0,000	249,635	0,232	4,699	2,615E-06
CPIJ007342	Putative uncharacterized protein	SET domain-containing protein	1,090	0,022	36,408	0,357	3,055	2,254E-03
CPIJ004355	protein krueppel		1,091	0,156	13,041	0,521	2,093	3,638E-02
CPIJ016990	Putative uncharacterized protein		1,092	0,025	32,901	0,362	3,018	2,544E-03
CPIJ010305	CHKov1, putative		1,093	0,001	143,278	0,273	4,006	6,168E-05
CPIJ004088	Guanylyl cyclase receptor		1,093	0,163	12,404	0,529	2,065	3,888E-02
CPIJ010979	phosphatidylcholine-sterol acyltransferase precursor		1,097	0,014	126,308	0,339	3,240	1,194E-03
CPIJ001556	acyl-coenzyme A oxidase 3		1,098	0,001	400,684	0,265	4,142	3,436E-05
CPIJ007625	Alcohol dehydrogenase		1,099	0,268	7,666	0,643	1,709	8,742E-02
CPIJ016771	peptidoglycan recognition protein precursor		1,099	0,010	320,368	0,327	3,357	7,885E-04
CPIJ006516	Putative uncharacterized protein		1,100	0,063	20,332	0,426	2,579	9,899E-03
CPIJ006747	Transient receptor potential cation channel protein painless		1,100	0,137	13,259	0,506	2,174	2,970E-02
CPIJ001380	CYP314A1: cytochrome P450 314A1		1,101	0,011	58,609	0,332	3,319	9,030E-04
CPIJ007566	Mitochondrial cytochrome c1		1,101	0,009	48,847	0,324	3,398	6,779E-04

CPIJ002777	Putative uncharacterized protein	Transmembrane protein 192	1,102	0,002	110,812	0,289	3,814	1,369E-04
CPIJ005955	CYP6P14: cytochrome P450 6P14		1,103	0,000	603,705	0,234	4,705	2,544E-06
CPIJ010921	Monocarboxylate transporter		1,106	0,044	24,136	0,401	2,758	5,820E-03
CPIJ003967	Putative uncharacterized protein	Chitin-binding type-2 domain-conta	1,109	0,001	492,248	0,279	3,972	7,141E-05
CPIJ000124	Putative uncharacterized protein		1,113	0,097	15,388	0,472	2,360	1,828E-02
CPIJ001230	Putative uncharacterized protein		1,117	0,151	11,404	0,528	2,117	3,425E-02
CPIJ013194	Putative uncharacterized protein		1,117	0,032	49,408	0,385	2,900	3,729E-03
CPIJ018721	Putative uncharacterized protein		1,119	0,013	74,779	0,343	3,261	1,112E-03
CPIJ000005	Malate synthase		1,119	0,001	199,564	0,266	4,213	2,522E-05
CPIJ003267	sodium/chloride dependent amino acid transporter		1,119	0,027	73,723	0,377	2,973	2,951E-03
CPIJ005975	Putative uncharacterized protein	EF-hand domain-containing protein	1,122	0,011	901,388	0,339	3,315	9,177E-04
CPIJ005303	erk1/2		1,127	0,273	8,097	0,666	1,693	9,043E-02
CPIJ001670	glycerol-3-phosphate acyltransferase, putative		1,127	0,001	180,745	0,270	4,171	3,032E-05
CPIJ016156	Putative uncharacterized protein	PWWP domain-containing protein	1,128	0,212	7,538	0,600	1,879	6,030E-02
CPIJ015428	CYP6Z10: cytochrome P450 6Z10		1,128	0,000	432,795	0,227	4,977	6,444E-07
CPIJ007581	Vanin-like protein 1 precursor, putative		1,129	0,000	280,152	0,232	4,865	1,142E-06
CPIJ005050	ATP-binding cassette sub-family A member 3		1,130	0,024	38,483	0,373	3,030	2,443E-03
CPIJ008128	Putative uncharacterized protein	Nuclear respiratory factor 1 NLS/DI	1,130	0,023	37,908	0,371	3,044	2,337E-03
CPIJ002518	chymotrypsin 1 precursor		1,133	0,571	17,897	1,136	0,997	3,186E-01
CPIJ016836	Cytochrome c oxidase subunit I		1,135	0,096	15,931	0,479	2,370	1,779E-02
CPIJ017461	Aldo-keto reductase		1,137	0,164	10,572	0,552	2,061	3,934E-02
CPIJ000316	Putative uncharacterized protein		1,137	0,000	177,507	0,266	4,266	1,987E-05
CPIJ004278	Putative uncharacterized protein	Rho-GAP domain-containing protei	1,137	0,120	13,507	0,506	2,246	2,470E-02
CPIJ007938	Asparagine synthetase B		1,142	0,058	37,523	0,435	2,624	8,691E-03
CPIJ010537	CYP9J45: cytochrome P450 9J45		1,142	0,001	395,593	0,275	4,150	3,326E-05
CPIJ016827	26S proteasome regulatory subunit S3		1,142	0,131	12,997	0,519	2,200	2,779E-02
CPIJ000389	Ubiquitin conjugating enzyme 7 interacting protein		1,142	0,182	13,404	0,574	1,992	4,640E-02
CPIJ001773	Synaptic vesicle protein		1,143	0,096	21,176	0,482	2,371	1,772E-02
CPIJ019517	lactosylceramide 4-alpha-galactosyltransferase, putative		1,144	0,000	113,219	0,265	4,322	1,543E-05
CPIJ001049	protease m1 zinc metalloprotease		1,145	0,000	3273,545	0,261	4,382	1,178E-05
CPIJ006282	serine/threonine protein kinase, putative		1,146	0,030	86,374	0,391	2,929	3,402E-03
CPIJ011431	Putative uncharacterized protein		1,147	0,000	18446,043	0,132	8,719	2,805E-18
CPIJ001758	CYP4H38: cytochrome P450 4H38		1,148	0,001	347,150	0,289	3,973	7,111E-05
CPIJ007054	alpha-tocopherol transfer protein		1,148	0,017	137,514	0,365	3,150	1,635E-03
CPIJ012872	Putative uncharacterized protein	RING-type domain-containing prote	1,149	0,100	14,685	0,490	2,343	1,915E-02
CPIJ015817	Putative uncharacterized protein		1,151	0,130	13,247	0,522	2,206	2,741E-02
CPIJ004224	Pancreatic triacylglycerol lipase		1,151	0,009	164,087	0,339	3,393	6,917E-04
CPIJ006517	Putative uncharacterized protein		1,153	0,679	44,505	1,485	0,777	4,374E-01

CPIJ015223	CYP6F4: cytochrome P450 6F4		1,153	0,002	60,377	0,302	3,822	1,325E-04
CPIJ005799	Putative uncharacterized protein	C2 tensin-type domain-containing pr	1,156	0,154	14,796	0,550	2,103	3,546E-02
CPIJ006543	urokinase-type plasminogen activator precursor		1,160	0,241	7,297	0,648	1,790	7,349E-02
CPIJ007573	ADP-ribosylation factor, arf		1,161	0,176	8,813	0,577	2,012	4,425E-02
CPIJ003198	Neuropathy target esterase/swiss cheese		1,161	0,007	42,631	0,335	3,465	5,312E-04
CPIJ016993	Xaa-pro dipeptidase		1,161	0,019	31,466	0,374	3,109	1,875E-03
CPIJ012498	Putative uncharacterized protein	HMG box domain-containing protei	1,162	0,196	7,495	0,600	1,938	5,266E-02
CPIJ006370	Tak1		1,164	0,025	31,253	0,387	3,004	2,660E-03
CPIJ008959	Ribulokinase 2		1,164	0,035	24,987	0,406	2,871	4,086E-03
CPIJ000510	Putative uncharacterized protein		1,168	0,193	8,378	0,600	1,947	5,148E-02
CPIJ015577	Lola		1,168	0,132	13,052	0,532	2,196	2,811E-02
CPIJ002128	mast cell protease 2 precursor		1,169	0,000	826,999	0,238	4,909	9,153E-07
CPIJ001569	Putative uncharacterized protein	Titin	1,174	0,000	425,891	0,225	5,208	1,910E-07
CPIJ003269	broad complex isoform Z4		1,174	0,085	14,503	0,483	2,434	1,495E-02
CPIJ001195	Glyoxylate reductase/hydroxypyruvate reductase		1,176	0,007	52,088	0,338	3,479	5,032E-04
CPIJ018991	serine-type enodpeptidase, putative		1,178	0,243	6,102	0,660	1,783	7,457E-02
CPIJ003349	abc transporter, putative		1,178	0,191	7,701	0,601	1,959	5,008E-02
CPIJ009637	sterol desaturase, putative		1,179	0,000	410,157	0,223	5,285	1,257E-07
CPIJ011273	39S ribosomal protein 54, mitochondrial precursor		1,179	0,036	23,125	0,414	2,850	4,368E-03
CPIJ019523	Putative uncharacterized protein		1,181	0,005	79,839	0,330	3,579	3,452E-04
CPIJ009744	Putative uncharacterized protein	Peroxisomal membrane protein 11C	1,182	0,002	58,868	0,303	3,906	9,392E-05
CPIJ018126	Putative uncharacterized protein	Ninjurin-2	1,187	0,022	30,594	0,387	3,065	2,177E-03
CPIJ011420	Putative uncharacterized protein		1,188	0,202	9,475	0,621	1,913	5,579E-02
CPIJ016340	esterase 6 precursor		1,191	0,104	13,081	0,513	2,322	2,021E-02
CPIJ008870	protein phosphatase type 2c		1,192	0,132	10,754	0,543	2,196	2,807E-02
CPIJ001030	Zinc finger protein 235		1,195	0,130	11,634	0,543	2,202	2,763E-02
CPIJ001755	CYP4J19: cytochrome P450 4J19		1,195	0,156	10,296	0,571	2,094	3,628E-02
CPIJ006761	Glucose-6-phosphate 1-dehydrogenase		1,203	0,001	58,483	0,303	3,971	7,170E-05
CPIJ004223	pancreatic triacylglycerol lipase precursor		1,204	0,000	613,122	0,282	4,274	1,916E-05
CPIJ002070	Putative uncharacterized protein		1,207	0,000	5144,590	0,194	6,208	5,359E-10
CPIJ017178	Myoinositol oxygenase		1,211	0,237	851,557	0,673	1,798	7,210E-02
CPIJ001050	protease m1 zinc metalloprotease		1,212	0,000	829,316	0,244	4,967	6,791E-07
CPIJ014792	Putative uncharacterized protein	Myrosinase	1,213	0,166	8,637	0,591	2,051	4,027E-02
CPIJ006739	dynein light chain, putative		1,215	0,074	14,435	0,486	2,500	1,243E-02
CPIJ015126	Putative uncharacterized protein		1,217	0,196	8,011	0,628	1,938	5,265E-02
CPIJ018193	Putative uncharacterized protein	DAZ-associated protein 2	1,217	0,062	39,237	0,470	2,590	9,599E-03
CPIJ008702	Putative uncharacterized protein	Gamma-interferon inducible lysosor	1,220	0,026	26,974	0,407	2,996	2,739E-03
CPIJ006498	Goliath E3 ubiquitin ligase		1,225	0,015	30,155	0,381	3,217	1,297E-03

CPIJ012511	Uncharacterized protein (Fragment)		1,225	0,002	72,640	0,312	3,926	8,627E-05
CPIJ004547	Lariat debranching enzyme		1,226	0,111	11,136	0,536	2,288	2,213E-02
CPIJ005692	Putative uncharacterized protein		1,226	0,037	24,823	0,431	2,842	4,478E-03
CPIJ000498	Putative uncharacterized protein		1,226	0,119	11,859	0,544	2,253	2,429E-02
CPIJ001239	cathepsin B precursor		1,227	0,075	19,015	0,491	2,499	1,245E-02
CPIJ009474	CYP4D40: cytochrome P450 4D40		1,227	0,000	512,041	0,266	4,618	3,870E-06
CPIJ010480	CYP4J20: cytochrome P450 4J20		1,227	0,007	46,283	0,352	3,489	4,848E-04
CPIJ019460	Putative uncharacterized protein	Homeobox domain-containing prote	1,236	0,201	7,576	0,644	1,919	5,502E-02
CPIJ009367	Putative uncharacterized protein		1,238	0,003	60,585	0,329	3,761	1,695E-04
CPIJ004795	mitochondrial carrier protein, putative		1,238	0,023	25,391	0,407	3,041	2,357E-03
CPIJ015211	imaginal discs arrested, putative		1,238	0,187	7,702	0,628	1,973	4,855E-02
CPIJ007178	Putative uncharacterized protein	Peptidase C45 hydrolase domain-co	1,241	0,022	39,729	0,406	3,058	2,227E-03
CPIJ003317	Putative uncharacterized protein		1,241	0,000	103,080	0,274	4,530	5,887E-06
CPIJ008545	Zinc finger protein 700		1,242	0,197	7,934	0,642	1,935	5,299E-02
CPIJ007760	Putative uncharacterized protein		1,243	0,065	27,222	0,484	2,567	1,026E-02
CPIJ003966	Putative uncharacterized protein	Chitin-binding type-2 domain-conta	1,245	0,000	362,630	0,272	4,586	4,529E-06
CPIJ018720	Protein kintoun		1,246	0,004	254,731	0,343	3,629	2,850E-04
CPIJ003965	Putative uncharacterized protein	Chitin-binding type-2 domain-conta	1,251	0,000	78,595	0,279	4,491	7,087E-06
CPIJ019593	Putative uncharacterized protein	CCHC-type domain-containing prot	1,253	0,005	34,830	0,349	3,586	3,361E-04
CPIJ016093	Putative uncharacterized protein	Osteopetrosis-associated transmemb	1,254	0,007	36,504	0,361	3,470	5,197E-04
CPIJ010024	Putative uncharacterized protein	F-box domain-containing protein	1,254	0,075	18,834	0,503	2,492	1,269E-02
CPIJ017789	Serine protease		1,257	0,177	8,646	0,626	2,008	4,467E-02
CPIJ012752	cysteine synthase		1,261	0,022	54,338	0,413	3,056	2,243E-03
CPIJ009131	Serpin B4		1,262	0,040	23,505	0,449	2,807	4,999E-03
CPIJ004944	Origin recognition complex subunit 4		1,263	0,180	6,785	0,633	1,997	4,583E-02
CPIJ004115	DNA repair protein RAD50		1,263	0,075	13,146	0,506	2,497	1,254E-02
CPIJ011040	Proton-coupled amino acid transporter 1		1,264	0,001	54,324	0,317	3,986	6,715E-05
CPIJ010231	CYP12F9: cytochrome P450 12F9		1,265	0,003	74,611	0,339	3,731	1,909E-04
CPIJ008107	Putative uncharacterized protein	Nose resistant-to-fluoxetine protein	1,266	0,001	88,611	0,309	4,095	4,231E-05
CPIJ012364	Abc transporter		1,266	0,033	20,048	0,437	2,895	3,790E-03
CPIJ003953	Putative uncharacterized protein	Chitin-binding type-2 domain-conta	1,268	0,813	26,853	2,668	0,475	6,346E-01
CPIJ005898	Putative uncharacterized protein	Chitin-binding type-2 domain-conta	1,270	0,000	98,773	0,279	4,553	5,283E-06
CPIJ000425	Short-chain-fatty-acid-CoA ligase		1,270	0,104	13,750	0,547	2,320	2,037E-02
CPIJ002822	Predicted protein		1,275	0,166	7,919	0,620	2,055	3,990E-02
CPIJ014162	BTB/POZ domain containing protein		1,276	0,119	12,158	0,567	2,250	2,446E-02
CPIJ007685	PDZ domain-containing protein BBG-LP10		1,278	0,013	27,033	0,391	3,267	1,086E-03
CPIJ006569	Glutaminase		1,280	0,018	26,154	0,407	3,148	1,645E-03
CPIJ014226	Cellular retinaldehyde-binding protein		1,281	0,000	710,404	0,215	5,950	2,680E-09

CPIJ008305	Putative uncharacterized protein	C2H2-type domain-containing prote	1,283	0,159	7,068	0,617	2,081	3,739E-02
CPIJ004779	Reticulon/nogo		1,283	0,005	37,653	0,355	3,617	2,975E-04
CPIJ005632	Ganglioside induced differentiation associated protein		1,284	0,002	50,379	0,335	3,829	1,285E-04
CPIJ001759	CYP4H40: cytochrome P450 4H40		1,285	0,000	553,988	0,261	4,930	8,203E-07
CPIJ010972	Zinc finger protein		1,286	0,012	58,718	0,389	3,303	9,563E-04
CPIJ006041	Angiopoietin-2		1,287	0,031	18,470	0,442	2,915	3,561E-03
CPIJ009758	serine/threonine protein kinase, putative		1,291	0,000	99,034	0,282	4,574	4,786E-06
CPIJ003373	Predicted protein		1,292	0,001	86,606	0,322	4,012	6,023E-05
CPIJ000348	pregnancy zone protein precursor		1,294	0,226	163,255	0,706	1,833	6,674E-02
CPIJ007313	Myotubularin		1,295	0,002	57,408	0,340	3,815	1,362E-04
CPIJ002959	sterol O-acyltransferase 2		1,299	0,055	25,738	0,490	2,653	7,988E-03
CPIJ007652	synaptic vesicle protein, putative		1,300	0,041	27,521	0,465	2,796	5,168E-03
CPIJ017192	Putative uncharacterized protein		1,302	0,001	68,152	0,325	4,003	6,259E-05
CPIJ010052	Ankyrin repeat domain-containing protein 44		1,304	0,011	42,364	0,391	3,339	8,396E-04
CPIJ012036	aminopeptidase N precursor		1,306	0,000	501,398	0,263	4,963	6,939E-07
CPIJ012204	alpha-glucosidase precursor		1,307	0,000	147,717	0,253	5,158	2,499E-07
CPIJ013377	Sorbitol dehydrogenase		1,308	0,200	419,289	0,680	1,923	5,448E-02
CPIJ000192	Putative uncharacterized protein		1,311	0,091	11,861	0,547	2,398	1,650E-02
CPIJ003342	Ceramide kinase		1,311	0,000	207,265	0,239	5,484	4,158E-08
CPIJ000426	Acetyl-coenzyme A synthetase		1,314	0,000	76,036	0,302	4,352	1,349E-05
CPIJ000906	tetraspanin, putative		1,317	0,000	258,576	0,286	4,602	4,177E-06
CPIJ014208	Monocarboxylate transporter		1,319	0,000	131,534	0,239	5,529	3,225E-08
CPIJ003376	CYP6BY4: cytochrome P450 6BY4		1,321	0,024	38,303	0,437	3,024	2,496E-03
CPIJ003259	Tubulin beta-2C chain		1,323	0,018	21,527	0,422	3,139	1,694E-03
CPIJ008531	lactase-phlorizin hydrolase precursor		1,328	0,000	1379,893	0,249	5,341	9,227E-08
CPIJ007825	para-nitrobenzyl esterase		1,328	0,107	21,366	0,576	2,304	2,121E-02
CPIJ000488	Putative uncharacterized protein		1,330	0,000	12055,173	0,133	9,963	2,204E-23
CPIJ006349	Scaffold protein salvador		1,333	0,098	10,590	0,565	2,359	1,834E-02
CPIJ005848	Putative uncharacterized protein	PD-(D/E)XK endonuclease-like don	1,335	0,104	9,048	0,576	2,320	2,036E-02
CPIJ008308	Putative uncharacterized protein	Aprataxin and PNK-like factor PBZ	1,338	0,003	34,614	0,357	3,743	1,816E-04
CPIJ001041	chitooligosaccharidolytic beta-N-acetylglucosaminidase precursor		1,338	0,000	69,291	0,302	4,426	9,587E-06
CPIJ002823	Putative uncharacterized protein	C2H2-type domain-containing prote	1,340	0,097	9,233	0,567	2,363	1,811E-02
CPIJ000164	Putative uncharacterized protein		1,340	0,198	5,317	0,693	1,933	5,320E-02
CPIJ012329	Zinc finger and SCAN domain-containing protein 5		1,342	0,138	7,896	0,619	2,169	3,007E-02
CPIJ004324	Gram-negative bacteria binding protein		1,342	0,000	627,717	0,268	5,011	5,405E-07
CPIJ014224	Putative uncharacterized protein	CRAL-TRIO domain-containing prc	1,343	0,010	96,415	0,399	3,369	7,558E-04
CPIJ008379	Putative uncharacterized protein	Metallothionein	1,345	0,001	541,353	0,330	4,072	4,660E-05
CPIJ008557	Putative uncharacterized protein	Chitin-binding type-2 domain-conta	1,351	0,000	95,376	0,288	4,694	2,677E-06

CPIJ018314	SALAPY1: salivary apyrase		1,351	0,000	274,738	0,259	5,216	1,830E-07
CPIJ012933	MIT domain-containing protein 1		1,352	0,101	8,896	0,578	2,338	1,938E-02
CPIJ012596	Zinc finger protein 28		1,355	0,084	10,317	0,556	2,438	1,475E-02
CPIJ003186	Gamma-glutamyl transpeptidase		1,357	0,062	12,577	0,524	2,590	9,602E-03
CPIJ006248	D-arabinose 1-dehydrogenase		1,360	0,005	41,366	0,377	3,604	3,134E-04
CPIJ006409	Alanine-glyoxylate aminotransferase		1,361	0,006	111,307	0,384	3,541	3,980E-04
CPIJ000611	Sulfate transporter 1.2		1,368	0,039	15,998	0,485	2,819	4,821E-03
CPIJ000032	Glucosyl/glucuronosyl transferase		1,374	0,000	74,715	0,322	4,267	1,984E-05
CPIJ016520	Suppressor of defective silencing		1,375	0,098	8,422	0,583	2,357	1,844E-02
CPIJ006939	Putative uncharacterized protein		1,375	0,014	24,732	0,425	3,236	1,214E-03
CPIJ015110	Synaptic vesicle protein		1,375	0,118	10,886	0,609	2,257	2,398E-02
CPIJ005161	DNA ligase 4		1,377	0,080	10,020	0,560	2,460	1,389E-02
CPIJ018313	protein 5NUC precursor		1,378	0,000	112,204	0,304	4,528	5,941E-06
CPIJ006731	numb-associated kinase, putative		1,383	0,042	15,844	0,497	2,781	5,421E-03
CPIJ008944	Sugar transporter		1,386	0,019	31,496	0,443	3,125	1,777E-03
CPIJ009746	Putative uncharacterized protein	CRAL-TRIO domain-containing prc	1,388	0,045	12,681	0,506	2,743	6,085E-03
CPIJ020294	Putative uncharacterized protein		1,389	0,015	20,376	0,433	3,208	1,337E-03
CPIJ019182	Ribonuclease UK114		1,392	0,037	18,919	0,491	2,835	4,582E-03
CPIJ011382	chymotrypsin 1 precursor		1,396	0,008	31,109	0,408	3,423	6,198E-04
CPIJ013275	PsqA		1,399	0,121	10,110	0,624	2,240	2,507E-02
CPIJ003876	Putative uncharacterized protein	Cyclin-dependent kinase inhibitor de	1,400	0,151	6,826	0,662	2,116	3,432E-02
CPIJ005734	Putative uncharacterized protein		1,402	0,098	8,276	0,596	2,354	1,859E-02
CPIJ020221	ferritin subunit, putative		1,405	0,131	6,812	0,639	2,199	2,785E-02
CPIJ003868	Putative uncharacterized protein		1,405	0,036	21,114	0,492	2,857	4,283E-03
CPIJ008109	Putative uncharacterized protein	Nose resistant-to-fluoxetine protein	1,410	0,000	147,121	0,261	5,405	6,486E-08
CPIJ002864	Tryptophan 2,3-dioxygenase		1,411	0,015	29,875	0,440	3,205	1,349E-03
CPIJ011985	Putative uncharacterized protein	Solute carrier family 66 member 3	1,414	0,054	12,369	0,532	2,657	7,887E-03
CPIJ015482	Putative uncharacterized protein		1,414	0,183	4,971	0,712	1,986	4,700E-02
CPIJ003583	Putative uncharacterized protein	Arrestin C-terminal-like domain-cor	1,416	0,000	343,445	0,241	5,885	3,977E-09
CPIJ010004	DNA topoisomerase 2-binding protein 1		1,418	0,135	6,312	0,650	2,179	2,930E-02
CPIJ005958	CYP6AA8: cytochrome P450 6AA8		1,425	0,000	252,853	0,262	5,430	5,635E-08
CPIJ001742	Zinc carboxypeptidase		1,428	0,000	422,215	0,322	4,431	9,381E-06
CPIJ003304	Isocitrate dehydrogenase [NADP]		1,429	0,000	989,049	0,242	5,901	3,614E-09
CPIJ016224	anionic trypsin-2 precursor		1,429	0,050	14,405	0,530	2,697	7,004E-03
CPIJ007505	bumetanide-sensitive Na-K-Cl cotransport protein, putative		1,430	0,000	141,833	0,301	4,746	2,076E-06
CPIJ012074	three prime repair exonuclease 1, putative		1,432	0,105	9,066	0,618	2,315	2,059E-02
CPIJ011026	dehydrogenase/reductase SDR family member 7 precursor		1,433	0,000	55,351	0,303	4,729	2,258E-06
CPIJ001189	Putative uncharacterized protein	C3H1-type domain-containing prote	1,437	0,074	10,760	0,574	2,502	1,234E-02

CPIJ015858	Uncharacterized protein (Fragment)		1,442	0,063	9,739	0,558	2,583	9,781E-03
CPIJ003521	Putative uncharacterized protein	C2H2-type domain-containing prote	1,450	0,009	21,261	0,426	3,408	6,556E-04
CPIJ005957	CYP6AA9: cytochrome P450 6AA9		1,450	0,000	278,440	0,232	6,252	4,063E-10
CPIJ012198	Origin recognition complex subunit		1,457	0,133	6,320	0,666	2,189	2,861E-02
CPIJ002173	Putative uncharacterized protein		1,462	0,042	11,633	0,526	2,779	5,448E-03
CPIJ007911	Putative uncharacterized protein	Chitin-binding type-2 domain-conta	1,464	0,699	117,913	2,007	0,729	4,657E-01
CPIJ010068	ATP-binding cassette sub-family A member 7		1,467	0,000	473,955	0,237	6,200	5,652E-10
CPIJ003384	deoxyribonuclease I, putative		1,474	0,001	57,040	0,359	4,101	4,119E-05
CPIJ020053	glutathione transferase AtGST, putative		1,476	0,003	98,163	0,390	3,783	1,547E-04
CPIJ013017	Apoptosis stimulating of P53		1,479	0,064	9,350	0,575	2,573	1,007E-02
CPIJ010138	zinc carboxypeptidase A 1 precursor		1,479	0,000	93,700	0,344	4,303	1,686E-05
CPIJ009107	Angiotensin-converting enzyme		1,480	0,011	26,682	0,446	3,321	8,984E-04
CPIJ019291	serine protease htra2, putative		1,480	0,101	7,189	0,633	2,340	1,930E-02
CPIJ009976	Putative uncharacterized protein	Mannose-6-phosphate receptor dom	1,486	0,011	18,455	0,446	3,327	8,765E-04
CPIJ017721	gamma-glutamyltranspeptidase precursor		1,498	0,035	14,859	0,522	2,868	4,131E-03
CPIJ004238	Mitochondrial brown fat uncoupling protein		1,498	0,009	30,962	0,440	3,402	6,698E-04
CPIJ012948	Putative uncharacterized protein	DH domain-containing protein	1,500	0,000	220,234	0,274	5,465	4,620E-08
CPIJ013626	Putative uncharacterized protein	Chitin-binding type-2 domain-conta	1,503	0,655	16,900	1,800	0,835	4,038E-01
CPIJ019751	CYP6AG16: cytochrome P450 6AG16		1,504	0,074	9,067	0,601	2,504	1,228E-02
CPIJ009756	DNA topoisomerase 3-beta		1,510	0,000	139,648	0,317	4,757	1,963E-06
CPIJ003603	Putative uncharacterized protein	AN1-type domain-containing protei	1,515	0,005	21,890	0,426	3,560	3,711E-04
CPIJ013278	Putative uncharacterized protein	Farnesoic acid O-methyl transferase	1,516	0,121	430,605	0,676	2,242	2,496E-02
CPIJ002050	Homeobox protein		1,517	0,001	43,486	0,370	4,098	4,162E-05
CPIJ013304	Putative uncharacterized protein	F-box domain-containing protein	1,523	0,057	9,418	0,579	2,629	8,555E-03
CPIJ000037	UDP-glucuronosyltransferase 2B20 precursor		1,527	0,000	149,324	0,300	5,086	3,655E-07
CPIJ002744	Putative uncharacterized protein	MD-2-related lipid-recognition dom	1,527	0,003	91,343	0,412	3,710	2,073E-04
CPIJ019360	Predicted protein		1,527	0,051	10,560	0,570	2,681	7,338E-03
CPIJ010259	voltage and ligand gated potassium channel		1,530	0,001	34,119	0,365	4,189	2,803E-05
CPIJ004323	Gram-negative bacteria binding protein		1,530	0,001	74,311	0,372	4,110	3,955E-05
CPIJ012833	ficolin-1 precursor		1,532	0,101	13,070	0,655	2,340	1,928E-02
CPIJ013224	Putative uncharacterized protein	Adipose-secreted signaling protein	1,535	0,000	87,004	0,308	4,981	6,310E-07
CPIJ015854	Putative uncharacterized protein		1,554	0,024	15,999	0,513	3,033	2,421E-03
CPIJ012035	Putative uncharacterized protein		1,555	0,064	9,656	0,605	2,571	1,014E-02
CPIJ012250	Troponin C		1,558	0,011	17,149	0,469	3,323	8,897E-04
CPIJ003589	Putative uncharacterized protein	Ubiquitin-like domain-containing pr	1,568	0,000	743,697	0,256	6,129	8,845E-10
CPIJ003958	Putative uncharacterized protein	Chitin-binding type-2 domain-conta	1,568	0,000	520,104	0,304	5,163	2,424E-07
CPIJ003392	morphine 6-dehydrogenase .		1,569	0,544	8,157	1,481	1,059	2,896E-01
CPIJ018848	Mitochondrial protein import protein MAS5		1,584	0,000	62,431	0,342	4,634	3,579E-06

CPIJ018192	Putative uncharacterized protein	ADP/ATP translocase	1,592	0,000	182,995	0,237	6,704	2,029E-11
CPIJ003183	N(4)-(Beta-N-acetylglucosaminyl)-L-asparaginase precursor		1,594	0,059	8,100	0,610	2,612	8,996E-03
CPIJ001764	chorion peroxidase precursor		1,596	0,033	15,345	0,552	2,892	3,832E-03
CPIJ002236	Putative uncharacterized protein	Q rich salivary secreted protein	1,600	0,032	20,613	0,551	2,902	3,709E-03
CPIJ006721	CYP4H37: cytochrome P450 4H37		1,603	0,000	277,629	0,265	6,045	1,491E-09
CPIJ000627	Putative uncharacterized protein	C2H2-type domain-containing prote	1,623	0,049	9,937	0,598	2,713	6,677E-03
CPIJ010681	Coiled-coil domain-containing protein 22 homolog		1,624	0,005	19,917	0,454	3,578	3,457E-04
CPIJ013414	Nucleolar protein c7b		1,633	0,000	52,861	0,338	4,829	1,371E-06
CPIJ003305	NAD(P) transhydrogenase, mitochondrial precursor		1,634	0,000	1553,460	0,286	5,724	1,043E-08
CPIJ011584	Putative uncharacterized protein		1,642	0,003	22,495	0,443	3,708	2,089E-04
CPIJ016534	Putative uncharacterized protein		1,647	0,030	12,465	0,563	2,926	3,438E-03
CPIJ001938	BTG1 protein		1,651	0,043	9,415	0,598	2,761	5,758E-03
CPIJ020056	Cysteine dioxygenase		1,652	0,000	45,523	0,351	4,710	2,482E-06
CPIJ020229	CYP4D42: cytochrome P450 4D42		1,653	0,001	28,908	0,402	4,112	3,925E-05
CPIJ003787	Putative uncharacterized protein	Protein with signal anchor	1,656	0,085	95,904	0,681	2,431	1,506E-02
CPIJ012013	serine protease inhibitor, serpin		1,662	0,065	10,728	0,647	2,567	1,025E-02
CPIJ004320	gram-negative bacteria-binding protein 1 precursor		1,663	0,170	84,782	0,817	2,035	4,187E-02
CPIJ005697	n-acetylgalactosaminyltransferase		1,665	0,000	50,780	0,314	5,294	1,195E-07
CPIJ000415	Phosphatidyltransferase		1,665	0,001	32,731	0,399	4,172	3,023E-05
CPIJ000230	Putative uncharacterized protein	tRNA-splicing endonuclease subuni	1,666	0,059	7,008	0,636	2,618	8,853E-03
CPIJ006497	Putative uncharacterized protein	RING-type domain-containing prote	1,670	0,007	16,741	0,479	3,487	4,881E-04
CPIJ000007	Chitinase		1,674	0,007	23,667	0,485	3,454	5,529E-04
CPIJ008523	serine-type enodpeptidase, putative		1,674	0,009	19,279	0,494	3,388	7,033E-04
CPIJ003524	Annulin		1,678	0,049	7,705	0,620	2,706	6,809E-03
CPIJ007828	bile salt-activated lipase precursor		1,687	0,026	11,056	0,565	2,986	2,830E-03
CPIJ012677	Sugar transporter		1,687	0,076	6,109	0,678	2,487	1,288E-02
CPIJ006606	Molybdopterin cofactor synthesis protein a		1,688	0,000	160,846	0,264	6,392	1,635E-10
CPIJ013381	Sorbitol dehydrogenase		1,688	0,015	15,748	0,527	3,201	1,368E-03
CPIJ008074	glucosamine-6-phosphate isomerase		1,688	0,000	35,097	0,376	4,491	7,100E-06
CPIJ005956	CYP6BZ2: cytochrome P450 6BZ2		1,694	0,000	686,620	0,245	6,915	4,673E-12
CPIJ000147	Putative uncharacterized protein	PDZ domain-containing protein	1,696	0,043	11,480	0,613	2,769	5,627E-03
CPIJ010345	Putative uncharacterized protein		1,696	0,020	11,961	0,549	3,090	1,999E-03
CPIJ010806	Putative uncharacterized protein	Peptidase M14 carboxypeptidase A	1,700	0,010	21,278	0,507	3,351	8,039E-04
CPIJ001265	Alkaline phosphatase		1,702	0,000	523,241	0,275	6,191	5,972E-10
CPIJ000988	Sugar transporter		1,713	0,027	9,179	0,575	2,977	2,911E-03
CPIJ008283	Multidrug resistance protein 2		1,713	0,045	7,788	0,624	2,746	6,033E-03
CPIJ001560	calcium-binding protein, putative		1,718	0,283	25,353	1,031	1,666	9,565E-02
CPIJ002138	chymotrypsinogen, putative		1,722	0,000	27,018	0,402	4,277	1,891E-05

CPIJ007204	apolipophorins precursor		1,726	0,042	16,974	0,621	2,780	5,430E-03
CPIJ015813	Putative uncharacterized protein	chloroplast protein-transporting ATI	1,727	0,004	18,894	0,473	3,651	2,608E-04
CPIJ013511	Putative uncharacterized protein		1,729	0,043	8,930	0,626	2,762	5,742E-03
CPIJ007910	Putative uncharacterized protein	Chitin-binding type-2 domain-conta	1,733	0,608	42,458	1,874	0,925	3,551E-01
CPIJ015670	4-coumarate-CoA ligase 3		1,733	0,012	15,723	0,528	3,285	1,020E-03
CPIJ002818	Putative uncharacterized protein	Chitin-binding type-2 domain-conta	1,741	0,473	15,628	1,435	1,213	2,252E-01
CPIJ011298	Brachyurin		1,743	0,003	28,897	0,462	3,774	1,605E-04
CPIJ002025	Putative uncharacterized protein	FYVE-type domain-containing prote	1,752	0,000	33,961	0,366	4,789	1,674E-06
CPIJ016854	CYP6N22: cytochrome P450 6N22		1,761	0,000	34,627	0,390	4,514	6,363E-06
CPIJ017122	Putative uncharacterized protein	Superoxide dismutase copper/zinc b	1,763	0,015	14,169	0,549	3,211	1,320E-03
CPIJ016551	Caspase-1		1,770	0,000	33,876	0,365	4,851	1,227E-06
CPIJ013879	DNA polymerase theta		1,771	0,003	18,113	0,470	3,771	1,625E-04
CPIJ002628	sensory appendage protein, putative		1,781	0,050	8,833	0,661	2,694	7,060E-03
CPIJ017014	CYP6AG14: cytochrome P450 6AG14		1,785	0,000	54,280	0,315	5,673	1,406E-08
CPIJ019446	copper transport protein		1,789	0,011	15,339	0,536	3,338	8,451E-04
CPIJ002659	Transmembrane protease		1,793	0,085	4,829	0,737	2,433	1,499E-02
CPIJ005655	Oxidoreductase		1,803	0,000	48,115	0,337	5,344	9,083E-08
CPIJ006210	FK506-binding protein 6		1,806	0,074	5,215	0,722	2,503	1,232E-02
CPIJ008530	lactase-phlorizin hydrolase precursor		1,807	0,054	123,593	0,679	2,661	7,794E-03
CPIJ016094	Putative uncharacterized protein	Osteopetrosis-associated transmemb	1,813	0,000	40,260	0,382	4,749	2,041E-06
CPIJ002867	AMP dependent coa ligase, putative		1,818	0,039	236,655	0,647	2,812	4,929E-03
CPIJ000039	glucosyl/glucuronosyl transferases		1,821	0,000	248,829	0,249	7,308	2,710E-13
CPIJ010173	Testis-specific protein pbs13		1,821	0,000	67,501	0,357	5,105	3,304E-07
CPIJ011550	tubulin alpha-2 chain		1,833	0,015	14,359	0,572	3,206	1,344E-03
CPIJ000133	Putative uncharacterized protein	LIM zinc-binding domain-containin	1,834	0,027	7,944	0,619	2,965	3,024E-03
CPIJ013510	Putative uncharacterized protein		1,839	0,413	46,387	1,358	1,354	1,756E-01
CPIJ011198	Threonine dehydratase/deaminase		1,840	0,445	67,951	1,442	1,277	2,018E-01
CPIJ000146	superoxide dismutase 2		1,866	0,062	6,652	0,721	2,587	9,679E-03
CPIJ039829	Aminopeptidase		1,869	0,043	413,244	0,676	2,763	5,728E-03
CPIJ018794	Putative uncharacterized protein	14.5 kDa salivary protein	1,887	0,007	12,409	0,546	3,455	5,496E-04
CPIJ016147	Predicted protein		1,889	0,045	6,826	0,687	2,750	5,968E-03
CPIJ013341	Putative uncharacterized protein		1,911	0,000	28,828	0,401	4,767	1,874E-06
CPIJ004556	Putative uncharacterized protein	E3 ubiquitin-protein ligase KCMF1	1,914	0,000	35,428	0,374	5,116	3,113E-07
CPIJ016736	cysteine-rich venom protein, putative		1,920	0,020	14,983	0,620	3,099	1,943E-03
CPIJ018355	smad, putative		1,929	0,018	8,947	0,615	3,137	1,710E-03
CPIJ006075	trypsin beta precursor		1,930	0,000	114,758	0,351	5,491	3,991E-08
CPIJ001859	Sodium/solute symporter		1,932	0,009	11,112	0,567	3,407	6,558E-04
CPIJ009407	Putative uncharacterized protein	Chitin-binding type-2 domain-conta	1,932	0,310	85,923	1,211	1,596	1,106E-01

CPIJ006267	LRIM16: leucine-rich immune protein (TM)		1,949	0,000	37,562	0,366	5,320	1,035E-07
CPIJ002680	Glutathione S-transferase		1,952	0,005	12,835	0,546	3,576	3,491E-04
CPIJ020318	Putative uncharacterized protein	BZIP domain-containing protein	1,968	0,027	9,201	0,661	2,978	2,900E-03
CPIJ003372	Putative uncharacterized protein		1,978	0,050	5,471	0,734	2,696	7,011E-03
CPIJ015654	lysosomal alpha-mannosidase		1,978	0,000	54,024	0,408	4,844	1,273E-06
CPIJ010548	CYP9J39: cytochrome P450 9J39		1,991	0,002	18,676	0,521	3,820	1,332E-04
CPIJ018465	Putative uncharacterized protein	Chitin-binding type-2 domain-conta	1,993	0,610	70,653	2,163	0,921	3,569E-01
CPIJ005332	CYP9J43: cytochrome P450 9J43		1,994	0,085	45,016	0,819	2,434	1,492E-02
CPIJ011267	Putative uncharacterized protein		2,002	0,042	5,705	0,721	2,775	5,517E-03
CPIJ011185	deoxyribonuclease I, putative		2,016	0,000	112,239	0,315	6,400	1,558E-10
CPIJ017156	Ankyrin repeat and SOCS box protein 13		2,018	0,000	20,093	0,451	4,469	7,844E-06
CPIJ011427	Putative uncharacterized protein	U2A'/phosphoprotein 32 family A C	2,022	0,003	12,982	0,545	3,709	2,083E-04
CPIJ008946	Sugar transporter		2,057	0,017	8,624	0,651	3,162	1,569E-03
CPIJ002235	Putative uncharacterized protein		2,059	0,337	41,451	1,347	1,529	1,263E-01
CPIJ004718	Putative uncharacterized protein	C2H2-type domain-containing prote	2,059	0,024	6,473	0,679	3,034	2,412E-03
CPIJ004411	CYP6Z12: cytochrome P450 6Z12		2,065	0,000	260,634	0,305	6,766	1,323E-11
CPIJ009347	Zinc finger protein 394		2,073	0,062	4,427	0,801	2,588	9,642E-03
CPIJ003315	Putative uncharacterized protein	THAP-type domain-containing prot	2,073	0,062	4,427	0,801	2,588	9,642E-03
CPIJ004570	Putative uncharacterized protein	Oxidative stress-responsive serine-ri	2,083	0,023	6,197	0,686	3,038	2,383E-03
CPIJ007903	peritrophin-1 precursor		2,094	0,478	29,101	1,740	1,203	2,289E-01
CPIJ005492	Putative uncharacterized protein	Sushi domain-containing protein	2,102	0,000	139,574	0,260	8,101	5,460E-16
CPIJ001757	CYP4H39: cytochrome P450 4H39		2,108	0,000	123,671	0,266	7,911	2,548E-15
CPIJ005777	synaptic vesicle protein, putative		2,108	0,201	15,756	1,098	1,920	5,492E-02
CPIJ011346	Notch		2,113	0,000	16,674	0,494	4,278	1,889E-05
CPIJ010541	CYP9J37: cytochrome P450 9J37		2,120	0,000	535,261	0,275	7,716	1,202E-14
CPIJ012944	fad nad binding oxidoreductases		2,150	0,026	6,812	0,718	2,996	2,734E-03
CPIJ008555	Uncharacterized protein (Fragment)		2,160	0,009	9,398	0,638	3,385	7,109E-04
CPIJ007899	Putative uncharacterized protein	Chitin-binding type-2 domain-conta	2,182	0,550	55,516	2,094	1,042	2,973E-01
CPIJ004230	Lipase		2,202	0,000	139,561	0,345	6,376	1,816E-10
CPIJ015980	Putative uncharacterized protein	Chitin-binding type-2 domain-conta	2,210	0,109	288,371	0,964	2,294	2,179E-02
CPIJ008498	Putative uncharacterized protein	Chitin-binding type-2 domain-conta	2,212	0,479	35,017	1,842	1,201	2,298E-01
CPIJ011772	Homeobox protein		2,235	0,004	11,002	0,616	3,630	2,833E-04
CPIJ012045	ficolin-2 precursor		2,239	0,002	11,796	0,576	3,886	1,020E-04
CPIJ013933	Putative uncharacterized protein		2,242	0,000	20,339	0,491	4,567	4,949E-06
CPIJ018121	membrane-bound alkaline phosphatase precursor		2,248	0,055	155,553	0,847	2,655	7,942E-03
CPIJ014140	Putative uncharacterized protein	DEUBAD domain-containing protei	2,250	0,031	4,998	0,771	2,919	3,510E-03
CPIJ009106	angiotensin-converting enzyme precursor		2,261	0,001	12,079	0,569	3,974	7,078E-05
CPIJ007082	TIM: timeless circadian protein		2,273	0,000	24,951	0,504	4,508	6,558E-06

CPIJ012055	sodium/chloride dependent amino acid transporter, putative		2,280	0,002	11,184	0,585	3,894	9,857E-05
CPIJ003274	vacuolar proton translocating ATPase 116 kDa subunit a isoform 1		2,291	0,058	102,318	0,875	2,620	8,804E-03
CPIJ010230	CYP12F10: cytochrome P450 12F10		2,319	0,000	26,553	0,430	5,394	6,896E-08
CPIJ013273	Putative uncharacterized protein	Zinc finger DNA binding protein	2,328	0,026	4,849	0,779	2,990	2,793E-03
CPIJ002073	juvenile hormone esterase		2,332	0,000	25,906	0,447	5,224	1,752E-07
CPIJ007906	Putative uncharacterized protein	Chitin-binding type-2 domain-conta	2,342	0,584	16,139	2,402	0,975	3,297E-01
CPIJ007902	Putative uncharacterized protein	Chitin-binding type-2 domain-conta	2,344	0,573	112,670	2,360	0,993	3,205E-01
CPIJ012831	Fibrinogen and fibronectin		2,345	0,000	41,356	0,426	5,508	3,624E-08
CPIJ016104	kallikrein-6 precursor		2,355	0,000	21,211	0,542	4,342	1,409E-05
CPIJ007897	Putative uncharacterized protein	Chitin-binding type-2 domain-conta	2,356	0,479	64,607	1,961	1,202	2,295E-01
CPIJ001747	tep3		2,389	0,000	28,024	0,439	5,447	5,137E-08
CPIJ010067	ATP-binding cassette sub-family A member 3		2,402	0,000	269,934	0,343	7,012	2,343E-12
CPIJ005728	Putative uncharacterized protein	Ig-like domain-containing protein	2,406	0,000	22,553	0,442	5,443	5,233E-08
CPIJ008499	Putative uncharacterized protein	Chitin-binding type-2 domain-conta	2,425	0,098	24,890	1,030	2,354	1,859E-02
CPIJ013793	Cuticle protein 8		2,431	0,000	106,634	0,360	6,761	1,374E-11
CPIJ010313	Putative uncharacterized protein	CHK kinase-like domain-containing	2,443	0,005	7,820	0,676	3,612	3,033E-04
CPIJ016301	Serine protease inhibitor A3G		2,448	0,000	12,682	0,567	4,314	1,601E-05
CPIJ002849	Putative uncharacterized protein	Single domain-containing protein	2,482	0,000	21,258	0,455	5,460	4,748E-08
CPIJ006081	trypsin alpha precursor		2,483	0,000	24,652	0,551	4,510	6,486E-06
CPIJ017285	Putative uncharacterized protein	F-box domain-containing protein	2,484	0,006	7,087	0,702	3,541	3,990E-04
CPIJ016105	trypsin-3 precursor		2,487	0,002	17,159	0,644	3,863	1,120E-04
CPIJ000365	Putative uncharacterized protein		2,491	0,005	7,151	0,699	3,565	3,634E-04
CPIJ010307	Putative uncharacterized protein	CHK kinase-like domain-containing	2,543	0,000	310,310	0,219	11,597	4,271E-31
CPIJ019874	salivary cysteine-rich peptide		2,546	0,000	15,227	0,531	4,796	1,616E-06
CPIJ002937	trypsin delta/gamma precursor		2,555	0,182	92,573	1,283	1,992	4,640E-02
CPIJ008079	alpha-amylase 1 precursor		2,568	0,000	1269,734	0,224	11,447	2,443E-30
CPIJ002863	Putative uncharacterized protein		2,596	0,004	7,139	0,711	3,650	2,618E-04
CPIJ000495	Putative uncharacterized protein		2,601	0,001	210,325	0,638	4,075	4,600E-05
CPIJ010456	Cysteine dioxygenase		2,612	0,000	48,302	0,379	6,884	5,829E-12
CPIJ004228	Lipase		2,668	0,000	972,325	0,280	9,535	1,501E-21
CPIJ014406	Putative uncharacterized protein		2,684	0,000	21,685	0,516	5,201	1,986E-07
CPIJ001777	ataxia telangiectasia mutated, putative		2,686	0,001	9,725	0,650	4,135	3,542E-05
CPIJ007777	Putative uncharacterized protein		2,702	0,000	11,200	0,617	4,382	1,177E-05
CPIJ003983	Putative uncharacterized protein		2,723	0,049	39,585	1,007	2,706	6,817E-03
CPIJ007898	Putative uncharacterized protein	Chitin-binding type-2 domain-conta	2,727	0,510	103,290	2,403	1,135	2,564E-01
CPIJ002066	alpha-galactosidase A precursor		2,757	0,000	105,955	0,338	8,163	3,270E-16
CPIJ010308	Putative uncharacterized protein	CHK kinase-like domain-containing	2,764	0,000	35,443	0,393	7,040	1,928E-12
CPIJ000500	Putative uncharacterized protein		2,766	0,000	254,107	0,360	7,684	1,546E-14

CPIJ006619	Cystathionine gamma-lyase		2,779	0,091	68,303	1,157	2,401	1,634E-02
CPIJ017792	Serine protease		2,790	0,005	5,673	0,782	3,569	3,580E-04
CPIJ004730	Putative uncharacterized protein	Chitin-binding type-2 domain-conta	2,793	0,000	1464,315	0,296	9,430	4,097E-21
CPIJ015809	Putative uncharacterized protein	Transmembrane protein 267	2,861	0,000	9,343	0,661	4,326	1,517E-05
CPIJ010412	Fucosyltransferase 11		2,878	0,000	9,895	0,673	4,276	1,906E-05
CPIJ008490	Ankyrin repeat domain-containing protein 44		2,940	0,000	42,565	0,404	7,275	3,454E-13
CPIJ008127	Putative uncharacterized protein		2,944	0,002	7,088	0,750	3,927	8,614E-05
CPIJ012159	Sphingomyelin phosphodiesterase		3,000	0,049	38,610	1,106	2,713	6,667E-03
CPIJ008501	Putative uncharacterized protein	Chitin-binding type-2 domain-conta	3,019	0,228	27,592	1,653	1,827	6,775E-02
CPIJ002535	CYP6AG9: cytochrome P450 6AG9		3,032	0,000	56,663	0,401	7,569	3,764E-14
CPIJ001812	Sugar transporter		3,068	0,000	13,136	0,662	4,631	3,636E-06
CPIJ001996	Putative uncharacterized protein		3,109	0,000	9,123	0,702	4,429	9,449E-06
CPIJ010546	CYP9J34: cytochrome P450 9J34		3,109	0,000	1023,738	0,312	9,952	2,461E-23
CPIJ017065	NMDA-type glutamate receptor subunit 1		3,149	0,000	20,962	0,528	5,966	2,433E-09
CPIJ014178	Putative uncharacterized protein	Chitin-binding type-2 domain-conta	3,151	0,160	33,246	1,518	2,076	3,787E-02
CPIJ016697	Leukocyte elastase inhibitor		3,215	0,000	7,636	0,750	4,287	1,812E-05
CPIJ000494	Putative uncharacterized protein		3,215	0,000	458,944	0,622	5,166	2,392E-07
CPIJ010175	CYP9J48: cytochrome P450 9J48		3,220	0,000	260,587	0,356	9,048	1,452E-19
CPIJ011190	deoxyribonuclease I, putative		3,260	0,000	227,742	0,272	11,994	3,827E-33
CPIJ040816	Putative uncharacterized protein		3,283	0,072	12,206	1,306	2,513	1,196E-02
CPIJ008502	Putative uncharacterized protein	Chitin-binding type-2 domain-conta	3,320	0,066	13,396	1,300	2,553	1,067E-02
CPIJ000699	Vago: salivary Cys-rich secreted protein Vago		3,434	0,003	4,910	0,921	3,730	1,917E-04
CPIJ000496	Putative uncharacterized protein	Secreted protein	3,441	0,000	822,812	0,736	4,675	2,937E-06
CPIJ005229	N-acetylgalactosaminyltransferase 6		3,482	0,002	5,460	0,899	3,874	1,070E-04
CPIJ005640	Heat shock protein 26		3,664	0,000	9,051	0,799	4,587	4,492E-06
CPIJ003982	Putative uncharacterized protein		3,960	0,001	27,775	0,938	4,220	2,446E-05
CPIJ004489	potassium-dependent sodium-calcium exchanger, putative		4,187	0,000	50,418	0,858	4,881	1,054E-06
CPIJ003458	Putative uncharacterized protein	N-acetyltransferase domain-containi	4,259	0,000	8,208	0,894	4,766	1,878E-06
CPIJ000094	Putative uncharacterized protein		4,937	0,000	13,785	0,837	5,900	3,635E-09
CPIJ006306	Ion channel nompc		5,122	0,000	50,122	0,536	9,562	1,155E-21
CPIJ000093	Putative uncharacterized protein		5,190	0,000	8,320	1,109	4,679	2,881E-06
CPIJ016362	alpha-glucosidase precursor		5,912	0,000	3,871	1,309	4,517	6,268E-06
CPIJ010801	carboxypeptidase B precursor		6,001	0,000	137,607	0,433	13,848	1,301E-43
CPIJ013861	Putative uncharacterized protein		6,849	0,000	14,585	1,224	5,597	2,180E-08
CPIJ040083	COX3: cytochrome c oxidase subunit III		7,017	0,000	8,260	1,286	5,457	4,842E-08
CPIJ000344	39S ribosomal protein L24, mitochondrial precursor		7,373	0,000	10,526	1,281	5,754	8,708E-09
CPIJ002522	Farnesol dehydrogenase		7,628	0,000	12,597	1,264	6,033	1,605E-09
CPIJ009033	arylphorin subunit C223 precursor		9,484	0,000	45,994	1,956	4,850	1,235E-06

Table S4. Differentially expressed genes from the *Culex quinquefasciatus* Bin-resistant strains (REC and REC-2), compared to a susceptible strain (S).
(B) REC-2 X S up and down regulated genes.

ID	Description	Uniprot Annotation	log2Fold Change	padj	baseMean	lfcSE	stat	pvalue
CPIJ017592	Vanin-like protein 1 precursor, putative		-8,265	0,000	43,044	1,212	-6,821	9,064E-12
CPIJ017593	pantetheinase precursor		-8,173	0,000	78,454	1,164	-7,024	2,163E-12
CPIJ005154	Nuclear transport factor 2		-6,705	0,000	14,678	1,236	-5,424	5,817E-08
CPIJ012840	39S ribosomal protein L24, mitochondrial precursor		-6,604	0,000	13,612	1,233	-5,354	8,591E-08
CPIJ014889	Pyruvate dehydrogenase E1 component subunit alpha		-6,453	0,000	12,320	1,239	-5,208	1,906E-07
CPIJ002679	Glutathione S-transferase theta-2		-5,731	0,002	123,580	1,477	-3,880	1,045E-04
CPIJ009924	Putative uncharacterized protein		-5,514	0,000	6,459	1,266	-4,356	1,327E-05
CPIJ017634	Disulfide oxidoreductase		-5,247	0,001	5,254	1,280	-4,100	4,125E-05
CPIJ017291	Putative uncharacterized protein	DNA damage-binding protein 1	-4,866	0,003	4,070	1,286	-3,782	1,553E-04
CPIJ013539	ankyrin repeat and SOCS box protein 10		-4,792	0,004	3,917	1,289	-3,718	2,011E-04
CPIJ018744	ankyrin 2,3/unc44		-4,756	0,000	40,751	0,651	-7,310	2,681E-13
CPIJ006466	H/ACA ribonucleoprotein complex subunit 4		-4,546	0,008	3,220	1,305	-3,483	4,964E-04
CPIJ002989	Oseg1		-4,386	0,006	5,974	1,223	-3,588	3,338E-04
CPIJ007230	Regucalcin		-4,349	0,007	5,607	1,230	-3,537	4,047E-04
CPIJ019172	Histone deacetylase		-4,272	0,016	2,745	1,315	-3,250	1,156E-03
CPIJ012061	hyp37.3 putativesecreted salivary gland protein		-4,164	0,000	18,563	0,814	-5,116	3,122E-07
CPIJ013173	Neutral and basic amino acid transport protein rBAT	Culex quinquefasciatus maltase 1	-4,140	0,000	1194,580	0,210	-19,754	7,346E-87
CPIJ016846	CYP6M13: cytochrome P450 6M13		-4,010	0,000	85,651	0,767	-5,231	1,690E-07
CPIJ019567	vegetatible incompatibility protein HET-E-1, putative		-4,001	0,004	8,274	1,078	-3,711	2,065E-04
CPIJ012066	sodium/chloride dependent amino acid transporter, putative		-3,914	0,000	633,940	0,273	-14,326	1,516E-46
CPIJ014435	Putative uncharacterized protein	Cuticle protein	-3,903	0,012	17,633	1,169	-3,339	8,404E-04
CPIJ012702	Putative uncharacterized protein	CHK kinase-like domain-containing protein	-3,756	0,000	17,432	0,736	-5,104	3,320E-07
CPIJ005064	alpha-amylase precursor		-3,720	0,034	3,927	1,257	-2,959	3,083E-03
CPIJ012697	Putative uncharacterized protein	CHK kinase-like domain-containing protein	-3,710	0,000	19,122	0,682	-5,439	5,366E-08
CPIJ015140	Putative uncharacterized protein	PiggyBac transposable element-derived protein dc	-3,703	0,034	3,672	1,252	-2,958	3,099E-03
CPIJ011602	NADH-cytochrome b5 reductase		-3,577	0,002	9,440	0,923	-3,876	1,062E-04
CPIJ017738	plasminogen precursor		-3,559	0,045	3,564	1,255	-2,836	4,561E-03
CPIJ010515	Phosphoenolpyruvate carboxykinase		-3,541	0,051	3,519	1,271	-2,785	5,349E-03
CPIJ004607	Putative uncharacterized protein	C-type lectin domain-containing protein	-3,256	0,039	4,842	1,121	-2,904	3,679E-03
CPIJ007273	D-amino acid oxidase		-3,226	0,089	2,661	1,273	-2,533	1,131E-02
CPIJ010699	CecA1: Cecropin A		-3,212	0,004	9,357	0,870	-3,690	2,239E-04
CPIJ018624	Glutathione-s-transferase theta, gst		-3,209	0,000	73,302	0,396	-8,103	5,382E-16
CPIJ015947	purine nucleoside phosphorylase		-3,196	0,096	2,930	1,280	-2,496	1,256E-02
CPIJ008729	4-aminobutyrate aminotransferase, mitochondrial precursor		-3,184	0,000	19,642	0,661	-4,818	1,451E-06

CPIJ012700	CHKov1, putative		-3,135	0,000	42,879	0,453	-6,920	4,522E-12
CPIJ017652	GPI transamidase component PIG-T precursor		-3,115	0,016	6,887	0,961	-3,242	1,186E-03
CPIJ017473	Organic cation transporter		-3,065	0,000	13,739	0,691	-4,432	9,316E-06
CPIJ012841	Valacyclovir hydrolase		-3,048	0,059	4,298	1,119	-2,724	6,444E-03
CPIJ007525	Putative uncharacterized protein	Gamma-interferon inducible lysosomal thiol reduc	-3,040	0,000	19,878	0,584	-5,205	1,937E-07
CPIJ002307	Putative uncharacterized protein		-3,033	0,070	3,955	1,144	-2,651	8,029E-03
CPIJ005656	Oxidoreductase		-3,006	0,000	15,158	0,656	-4,586	4,528E-06
CPIJ004537	Double-strand break repair protein MRE11		-2,965	0,070	4,189	1,120	-2,647	8,126E-03
CPIJ018326	Putative uncharacterized protein	MD-2-related lipid-recognition domain-containing	-2,915	0,000	253,745	0,317	-9,203	3,470E-20
CPIJ018626	Glutathione-s-transferase theta, gst		-2,892	0,000	236,473	0,312	-9,281	1,679E-20
CPIJ012829	Fibrinogen and fibronectin		-2,841	0,000	110,063	0,302	-9,406	5,134E-21
CPIJ007346	TTC27 protein		-2,823	0,011	7,832	0,840	-3,363	7,714E-04
CPIJ012699	Putative uncharacterized protein	CHK kinase-like domain-containing protein	-2,819	0,003	10,353	0,749	-3,765	1,662E-04
CPIJ003266	CCAAT/enhancer-binding protein		-2,808	0,002	10,474	0,727	-3,864	1,114E-04
CPIJ012562	26S proteasome non-ATPase regulatory subunit 10		-2,761	0,102	3,937	1,120	-2,465	1,369E-02
CPIJ000718	Transketolase		-2,750	0,112	3,635	1,137	-2,419	1,557E-02
CPIJ004873	Putative uncharacterized protein	RING finger and SPRY domain-containing protei	-2,727	0,002	11,820	0,687	-3,972	7,124E-05
CPIJ019428	trypsin 2 precursor		-2,693	0,023	7,226	0,870	-3,096	1,958E-03
CPIJ008739	Polycomb protein		-2,668	0,130	3,433	1,136	-2,348	1,888E-02
CPIJ002675	Glutathione S-transferase 1		-2,648	0,003	1098,694	0,700	-3,782	1,557E-04
CPIJ016453	Acyl-coa dehydrogenase		-2,616	0,000	24,913	0,494	-5,294	1,198E-07
CPIJ020105	apolipoprotein D, putative		-2,603	0,015	8,140	0,797	-3,268	1,083E-03
CPIJ002194	Synaptojanin		-2,571	0,076	4,638	0,985	-2,610	9,048E-03
CPIJ015685	3-oxoacyl-[acyl-carrier-protein] reductase		-2,542	0,167	3,099	1,153	-2,205	2,749E-02
CPIJ013313	apolipoprotein D, putative		-2,503	0,116	39,658	1,042	-2,401	1,633E-02
CPIJ016639	acetyl-coa synthetase		-2,499	0,000	72,593	0,348	-7,182	6,865E-13
CPIJ006734	DNA-directed RNA polymerase II 16 kDa polypeptide		-2,497	0,007	9,754	0,710	-3,515	4,391E-04
CPIJ000225	UDP-glucuronosyltransferase R-21		-2,482	0,000	105,729	0,288	-8,609	7,357E-18
CPIJ006014	Serine protease		-2,462	0,184	3,544	1,145	-2,150	3,152E-02
CPIJ008664	Putative uncharacterized protein	Protein TsetseEP domain-containing protein	-2,435	0,000	128,727	0,389	-6,262	3,795E-10
CPIJ012919	GDP-mannose 4,6 dehydratase		-2,421	0,117	3,971	1,010	-2,397	1,654E-02
CPIJ018769	Miranda		-2,403	0,012	9,147	0,721	-3,333	8,592E-04
CPIJ002676	Glutathione S-transferase D7		-2,379	0,000	64,479	0,339	-7,024	2,152E-12
CPIJ014327	Sugar transporter SWEET		-2,374	0,026	7,548	0,778	-3,052	2,271E-03
CPIJ019704	CYP6N24: cytochrome P450 6N24		-2,340	0,002	13,146	0,608	-3,852	1,174E-04
CPIJ012065	tryptophan transporter		-2,339	0,000	40,711	0,419	-5,587	2,305E-08
CPIJ015938	Putative uncharacterized protein		-2,328	0,070	6,178	0,878	-2,650	8,046E-03
CPIJ009775	Putative uncharacterized protein		-2,321	0,131	4,124	0,992	-2,340	1,929E-02

CPIJ004709	Bifunctional protein folD		-2,312	0,000	46,653	0,454	-5,087	3,642E-07
CPIJ018994	Uncharacterized protein (Fragment)		-2,306	0,130	4,542	0,983	-2,347	1,894E-02
CPIJ013981	mediator complex, subunit, putative		-2,292	0,236	2,715	1,157	-1,981	4,761E-02
CPIJ005349	lysosomal acid lipase, putative		-2,262	0,053	6,578	0,817	-2,767	5,661E-03
CPIJ012605	Exu-associated protein		-2,245	0,154	3,902	0,998	-2,248	2,454E-02
CPIJ005792	nuclear pore complex protein Nup93		-2,240	0,149	4,293	0,986	-2,272	2,310E-02
CPIJ005505	Putative uncharacterized protein		-2,233	0,000	20,748	0,500	-4,468	7,897E-06
CPIJ010860	cat eye syndrome critical region protein 1 precursor		-2,231	0,255	2,627	1,160	-1,924	5,441E-02
CPIJ007924	Proteasome assembly chaperone 2		-2,227	0,177	3,492	1,026	-2,171	2,995E-02
CPIJ005214	Ceramide glucosyltransferase		-2,221	0,000	30,753	0,429	-5,175	2,280E-07
CPIJ013570	protein sym-1		-2,220	0,092	5,398	0,882	-2,518	1,180E-02
CPIJ016852	CYP6N19: cytochrome P450 6N19		-2,201	0,000	227,305	0,268	-8,212	2,173E-16
CPIJ007224	Methyl-cpg binding protein		-2,194	0,102	4,866	0,890	-2,465	1,369E-02
CPIJ002336	Putative uncharacterized protein	Acetyl-coa transporter	-2,180	0,001	19,566	0,539	-4,043	5,284E-05
CPIJ009657	Putative uncharacterized protein	Nucleolar protein 16	-2,175	0,066	6,363	0,813	-2,675	7,465E-03
CPIJ010919	protein will die slowly		-2,168	0,143	4,365	0,945	-2,296	2,170E-02
CPIJ008426	Putative uncharacterized protein	Monocarboxylate transporter	-2,165	0,277	2,642	1,161	-1,864	6,230E-02
CPIJ015946	guanylate cyclase, putative		-2,161	0,292	2,317	1,185	-1,824	6,822E-02
CPIJ003163	Putative uncharacterized protein		-2,156	0,007	12,270	0,613	-3,518	4,345E-04
CPIJ011623	Putative uncharacterized protein	Chitin-binding type-2 domain-containing protein	-2,151	0,000	86,516	0,339	-6,352	2,126E-10
CPIJ002911	retinoid-inducible serine carboxypeptidase precursor		-2,151	0,000	100,166	0,272	-7,903	2,712E-15
CPIJ007010	Peroxisomal membrane protein pmp34		-2,129	0,016	11,764	0,656	-3,245	1,175E-03
CPIJ012180	Putative uncharacterized protein	MD-2-related lipid-recognition domain-containing	-2,123	0,000	1108,246	0,184	-11,535	8,794E-31
CPIJ009939	Putative uncharacterized protein		-2,110	0,050	8,172	0,756	-2,792	5,231E-03
CPIJ004068	Suppressor of T-cell receptor signaling 1		-2,109	0,000	36,021	0,453	-4,652	3,284E-06
CPIJ015475	Thiamin pyrophosphokinase 1		-2,107	0,179	4,436	0,975	-2,163	3,058E-02
CPIJ005160	serine protease htra2, putative		-2,100	0,110	5,349	0,866	-2,426	1,526E-02
CPIJ014772	techylectin-5B precursor		-2,099	0,044	9,362	0,738	-2,845	4,447E-03
CPIJ014142	nucleotide-binding protein 1		-2,091	0,012	14,774	0,623	-3,355	7,946E-04
CPIJ005304	DNA-directed RNA polymerase II subunit J		-2,088	0,074	6,229	0,797	-2,622	8,745E-03
CPIJ005595	Fatty acid synthase S-acetyltransferase		-2,086	0,000	187,945	0,279	-7,482	7,346E-14
CPIJ016341	alpha-esterase		-2,084	0,577	11,348	1,793	-1,162	2,452E-01
CPIJ005780	Pre-mRNA-splicing factor cef-1		-2,070	0,092	5,621	0,822	-2,518	1,181E-02
CPIJ000058	Receptor protein tyrosine kinase		-2,069	0,118	5,267	0,865	-2,392	1,675E-02
CPIJ004369	glucosyl transferase		-2,062	0,000	42,001	0,437	-4,713	2,445E-06
CPIJ004872	protein phosphatase 2C isoform beta		-2,028	0,003	17,620	0,534	-3,800	1,445E-04
CPIJ006314	Putative uncharacterized protein	3'-5' exonuclease domain-containing protein	-2,024	0,347	2,151	1,199	-1,688	9,140E-02
CPIJ019569	Juvenile hormone-inducible protein, putative		-2,023	0,347	2,147	1,199	-1,687	9,163E-02

CPIJ006677	Putative uncharacterized protein	Nose resistant-to-fluoxetine protein N-terminal do	-2,000	0,026	10,171	0,654	-3,060	2,210E-03
CPIJ009093	Ornithine decarboxylase		-1,997	0,000	74,162	0,359	-5,564	2,631E-08
CPIJ009256	Putative uncharacterized protein	FHA domain-containing protein	-1,994	0,148	4,927	0,877	-2,274	2,296E-02
CPIJ014363	Rrp15 protein		-1,976	0,026	10,054	0,645	-3,062	2,196E-03
CPIJ012125	FK506 binding protein		-1,974	0,155	4,753	0,880	-2,243	2,487E-02
CPIJ007583	pantetheinase precursor		-1,965	0,000	155,498	0,305	-6,440	1,191E-10
CPIJ006313	lipoate-protein ligase		-1,954	0,234	4,112	0,982	-1,989	4,667E-02
CPIJ003292	Putative uncharacterized protein	RING-type domain-containing protein	-1,931	0,259	3,317	1,012	-1,909	5,628E-02
CPIJ019818	Phosphopantothenoylcysteine decarboxylase		-1,911	0,099	6,184	0,770	-2,482	1,307E-02
CPIJ017233	Actin-binding protein ipp		-1,911	0,275	3,113	1,022	-1,870	6,145E-02
CPIJ008878	prolylcarboxypeptidase, putative		-1,883	0,004	19,020	0,515	-3,658	2,541E-04
CPIJ007633	ATP-dependent DNA helicase hus2/rqh1		-1,882	0,300	2,871	1,042	-1,805	7,100E-02
CPIJ010762	glucosyl/glucuronosyl transferases		-1,880	0,041	10,964	0,654	-2,874	4,056E-03
CPIJ016848	CYP6M14: cytochrome P450 6M14		-1,878	0,000	812,195	0,285	-6,589	4,442E-11
CPIJ012737	Nucleoporin 50kDa		-1,876	0,042	10,069	0,654	-2,866	4,151E-03
CPIJ014285	3-oxoacyl-[acyl-carrier-protein] reductase		-1,875	0,087	8,384	0,737	-2,544	1,097E-02
CPIJ019673	CYP6AG15: cytochrome P450 6AG15		-1,872	0,000	37,474	0,389	-4,812	1,497E-06
CPIJ015408	branched-chain amino acid aminotransferase		-1,866	0,000	84,528	0,304	-6,136	8,450E-10
CPIJ000761	protein expanded		-1,855	0,280	3,383	1,000	-1,855	6,365E-02
CPIJ011433	Brachyurin		-1,845	0,194	100,401	0,872	-2,117	3,427E-02
CPIJ008896	Putative uncharacterized protein	Phorbol-ester/DAG-type domain-containing prote	-1,841	0,219	4,230	0,902	-2,042	4,112E-02
CPIJ015964	Brain protein 16		-1,835	0,205	5,042	0,880	-2,084	3,713E-02
CPIJ006716	peptidoglycan recognition protein sb2		-1,835	0,105	6,420	0,749	-2,450	1,429E-02
CPIJ005330	60S ribosome subunit biogenesis protein NIP7 homolog		-1,832	0,032	11,729	0,614	-2,982	2,865E-03
CPIJ007736	Steroid dehydrogenase		-1,828	0,009	19,723	0,532	-3,435	5,926E-04
CPIJ010538	CYP9J46: cytochrome P450 9J46		-1,822	0,000	64,797	0,357	-5,112	3,196E-07
CPIJ016828	26S proteasome regulatory subunit S3		-1,818	0,158	5,535	0,813	-2,236	2,535E-02
CPIJ017594	Putative uncharacterized protein	mRNA decay factor PAT1 domain-containing pro	-1,814	0,132	6,118	0,776	-2,336	1,948E-02
CPIJ018795	14.5 kDa salivary protein, putative		-1,814	0,065	8,316	0,676	-2,682	7,323E-03
CPIJ005585	Putative uncharacterized protein	E3 ubiquitin-protein ligase E3D	-1,813	0,302	3,411	1,006	-1,801	7,163E-02
CPIJ010738	protein FAM96A		-1,812	0,004	18,044	0,490	-3,698	2,171E-04
CPIJ003123	DNA-directed RNA polymerase I 40 kDa polypeptide		-1,806	0,040	10,479	0,623	-2,898	3,755E-03
CPIJ018533	MAP kinase JNK		-1,785	0,416	2,376	1,168	-1,529	1,263E-01
CPIJ020137	Ceramidase		-1,771	0,320	3,283	1,008	-1,757	7,888E-02
CPIJ001657	Organic cation transporter protein		-1,770	0,216	4,382	0,862	-2,054	3,993E-02
CPIJ017306	Sterol desaturase		-1,767	0,016	13,947	0,547	-3,231	1,235E-03
CPIJ003814	cell cycle checkpoint protein rad17, putative		-1,765	0,431	2,149	1,186	-1,488	1,369E-01
CPIJ019825	Putative uncharacterized protein		-1,762	0,320	3,816	1,001	-1,761	7,831E-02

CPIJ020199	CYP6N20: cytochrome P450 6N20		-1,760	0,001	28,286	0,436	-4,038	5,397E-05
CPIJ009320	Ethanolamine-phosphate cytidylyltransferase		-1,750	0,001	38,588	0,415	-4,217	2,479E-05
CPIJ001316	6-phosphogluconate dehydrogenase, decarboxylating		-1,749	0,000	56,206	0,330	-5,297	1,174E-07
CPIJ006169	WD repeat protein 74		-1,748	0,060	10,041	0,643	-2,718	6,576E-03
CPIJ016145	Predicted protein		-1,747	0,028	12,274	0,578	-3,024	2,495E-03
CPIJ016651	HemK methyltransferase family member 1		-1,737	0,148	6,239	0,763	-2,277	2,276E-02
CPIJ005830	glutaredoxin, putative		-1,727	0,000	36,509	0,374	-4,621	3,821E-06
CPIJ007384	azurocidin precursor		-1,726	0,040	1022,485	0,598	-2,886	3,905E-03
CPIJ011327	Putative uncharacterized protein	HTH CENPB-type domain-containing protein	-1,723	0,141	6,794	0,748	-2,305	2,118E-02
CPIJ016855	CYP6N20: cytochrome P450 6N20		-1,722	0,000	186,436	0,282	-6,106	1,019E-09
CPIJ000031	Glutathione S-transferase		-1,714	0,000	128,763	0,273	-6,278	3,432E-10
CPIJ001160	Abc transporter		-1,713	0,000	40,813	0,360	-4,760	1,941E-06
CPIJ020264	UDP-glucuronosyltransferase 2B15 precursor		-1,704	0,019	16,595	0,539	-3,163	1,561E-03
CPIJ006115	Mck1		-1,703	0,130	6,529	0,726	-2,347	1,893E-02
CPIJ008422	Serine esterase		-1,698	0,000	84,719	0,376	-4,516	6,288E-06
CPIJ013581	Putative uncharacterized protein		-1,697	0,289	3,690	0,926	-1,832	6,691E-02
CPIJ005288	SM protein G, putative		-1,687	0,259	4,088	0,884	-1,909	5,629E-02
CPIJ018757	Gh regulated tbc protein-1		-1,683	0,222	5,131	0,827	-2,034	4,198E-02
CPIJ000616	clip-domain serine protease, putative		-1,678	0,264	4,485	0,887	-1,893	5,838E-02
CPIJ014898	protein tyrosine phosphatase n11		-1,678	0,275	4,069	0,897	-1,870	6,152E-02
CPIJ014207	Elongator complex protein 2		-1,673	0,375	2,738	1,033	-1,620	1,051E-01
CPIJ002103	Putative uncharacterized protein		-1,662	0,000	1047,502	0,304	-5,475	4,372E-08
CPIJ008163	Skeletal muscle/kidney enriched inositol 5-phosphatase		-1,657	0,015	15,358	0,509	-3,256	1,129E-03
CPIJ009805	Putative uncharacterized protein	CYTH domain-containing protein	-1,645	0,372	3,087	1,011	-1,627	1,037E-01
CPIJ003059	acyl-CoA oxidase		-1,645	0,000	202,446	0,263	-6,252	4,048E-10
CPIJ008272	BTB/POZ domain-containing protein 3		-1,643	0,289	4,112	0,897	-1,832	6,699E-02
CPIJ011633	short-chain specific acyl-CoA dehydrogenase, mitochondrial precursor		-1,640	0,008	19,104	0,469	-3,498	4,688E-04
CPIJ012130	Putative uncharacterized protein	Signal recognition particle 19 kDa protein	-1,637	0,068	11,080	0,616	-2,660	7,815E-03
CPIJ000529	Putative uncharacterized protein		-1,634	0,300	3,914	0,904	-1,808	7,067E-02
CPIJ016477	Putative uncharacterized protein		-1,623	0,133	7,693	0,696	-2,332	1,972E-02
CPIJ002156	chymotrypsin BI precursor		-1,622	0,000	268,860	0,326	-4,970	6,700E-07
CPIJ013521	Putative uncharacterized protein	Protein quiver	-1,622	0,397	2,819	1,032	-1,571	1,161E-01
CPIJ006691	insect replication protein a, putative		-1,620	0,116	7,589	0,674	-2,403	1,628E-02
CPIJ003520	Pre-mRNA-splicing factor ATP-dependent RNA helicase prp22		-1,615	0,195	5,772	0,765	-2,112	3,465E-02
CPIJ018693	Hyp37.3 putative secreted salivary gland protein		-1,612	0,001	44,508	0,383	-4,213	2,521E-05
CPIJ001219	DNA-directed RNA polymerase II 19 kDa polypeptide		-1,610	0,185	5,770	0,750	-2,145	3,192E-02
CPIJ001801	Endocuticle structural glycoprotein SgAbd-1		-1,610	0,696	48,959	1,781	-0,904	3,662E-01
CPIJ012888	rRNA processing protein Ebp2		-1,610	0,160	7,492	0,723	-2,228	2,590E-02
CPIJ019702	CYP6M16: cytochrome P450 6M16		-1,609	0,004	50,904	0,434	-3,710	2,072E-04

CPIJ011457	Putative uncharacterized protein	DUF4149 domain-containing protein	-1,609	0,000	42,890	0,375	-4,293	1,766E-05
CPIJ012908	Glutaminyl-peptide cyclotransferase		-1,597	0,238	5,332	0,809	-1,975	4,827E-02
CPIJ000840	Putative uncharacterized protein	Enoyl reductase (ER) domain-containing protein	-1,595	0,000	63,350	0,311	-5,122	3,021E-07
CPIJ015346	Glutaredoxin, grx		-1,579	0,327	3,830	0,908	-1,739	8,211E-02
CPIJ007558	Lethal(3)malignant brain tumor		-1,579	0,230	5,311	0,788	-2,004	4,511E-02
CPIJ013810	Cartilage associated protein		-1,577	0,084	9,563	0,616	-2,559	1,050E-02
CPIJ006724	tubulin alpha-1 chain		-1,576	0,000	422,770	0,222	-7,091	1,330E-12
CPIJ009355	Putative uncharacterized protein	S phase cyclin A-associated protein in the endopla	-1,576	0,420	2,645	1,043	-1,511	1,309E-01
CPIJ018515	Poly(P)/ATP NAD kinase		-1,575	0,031	16,456	0,527	-2,990	2,787E-03
CPIJ017329	Brain tumor protein		-1,573	0,406	3,059	1,015	-1,549	1,213E-01
CPIJ017386	proteasome subunit beta type 8 precursor		-1,571	0,012	26,548	0,470	-3,342	8,307E-04
CPIJ014522	lava lamp protein		-1,570	0,000	93,194	0,288	-5,445	5,184E-08
CPIJ011728	Serine/threonine protein kinase		-1,568	0,190	6,646	0,737	-2,128	3,334E-02
CPIJ010601	N-terminal acetyltransferase A complex catalytic subunit ARD1		-1,566	0,065	11,425	0,584	-2,683	7,304E-03
CPIJ005390	Putative uncharacterized protein	TP53-regulated inhibitor of apoptosis 1	-1,566	0,074	11,471	0,597	-2,622	8,734E-03
CPIJ015124	sterol carrier protein-2, putative		-1,558	0,000	1206,852	0,288	-5,411	6,267E-08
CPIJ002678	Glutathione transferase I		-1,554	0,002	36,087	0,404	-3,847	1,193E-04
CPIJ004325	Gram-negative bacteria binding protein		-1,552	0,000	99,893	0,263	-5,901	3,604E-09
CPIJ010038	Putative uncharacterized protein		-1,544	0,362	3,401	0,934	-1,654	9,816E-02
CPIJ008877	prolylcarboxypeptidase, putative		-1,542	0,001	70,689	0,386	-3,994	6,494E-05
CPIJ018976	1-acylglycerol-3-phosphate acyltransferase, putative		-1,539	0,154	7,114	0,684	-2,250	2,442E-02
CPIJ001897	Putative uncharacterized protein	MADF domain-containing protein	-1,534	0,374	3,358	0,946	-1,622	1,047E-01
CPIJ005978	Putative uncharacterized protein		-1,530	0,366	3,290	0,932	-1,642	1,005E-01
CPIJ012182	Putative uncharacterized protein	CCAAT-binding factor domain-containing proteir	-1,517	0,041	14,216	0,527	-2,881	3,962E-03
CPIJ001404	Voltage-gated ion channel		-1,515	0,155	7,788	0,675	-2,244	2,482E-02
CPIJ017177	Utp14a protein		-1,514	0,225	7,500	0,747	-2,026	4,277E-02
CPIJ013919	Xanthine dehydrogenase/oxidase		-1,508	0,000	843,774	0,279	-5,415	6,123E-08
CPIJ006512	Putative uncharacterized protein		-1,505	0,353	3,942	0,900	-1,672	9,453E-02
CPIJ013917	esterase B1 precursor		-1,504	0,000	343,526	0,296	-5,082	3,740E-07
CPIJ008466	Putative uncharacterized protein	Chitin-binding type-2 domain-containing protein	-1,498	0,303	4,364	0,834	-1,797	7,235E-02
CPIJ011855	Putative uncharacterized protein	Golgi to ER traffic protein 4	-1,493	0,258	5,333	0,780	-1,913	5,573E-02
CPIJ017390	cytosolic Fe-S cluster assembling factor CFD1		-1,491	0,100	9,907	0,602	-2,477	1,325E-02
CPIJ019233	Cerebral protein 1		-1,477	0,187	7,578	0,691	-2,137	3,260E-02
CPIJ010179	Putative uncharacterized protein	Phospholipid/glycerol acyltransferase domain-con	-1,476	0,465	2,486	1,045	-1,413	1,577E-01
CPIJ018627	Glutathione S-transferase 1-1		-1,475	0,000	255,764	0,227	-6,492	8,445E-11
CPIJ009429	Putative uncharacterized protein	ZZ-type domain-containing protein	-1,475	0,259	5,701	0,772	-1,910	5,619E-02
CPIJ000897	proteasome subunit alpha type 1		-1,466	0,005	31,078	0,402	-3,645	2,675E-04
CPIJ003580	cle7, putative		-1,464	0,171	7,300	0,669	-2,188	2,867E-02

CPIJ007385	serine-type enodpeptidase, putative		-1,460	0,000	99,093	0,323	-4,523	6,093E-06
CPIJ017188	Putative uncharacterized protein	SecA family profile domain-containing protein	-1,456	0,001	45,541	0,345	-4,216	2,488E-05
CPIJ017118	Putative uncharacterized protein	CHK kinase-like domain-containing protein	-1,452	0,022	21,237	0,464	-3,130	1,751E-03
CPIJ002430	Cytidine deaminase		-1,449	0,000	63,254	0,311	-4,653	3,277E-06
CPIJ007196	Putative uncharacterized protein	Hypoxia up-regulated protein 1	-1,445	0,000	190,825	0,262	-5,508	3,623E-08
CPIJ003799	protein suppressor of forked		-1,444	0,079	12,676	0,557	-2,592	9,545E-03
CPIJ004746	Membralin		-1,444	0,336	4,407	0,841	-1,716	8,613E-02
CPIJ000199	Putative uncharacterized protein		-1,435	0,277	5,754	0,771	-1,862	6,262E-02
CPIJ016574	Geranylgeranyl transferase type-1 subunit beta		-1,433	0,050	15,870	0,512	-2,796	5,171E-03
CPIJ000418	Putative uncharacterized protein	J domain-containing protein	-1,431	0,149	9,627	0,630	-2,271	2,313E-02
CPIJ006930	gamma-glutamyl hydrolase, putative		-1,428	0,000	50,150	0,318	-4,487	7,225E-06
CPIJ010187	Sodium/solute symporter		-1,428	0,226	6,987	0,707	-2,020	4,334E-02
CPIJ003678	U3 small nucleolar ribonucleoprotein protein IMP4		-1,427	0,232	6,252	0,715	-1,994	4,611E-02
CPIJ006359	Putative uncharacterized protein		-1,423	0,004	35,501	0,383	-3,718	2,009E-04
CPIJ005818	13 kDa ribonucleoprotein-associated protein		-1,422	0,041	30,179	0,494	-2,878	4,008E-03
CPIJ010781	secreted ferritin G subunit precursor, putative		-1,415	0,110	13,504	0,584	-2,425	1,532E-02
CPIJ011962	Multidrug resistance-associated protein 14		-1,413	0,050	19,337	0,506	-2,793	5,218E-03
CPIJ007956	Putative uncharacterized protein	Chitin-binding type-2 domain-containing protein	-1,412	0,067	15,480	0,530	-2,667	7,650E-03
CPIJ002176	Phosphatidylserine synthase		-1,410	0,570	22,709	1,201	-1,175	2,401E-01
CPIJ009449	Putative uncharacterized protein	RING-type domain-containing protein	-1,410	0,044	16,037	0,495	-2,849	4,387E-03
CPIJ005450	Lysozyme		-1,408	0,394	4,164	0,894	-1,575	1,152E-01
CPIJ011381	serine-type enodpeptidase, putative		-1,401	0,000	196,555	0,313	-4,471	7,785E-06
CPIJ001717	aspartyl/glutamyl-tRNA(Asn/Gln) amidotransferase subunit B		-1,396	0,205	7,688	0,670	-2,084	3,718E-02
CPIJ003399	Peroxiredoxins, prx-1, prx-2, prx-3		-1,396	0,009	47,463	0,405	-3,449	5,620E-04
CPIJ015151	adenylate cyclase, putative		-1,385	0,255	138,419	0,721	-1,921	5,467E-02
CPIJ009812	Xenotropic and polytropic murine leukemia virus receptor xpr1		-1,381	0,026	19,732	0,452	-3,053	2,268E-03
CPIJ004373	UDP-glucuronosyltransferase		-1,379	0,004	37,320	0,376	-3,667	2,456E-04
CPIJ011805	Putative uncharacterized protein		-1,379	0,710	5,118	1,587	-0,869	3,848E-01
CPIJ015241	Alkaline phosphatase		-1,375	0,224	8,812	0,678	-2,028	4,251E-02
CPIJ008859	Putative uncharacterized protein	ER-bound oxygenase mpaB/mpaB'/Rubber oxyge	-1,374	0,000	162,254	0,257	-5,353	8,635E-08
CPIJ015556	RpS28: 40S ribosomal protein S28		-1,373	0,398	45,041	0,875	-1,568	1,168E-01
CPIJ019364	apolipoprotein D, putative		-1,366	0,003	36,847	0,358	-3,819	1,339E-04
CPIJ006617	Putative uncharacterized protein	ascorbate ferrireductase (transmembrane	-1,365	0,000	168,286	0,269	-5,068	4,015E-07
CPIJ012519	E3 ubiquitin-protein ligase RNF123		-1,364	0,280	6,163	0,736	-1,854	6,373E-02
CPIJ015106	tonin precursor		-1,361	0,016	47,667	0,419	-3,246	1,172E-03
CPIJ005348	lipase 3 precursor		-1,358	0,038	18,232	0,466	-2,916	3,550E-03
CPIJ015650	Sulfotransferase 1A1		-1,355	0,022	24,043	0,435	-3,114	1,846E-03
CPIJ012313	RNA 3 terminal phosphate cyclase		-1,352	0,192	8,081	0,638	-2,121	3,396E-02

CPIJ001276	DEFA: defensin anti-microbial peptide		-1,351	0,327	5,536	0,778	-1,737	8,233E-02
CPIJ001564	Selenide		-1,351	0,010	52,682	0,398	-3,391	6,974E-04
CPIJ019996	Putative uncharacterized protein	Transmembrane protein 53	-1,345	0,050	23,209	0,481	-2,796	5,170E-03
CPIJ018694	Ion channel nompc		-1,345	0,004	41,967	0,361	-3,730	1,914E-04
CPIJ009980	Eukaryotic translation initiation factor 4 gamma		-1,338	0,060	26,460	0,493	-2,715	6,629E-03
CPIJ001856	Putative uncharacterized protein	Thioredoxin domain-containing protein	-1,334	0,080	16,095	0,517	-2,583	9,782E-03
CPIJ016873	Gr15: gustatory receptor Gr15		-1,334	0,256	7,574	0,696	-1,916	5,530E-02
CPIJ013525	Putative uncharacterized protein	SNF2 N-terminal domain-containing protein	-1,332	0,001	45,904	0,322	-4,140	3,479E-05
CPIJ016541	Putative uncharacterized protein		-1,331	0,171	9,344	0,608	-2,189	2,863E-02
CPIJ002043	Translocon-associated protein, delta subunit		-1,331	0,040	20,253	0,461	-2,886	3,899E-03
CPIJ004817	Sodium-dependent phosphate transporter		-1,326	0,000	89,200	0,261	-5,073	3,920E-07
CPIJ001269	Thiamine transporter		-1,326	0,367	4,467	0,809	-1,639	1,012E-01
CPIJ009421	Nucleoporin NUP53		-1,320	0,382	4,943	0,823	-1,605	1,085E-01
CPIJ004967	Uracil phosphoribosyltransferase		-1,320	0,421	3,573	0,876	-1,507	1,317E-01
CPIJ005468	Adenosine diphosphatase		-1,317	0,004	39,371	0,356	-3,702	2,137E-04
CPIJ016853	CYP6N21P: cytochrome P450 6N21P		-1,317	0,007	131,019	0,373	-3,528	4,187E-04
CPIJ003007	elongase, putative		-1,316	0,027	22,753	0,432	-3,048	2,305E-03
CPIJ003587	Toys are us		-1,315	0,172	9,346	0,601	-2,187	2,873E-02
CPIJ013322	Putative uncharacterized protein	Proteasome activator complex subunit 4	-1,314	0,060	19,896	0,484	-2,717	6,578E-03
CPIJ003094	Putative uncharacterized protein	DUF4773 domain-containing protein	-1,314	0,453	3,803	0,913	-1,440	1,500E-01
CPIJ016524	Putative uncharacterized protein	RING finger protein 141	-1,313	0,108	15,424	0,539	-2,435	1,489E-02
CPIJ016053	MICOS complex subunit MIC13		-1,311	0,371	4,773	0,805	-1,628	1,036E-01
CPIJ001739	Inosine triphosphate pyrophosphatase		-1,310	0,324	6,081	0,750	-1,746	8,075E-02
CPIJ005699	n-acetylgalactosaminyltransferase		-1,308	0,046	18,994	0,462	-2,832	4,630E-03
CPIJ016849	CYP6M12: cytochrome P450 6M12		-1,308	0,205	141,446	0,629	-2,081	3,741E-02
CPIJ000675	Putative uncharacterized protein		-1,308	0,262	6,938	0,689	-1,899	5,763E-02
CPIJ003754	Putative uncharacterized protein	FIP-RBD domain-containing protein	-1,305	0,054	16,871	0,473	-2,760	5,783E-03
CPIJ008246	Putative uncharacterized protein		-1,305	0,000	467,644	0,267	-4,892	9,990E-07
CPIJ015056	ubiquitin conjugating enzyme, putative		-1,305	0,247	7,044	0,670	-1,947	5,148E-02
CPIJ011129	CYP6N25: cytochrome P450 6N25		-1,305	0,254	7,178	0,678	-1,925	5,425E-02
CPIJ019789	Putative uncharacterized protein		-1,302	0,000	87,789	0,300	-4,334	1,462E-05
CPIJ012821	Antimeros		-1,299	0,161	9,726	0,584	-2,223	2,621E-02
CPIJ004492	sodium/potassium/calcium exchanger 3 precursor		-1,295	0,014	36,636	0,393	-3,293	9,901E-04
CPIJ015538	endonuclease G, mitochondrial precursor		-1,294	0,175	9,687	0,594	-2,177	2,951E-02
CPIJ013616	trypsin 5 precursor		-1,291	0,100	13,483	0,522	-2,476	1,329E-02
CPIJ017717	Putative uncharacterized protein		-1,291	0,000	862,121	0,281	-4,600	4,230E-06
CPIJ005306	Restin		-1,289	0,417	4,340	0,847	-1,522	1,279E-01
CPIJ002523	Spermidine synthase		-1,286	0,023	26,767	0,415	-3,103	1,917E-03
CPIJ014045	39S ribosomal protein L32, mitochondrial precursor		-1,285	0,225	8,134	0,634	-2,026	4,279E-02

CPIJ015971	26S proteasome non-ATPase regulatory subunit 11		-1,275	0,018	28,797	0,398	-3,202	1,364E-03
CPIJ009045	Putative uncharacterized protein	Fatty acid hydroxylase domain-containing protein	-1,270	0,658	7,523	1,292	-0,983	3,257E-01
CPIJ011095	TBC1 domain family member 5		-1,268	0,494	2,982	0,942	-1,347	1,780E-01
CPIJ019561	ADP-ribosylation factor, arf		-1,267	0,431	3,909	0,852	-1,488	1,369E-01
CPIJ014557	Nitrilase and fragile histidine triad fusion protein NitFhit		-1,266	0,093	14,492	0,504	-2,513	1,198E-02
CPIJ004838	26S proteasome non-ATPase regulatory subunit 3		-1,265	0,033	25,404	0,426	-2,966	3,013E-03
CPIJ001654	n-acetylgalactosaminyltransferase, putative		-1,257	0,388	4,675	0,791	-1,589	1,120E-01
CPIJ008588	mRNA turnover protein 4		-1,256	0,028	28,353	0,415	-3,028	2,464E-03
CPIJ007308	Putative uncharacterized protein	Plexus	-1,255	0,230	8,361	0,625	-2,007	4,471E-02
CPIJ006965	Putative uncharacterized protein	Protein takeout	-1,255	0,261	8,118	0,659	-1,905	5,679E-02
CPIJ002762	mediator complex, subunit, putative		-1,251	0,417	4,174	0,821	-1,524	1,276E-01
CPIJ002022	leucine rich protein, putative		-1,249	0,414	4,114	0,815	-1,533	1,253E-01
CPIJ007524	Putative uncharacterized protein	Gamma-interferon inducible lysosomal thiol reduc	-1,245	0,001	166,547	0,295	-4,220	2,440E-05
CPIJ015753	Putative uncharacterized protein	Transmembrane protein 87A	-1,245	0,302	6,777	0,691	-1,800	7,188E-02
CPIJ004176	Putative uncharacterized protein	Phosphatidylinositol-glycan biosynthesis class X p	-1,242	0,385	5,354	0,778	-1,596	1,104E-01
CPIJ002233	Sphingomyelin synthetase		-1,238	0,211	9,499	0,600	-2,065	3,891E-02
CPIJ008983	ATP-dependent RNA helicase DBP8		-1,237	0,375	5,240	0,763	-1,621	1,051E-01
CPIJ005158	Putative uncharacterized protein	Vesicle transport protein SEC20	-1,237	0,433	4,312	0,834	-1,482	1,383E-01
CPIJ019405	p15-2a protein, putative		-1,235	0,205	9,230	0,593	-2,084	3,720E-02
CPIJ002416	NIK and IKK{beta} binding protein		-1,235	0,451	4,470	0,856	-1,443	1,491E-01
CPIJ016922	Farnesol dehydrogenase		-1,230	0,029	43,394	0,409	-3,011	2,606E-03
CPIJ010526	Cysteinyl-tRNA synthetase		-1,230	0,078	23,371	0,473	-2,600	9,332E-03
CPIJ019125	ATP-binding cassette sub-family G member 4		-1,230	0,015	52,815	0,376	-3,267	1,087E-03
CPIJ009452	Monocarboxylate transporter		-1,226	0,255	7,898	0,639	-1,920	5,488E-02
CPIJ010195	Putative uncharacterized protein	Serum response factor-binding protein 1	-1,224	0,346	6,168	0,725	-1,689	9,118E-02
CPIJ011856	E3 ubiquitin-protein ligase MARCH5		-1,223	0,066	21,503	0,457	-2,674	7,506E-03
CPIJ004204	J protein type 1		-1,222	0,230	8,944	0,610	-2,005	4,497E-02
CPIJ011127	CYP4H34: cytochrome P450 4H34		-1,220	0,003	139,823	0,326	-3,744	1,811E-04
CPIJ000615	clip-domain serine protease, putative		-1,220	0,230	9,294	0,609	-2,003	4,523E-02
CPIJ013654	Putative uncharacterized protein	Synaptobrevin homolog YKT6	-1,219	0,517	2,934	0,944	-1,291	1,966E-01
CPIJ005438	exocyst complex component 6		-1,218	0,026	25,828	0,399	-3,050	2,289E-03
CPIJ012085	SF21		-1,217	0,020	31,862	0,386	-3,148	1,644E-03
CPIJ018614	Malate dehydrogenase		-1,216	0,384	5,032	0,760	-1,599	1,099E-01
CPIJ010890	Phosphatidylinositol glycan anchor biosynthesis		-1,215	0,210	10,021	0,587	-2,068	3,860E-02
CPIJ009247	E3 ubiquitin-protein ligase sina		-1,213	0,329	6,356	0,700	-1,732	8,327E-02
CPIJ017472	Organic cation transporter		-1,213	0,034	25,891	0,410	-2,961	3,065E-03
CPIJ007554	Actin		-1,212	0,362	5,480	0,733	-1,653	9,836E-02
CPIJ000881	Putative uncharacterized protein		-1,212	0,000	123,961	0,237	-5,110	3,230E-07

CPIJ009789	Putative uncharacterized protein		-1,209	0,365	6,457	0,735	-1,646	9,969E-02
CPIJ017540	Coatomer subunit delta		-1,208	0,048	28,890	0,429	-2,814	4,894E-03
CPIJ008046	Putative uncharacterized protein		-1,207	0,264	8,153	0,638	-1,894	5,826E-02
CPIJ002068	Uncharacterized protein (Fragment)	Queuosine 5'-phosphate N-glycosylase/hydrolase	-1,206	0,001	75,861	0,284	-4,253	2,112E-05
CPIJ018160	Lipid storage droplets surface binding protein 2		-1,204	0,003	86,934	0,320	-3,758	1,710E-04
CPIJ012341	Malic enzyme		-1,202	0,000	133,373	0,280	-4,297	1,727E-05
CPIJ003395	Reductase protein		-1,199	0,000	246,443	0,213	-5,638	1,725E-08
CPIJ007348	Nuclear movement protein nudC		-1,199	0,231	9,699	0,600	-1,998	4,574E-02
CPIJ001089	lactosylceramide 4-alpha-galactosyltransferase, putative		-1,196	0,195	11,728	0,566	-2,114	3,454E-02
CPIJ013082	Chitinase		-1,194	0,187	13,149	0,558	-2,138	3,250E-02
CPIJ009639	single-stranded DNA-binding protein, mitochondrial precursor		-1,193	0,419	4,684	0,789	-1,513	1,302E-01
CPIJ010599	Exonuclease nef-sp		-1,193	0,230	9,163	0,595	-2,004	4,508E-02
CPIJ004062	Putative uncharacterized protein	Protein UXT	-1,193	0,341	6,867	0,701	-1,703	8,864E-02
CPIJ004351	Zinc finger protein 92		-1,192	0,220	9,471	0,585	-2,038	4,156E-02
CPIJ002726	lipase 3 precursor		-1,188	0,675	12,139	1,256	-0,945	3,445E-01
CPIJ013727	Ad-003		-1,188	0,388	5,073	0,747	-1,589	1,120E-01
CPIJ014272	Putative uncharacterized protein	Dolichyl-diphosphooligosaccharide--protein glyco	-1,188	0,167	12,384	0,539	-2,205	2,745E-02
CPIJ004078	WD repeat protein 36		-1,183	0,230	9,106	0,591	-2,002	4,532E-02
CPIJ016097	Peroxisomal membrane protein 70 abcd3		-1,182	0,031	52,058	0,395	-2,990	2,793E-03
CPIJ005246	Abhydrolase domain-containing protein 7		-1,181	0,516	3,433	0,913	-1,294	1,957E-01
CPIJ002181	Endoplasmic reticulum-Golgi intermediate compartment protein 3		-1,176	0,374	5,572	0,725	-1,622	1,049E-01
CPIJ008811	Putative uncharacterized protein	BEACH domain-containing protein	-1,174	0,424	4,567	0,783	-1,499	1,338E-01
CPIJ004923	Putative uncharacterized protein	Thiamine transporter 1	-1,172	0,154	13,154	0,520	-2,252	2,433E-02
CPIJ004201	Mevalonate kinase		-1,170	0,143	13,311	0,509	-2,298	2,156E-02
CPIJ004056	Uncharacterized protein (Fragment)		-1,170	0,531	3,078	0,928	-1,260	2,075E-01
CPIJ002337	Ribonucleoside-diphosphate reductase small chain		-1,169	0,025	29,880	0,381	-3,069	2,150E-03
CPIJ004141	1-acyl-sn-glycerol-3-phosphate acyltransferase beta		-1,167	0,001	67,808	0,280	-4,174	2,992E-05
CPIJ017966	Putative uncharacterized protein	Stambha A	-1,165	0,007	52,483	0,331	-3,516	4,380E-04
CPIJ005508	DNA-J/hsp40		-1,165	0,001	95,438	0,274	-4,260	2,043E-05
CPIJ003082	CYP9J42: cytochrome P450 9J42		-1,165	0,000	565,602	0,265	-4,402	1,073E-05
CPIJ001233	Putative uncharacterized protein		-1,164	0,441	4,698	0,793	-1,466	1,425E-01
CPIJ011373	Putative uncharacterized protein	PNPLA domain-containing protein	-1,162	0,000	107,006	0,267	-4,355	1,333E-05
CPIJ009698	Putative uncharacterized protein	SLC41A/MgtE integral membrane domain-contai	-1,162	0,046	23,211	0,412	-2,824	4,741E-03
CPIJ014050	Predicted protein		-1,159	0,035	31,280	0,394	-2,941	3,270E-03
CPIJ016728	Putative uncharacterized protein		-1,159	0,104	15,827	0,472	-2,453	1,417E-02
CPIJ009629	Zinc finger X-linked protein ZXDA		-1,158	0,302	8,421	0,644	-1,799	7,197E-02
CPIJ008889	60 kDa heat shock protein, mitochondrial precursor		-1,158	0,417	43,500	0,762	-1,521	1,283E-01
CPIJ019363	chitinase domain-containing protein 1 precursor		-1,154	0,002	85,929	0,298	-3,875	1,065E-04

CPIJ012675	Sugar transporter		-1,152	0,004	72,945	0,314	-3,674	2,388E-04
CPIJ002197	Lysine-specific histone demethylase 1		-1,152	0,288	7,967	0,627	-1,836	6,630E-02
CPIJ006932	Ebna2 binding protein P100		-1,150	0,022	109,334	0,368	-3,126	1,772E-03
CPIJ003821	Sel1l protein		-1,145	0,001	127,036	0,275	-4,167	3,089E-05
CPIJ011412	M-phase phosphoprotein 11		-1,144	0,092	21,738	0,454	-2,519	1,175E-02
CPIJ005011	Putative uncharacterized protein	BUD13 homolog	-1,141	0,475	3,871	0,825	-1,384	1,663E-01
CPIJ005483	Putative uncharacterized protein	EGF-like domain-containing protein	-1,141	0,188	16,107	0,535	-2,134	3,284E-02
CPIJ019900	Inositol-1-monophosphatase		-1,136	0,302	9,152	0,631	-1,802	7,162E-02
CPIJ013918	esterase B1 precursor		-1,134	0,000	1156,576	0,210	-5,395	6,859E-08
CPIJ009064	Abhydrolase domain-containing protein 4		-1,132	0,052	43,994	0,407	-2,779	5,456E-03
CPIJ010981	Secretion-regulating guanine nucleotide exchange factor		-1,132	0,503	3,915	0,852	-1,329	1,837E-01
CPIJ016649	UBX domain-containing protein 8		-1,132	0,167	13,132	0,514	-2,202	2,770E-02
CPIJ013568	Exosome complex exonuclease RRP43		-1,132	0,453	4,553	0,786	-1,439	1,501E-01
CPIJ017623	Tetratricopeptide repeat protein 1		-1,128	0,469	4,119	0,805	-1,401	1,613E-01
CPIJ015379	Putative uncharacterized protein	Pseudouridine-5'-phosphate glycosidase	-1,127	0,302	8,660	0,626	-1,800	7,192E-02
CPIJ015252	Zinc carboxypeptidase		-1,124	0,067	20,987	0,421	-2,669	7,616E-03
CPIJ004646	UDP-galactose transporter		-1,123	0,048	28,646	0,400	-2,810	4,961E-03
CPIJ016406	protein-L-isoaspartate(D-aspartate) O-methyltransferase		-1,122	0,379	6,331	0,696	-1,611	1,072E-01
CPIJ005083	Pre-mRNA-splicing factor 18		-1,121	0,524	3,398	0,878	-1,278	2,014E-01
CPIJ017711	Putative uncharacterized protein	Cytochrome c oxidase assembly factor 6	-1,119	0,329	7,517	0,648	-1,728	8,394E-02
CPIJ002702	Adenylosuccinate lyase		-1,119	0,392	5,940	0,707	-1,582	1,136E-01
CPIJ001623	sorting nexin, putative		-1,118	0,228	10,852	0,555	-2,016	4,379E-02
CPIJ015911	Putative uncharacterized protein		-1,117	0,187	14,763	0,523	-2,138	3,254E-02
CPIJ003555	Cbp20: Nuclear cap-binding protein subunit 2		-1,115	0,283	9,338	0,604	-1,845	6,498E-02
CPIJ010271	Putative uncharacterized protein	C2H2-type domain-containing protein	-1,114	0,241	11,873	0,567	-1,966	4,924E-02
CPIJ005485	Thiamin pyrophosphokinase		-1,113	0,146	16,867	0,487	-2,286	2,225E-02
CPIJ014894	Putative uncharacterized protein	Protein CUSTOS	-1,113	0,389	5,889	0,701	-1,587	1,125E-01
CPIJ007327	protein disulfide-isomerase A6 precursor		-1,112	0,006	267,890	0,309	-3,594	3,252E-04
CPIJ008659	metalloproteinase, putative		-1,108	0,155	18,414	0,494	-2,245	2,475E-02
CPIJ011635	N-acetylglucosaminyl-phosphatidylinositol de-N-acetylase		-1,107	0,557	3,184	0,921	-1,201	2,296E-01
CPIJ006347	actin binding protein, putative		-1,105	0,048	37,566	0,393	-2,810	4,960E-03
CPIJ000406	CMP-sialic acid transporter		-1,105	0,241	11,913	0,563	-1,964	4,954E-02
CPIJ006730	ATP-dependent RNA helicase DDX55		-1,105	0,148	14,730	0,485	-2,279	2,267E-02
CPIJ005510	Putative uncharacterized protein	rRNA-processing protein UTP23	-1,105	0,529	3,490	0,871	-1,268	2,047E-01
CPIJ000337	isocitrate dehydrogenase cytoplasmic		-1,104	0,000	535,176	0,201	-5,501	3,777E-08
CPIJ001391	Putative uncharacterized protein		-1,103	0,323	7,942	0,631	-1,750	8,014E-02
CPIJ008456	Cystinosin		-1,102	0,000	412,156	0,197	-5,591	2,259E-08
CPIJ008904	alpha-glucosidase, putative		-1,101	0,001	179,201	0,257	-4,276	1,904E-05

CPIJ011770	Uncharacterized protein (Fragment)		-1,099	0,343	8,638	0,647	-1,699	8,940E-02
CPIJ003929	Alpha-1,3-mannosyl-glycoprotein beta-1		-1,099	0,013	42,237	0,332	-3,309	9,361E-04
CPIJ002036	Transmembrane and TPR repeat-containing protein		-1,095	0,288	8,543	0,597	-1,834	6,661E-02
CPIJ011443	diphosphoinositol polyphosphate phosphohydrolase, putative		-1,094	0,289	8,818	0,597	-1,833	6,674E-02
CPIJ015795	Putative uncharacterized protein		-1,094	0,329	7,998	0,633	-1,729	8,377E-02
CPIJ010328	39S ribosomal protein L51, mitochondrial precursor		-1,093	0,316	10,510	0,618	-1,770	7,678E-02
CPIJ020221	ferritin subunit, putative		-1,092	0,388	6,340	0,686	-1,591	1,117E-01
CPIJ016387	Putative uncharacterized protein		-1,091	0,064	23,881	0,405	-2,693	7,088E-03
CPIJ003976	gar2, putative		-1,091	0,065	24,340	0,406	-2,685	7,247E-03
CPIJ005943	Putative uncharacterized protein	Condensin-2 complex subunit H2 C-terminal dom	-1,090	0,376	6,625	0,674	-1,617	1,060E-01
CPIJ018539	Wd-repeat protein		-1,089	0,132	17,298	0,466	-2,338	1,937E-02
CPIJ016357	Putative uncharacterized protein	Methyltransferase domain-containing protein	-1,089	0,421	5,492	0,722	-1,509	1,314E-01
CPIJ007629	splicing factor, putative		-1,088	0,075	42,653	0,416	-2,618	8,835E-03
CPIJ015227	acetyl-coa acetyltransferase 2, putative		-1,087	0,220	14,910	0,533	-2,041	4,125E-02
CPIJ014539	U2-associated SR140 protein		-1,087	0,352	7,062	0,649	-1,675	9,398E-02
CPIJ000212	Putative uncharacterized protein		-1,086	0,461	5,507	0,764	-1,422	1,551E-01
CPIJ003064	Ribosomal protein S6 kinase		-1,085	0,230	11,370	0,542	-2,004	4,511E-02
CPIJ011328	GTP:AMP phosphotransferase mitochondrial		-1,083	0,121	26,213	0,455	-2,380	1,732E-02
CPIJ003818	Putative uncharacterized protein	VWFA domain-containing protein	-1,082	0,002	80,581	0,276	-3,926	8,623E-05
CPIJ010146	Putative uncharacterized protein	VWFA domain-containing protein	-1,079	0,236	14,049	0,544	-1,984	4,730E-02
CPIJ005821	Multi-sex-combs		-1,077	0,482	4,308	0,787	-1,369	1,711E-01
CPIJ017699	arginyl-tRNA-protein transferase 1		-1,075	0,532	3,810	0,856	-1,255	2,096E-01
CPIJ009388	Thermosome subunit alpha		-1,074	0,011	62,717	0,318	-3,381	7,226E-04
CPIJ005168	dolichyl-diphosphooligosaccharide protein glycotransferase		-1,072	0,110	38,717	0,442	-2,424	1,534E-02
CPIJ013731	Putative uncharacterized protein		-1,072	0,187	14,034	0,501	-2,138	3,249E-02
CPIJ004362	Lethal 87Df		-1,072	0,324	10,172	0,613	-1,748	8,052E-02
CPIJ001702	Putative uncharacterized protein		-1,071	0,595	2,730	0,954	-1,122	2,618E-01
CPIJ007179	Prosalpha4: 26S proteasome alpha 4 subunit		-1,069	0,028	44,629	0,353	-3,029	2,452E-03
CPIJ003835	Putative uncharacterized protein	Vitellogenin domain-containing protein	-1,068	0,382	7,024	0,666	-1,605	1,085E-01
CPIJ004832	loqs: loquacious		-1,068	0,082	28,335	0,415	-2,573	1,009E-02
CPIJ003870	acyl-CoA oxidase		-1,065	0,318	9,692	0,603	-1,765	7,751E-02
CPIJ010544	CYP9J33: cytochrome P450 9J33		-1,064	0,000	739,701	0,239	-4,460	8,210E-06
CPIJ000540	Putative uncharacterized protein	Neuroguidin	-1,063	0,359	7,919	0,640	-1,660	9,688E-02
CPIJ012907	Luciferin 4-monooxygenase		-1,061	0,000	248,832	0,234	-4,530	5,912E-06
CPIJ010578	Putative uncharacterized protein	C2H2-type domain-containing protein	-1,061	0,229	13,691	0,527	-2,012	4,427E-02
CPIJ003364	Splicing factor 3A subunit 2		-1,059	0,364	7,208	0,642	-1,650	9,892E-02
CPIJ000670	24-dehydrocholesterol reductase precursor		-1,058	0,000	768,590	0,216	-4,907	9,245E-07
CPIJ002829	Arsenite-resistance protein		-1,058	0,240	12,967	0,537	-1,969	4,890E-02

CPIJ000204	Putative uncharacterized protein	Folliculin	-1,055	0,455	5,764	0,735	-1,435	1,514E-01
CPIJ003176	Putative uncharacterized protein		-1,053	0,203	13,968	0,504	-2,090	3,665E-02
CPIJ009807	Bhlhzip transcription factor bigmax		-1,051	0,274	14,055	0,561	-1,873	6,103E-02
CPIJ007435	Putative uncharacterized protein	Major facilitator superfamily (MFS) profile domai	-1,050	0,155	17,445	0,468	-2,246	2,468E-02
CPIJ013683	Peroxisomal membrane protein PEX16		-1,050	0,328	9,686	0,606	-1,734	8,292E-02
CPIJ016096	Ciao1: protein CIAO1		-1,049	0,291	10,563	0,575	-1,826	6,792E-02
CPIJ014396	Putative uncharacterized protein	RAP domain-containing protein	-1,048	0,094	24,328	0,418	-2,506	1,222E-02
CPIJ015036	Syntaxin		-1,047	0,269	10,927	0,556	-1,883	5,967E-02
CPIJ014673	Putative uncharacterized protein	CRAL-TRIO domain-containing protein	-1,047	0,132	18,696	0,448	-2,336	1,951E-02
CPIJ018143	Putative uncharacterized protein		-1,046	0,369	7,201	0,641	-1,634	1,023E-01
CPIJ015682	Translation initiation factor if-2		-1,045	0,140	23,881	0,453	-2,306	2,112E-02
CPIJ008924	phospholipid-transporting atpase 1		-1,044	0,554	3,532	0,865	-1,207	2,276E-01
CPIJ004664	Putative uncharacterized protein	6-phosphofructo-2-kinase domain-containing prot	-1,042	0,040	40,672	0,360	-2,899	3,747E-03
CPIJ007234	AdoMet-dependent rRNA methyltransferase spb1		-1,042	0,024	46,880	0,337	-3,089	2,006E-03
CPIJ003550	78 kDa glucose-regulated protein precursor		-1,040	0,004	677,607	0,281	-3,702	2,141E-04
CPIJ014686	Putative uncharacterized protein	Ubiquitin carboxyl-terminal hydrolase 14	-1,037	0,133	19,279	0,445	-2,331	1,977E-02
CPIJ014052	Glutathione-s-transferase theta, gst		-1,036	0,000	170,741	0,230	-4,502	6,741E-06
CPIJ008347	Phosphatidylserine decarboxylase		-1,035	0,120	21,638	0,434	-2,387	1,701E-02
CPIJ013504	DHX9 protein		-1,035	0,509	4,574	0,790	-1,311	1,900E-01
CPIJ004472	Putative uncharacterized protein	26S proteasome non-ATPase regulatory subunit 5	-1,034	0,391	7,666	0,653	-1,584	1,132E-01
CPIJ006688	kinesin heavy chain subunit		-1,033	0,206	21,476	0,497	-2,078	3,771E-02
CPIJ001314	WD repeat protein 46		-1,032	0,185	16,153	0,481	-2,147	3,176E-02
CPIJ001891	Putative uncharacterized protein	CCHC-type domain-containing protein	-1,032	0,000	288,242	0,207	-4,993	5,940E-07
CPIJ008623	Flotillin-1		-1,030	0,035	33,738	0,350	-2,945	3,232E-03
CPIJ006316	Putative uncharacterized protein	Interferon-related developmental regulator 1	-1,030	0,197	19,389	0,489	-2,107	3,510E-02
CPIJ005611	Tumor suppressor candidate 3		-1,028	0,002	104,371	0,264	-3,889	1,009E-04
CPIJ015882	Putative uncharacterized protein	RRM domain-containing protein	-1,028	0,054	57,118	0,372	-2,761	5,769E-03
CPIJ010693	Uroporphyrinogen decarboxylase		-1,024	0,060	38,683	0,377	-2,716	6,607E-03
CPIJ009793	Glioma tumor suppressor candidate region gene 2 protein		-1,024	0,166	22,597	0,463	-2,211	2,705E-02
CPIJ006728	Putative uncharacterized protein	Methyltransferase-like protein 23	-1,024	0,136	18,602	0,441	-2,321	2,031E-02
CPIJ002605	protein serine/threonine kinase, putative		-1,023	0,263	13,052	0,540	-1,895	5,812E-02
CPIJ007439	Putative uncharacterized protein	RRM domain-containing protein	-1,022	0,456	5,982	0,713	-1,433	1,517E-01
CPIJ011778	Transcription initiation factor TFIID subunit 12		-1,022	0,450	5,576	0,707	-1,446	1,481E-01
CPIJ011687	Inosine-5-monophosphate dehydrogenase		-1,022	0,239	14,608	0,518	-1,971	4,871E-02
CPIJ018235	Prefoldin subunit 5		-1,022	0,104	21,949	0,416	-2,454	1,413E-02
CPIJ015642	ATP-dependent RNA helicase Ddx1		-1,020	0,234	12,718	0,512	-1,992	4,641E-02
CPIJ008447	Amine oxidase		-1,020	0,034	37,078	0,345	-2,954	3,139E-03
CPIJ006618	Putative uncharacterized protein	PLAC domain-containing protein	-1,019	0,413	6,921	0,663	-1,537	1,242E-01

CPIJ012839	1-acylglycerol-3-phosphate acyltransferase, putative		-1,018	0,106	22,968	0,417	-2,444	1,452E-02
CPIJ019470	3-demethylubiquinone-9 3-methyltransferase		-1,018	0,018	93,977	0,318	-3,198	1,384E-03
CPIJ001467	Putative uncharacterized protein		-1,015	0,524	4,439	0,794	-1,278	2,012E-01
CPIJ002278	S-methyl-5-thioadenosine phosphorylase		-1,015	0,078	25,635	0,391	-2,598	9,385E-03
CPIJ009487	Putative uncharacterized protein		-1,014	0,543	3,942	0,825	-1,229	2,190E-01
CPIJ009725	Putative uncharacterized protein	Pre-rRNA-processing protein RIX1 N-terminal dc	-1,014	0,412	7,788	0,659	-1,539	1,237E-01
CPIJ003764	Putative uncharacterized protein	UMA domain-containing protein	-1,014	0,353	8,214	0,606	-1,673	9,438E-02
CPIJ016921	3-oxoacyl-[acyl-carrier-protein] reductase 1		-1,014	0,629	2,680	0,962	-1,053	2,922E-01
CPIJ015942	camp-dependent protein kinase type i-beta regulatory subunit		-1,013	0,346	9,153	0,599	-1,690	9,103E-02
CPIJ014193	Putative uncharacterized protein	Chitin-binding type-2 domain-containing protein	-1,013	0,002	224,119	0,262	-3,869	1,095E-04
CPIJ006220	Putative uncharacterized protein		-1,011	0,027	54,870	0,332	-3,046	2,319E-03
CPIJ005577	Srpk		-1,011	0,532	4,013	0,805	-1,256	2,092E-01
CPIJ014091	Sorting nexin 14		-1,010	0,329	9,379	0,583	-1,732	8,319E-02
CPIJ008873	prolylcarboxypeptidase, putative		-1,010	0,000	669,192	0,199	-5,069	4,009E-07
CPIJ006918	Putative uncharacterized protein		-1,009	0,102	23,199	0,409	-2,467	1,364E-02
CPIJ001549	Putative uncharacterized protein		-1,008	0,577	3,294	0,869	-1,160	2,460E-01
CPIJ007514	ATPase family AAA domain-containing protein 3		-1,008	0,340	11,899	0,591	-1,706	8,795E-02
CPIJ001128	valacyclovir hydrolase, putative		-1,007	0,046	34,607	0,355	-2,833	4,612E-03
CPIJ020092	Putative uncharacterized protein	lactoylglutathione lyase	-1,006	0,010	78,857	0,295	-3,414	6,402E-04
CPIJ019868	Heat shock 70 kDa protein 4		-1,006	0,124	26,451	0,425	-2,366	1,797E-02
CPIJ001284	Putative uncharacterized protein	DPH-type MB domain-containing protein	-1,004	0,475	5,182	0,725	-1,385	1,660E-01
CPIJ007254	Putative uncharacterized protein	Conserved oligomeric Golgi complex subunit 3	-1,003	0,407	8,231	0,648	-1,548	1,216E-01
CPIJ011590	Putative uncharacterized protein	DUF4780 domain-containing protein	-1,002	0,132	27,253	0,428	-2,339	1,934E-02
CPIJ013872	Putative uncharacterized protein		-1,001	0,409	7,705	0,648	-1,545	1,224E-01
CPIJ019318	Putative uncharacterized protein	N-acetyltransferase domain-containing protein	-1,001	0,274	12,438	0,534	-1,874	6,089E-02
CPIJ005358	Putative uncharacterized protein		1,001	0,419	6,780	0,661	1,515	1,297E-01
CPIJ009367	Putative uncharacterized protein		1,002	0,019	47,758	0,316	3,171	1,517E-03
CPIJ010972	Zinc finger protein		1,003	0,121	40,632	0,421	2,383	1,716E-02
CPIJ005060	alpha-amylase B precursor		1,004	0,000	7686,575	0,122	8,219	2,052E-16
CPIJ004795	mitochondrial carrier protein, putative		1,006	0,077	23,055	0,386	2,604	9,212E-03
CPIJ004547	Lariat debranching enzyme		1,007	0,123	19,630	0,424	2,374	1,761E-02
CPIJ011616	serine collagenase 1 precursor, putative		1,007	0,001	332,827	0,251	4,011	6,055E-05
CPIJ020244	Putative uncharacterized protein	Solute carrier family 3 member 2 N-terminal dom:	1,011	0,079	25,626	0,390	2,589	9,627E-03
CPIJ002139	HzC4 chymotrypsinogen		1,011	0,000	3293,862	0,193	5,243	1,578E-07
CPIJ011273	39S ribosomal protein 54, mitochondrial precursor		1,015	0,080	22,823	0,393	2,582	9,809E-03
CPIJ005417	Synaptic vesicle glycoprotein 2C		1,015	0,287	2583,089	0,552	1,838	6,610E-02
CPIJ006999	Putative uncharacterized protein	3'-5' exonuclease domain-containing protein	1,016	0,283	11,032	0,550	1,846	6,485E-02
CPIJ013258	Putative uncharacterized protein		1,016	0,250	10,415	0,524	1,937	5,275E-02

CPIJ003581	Putative uncharacterized protein	Chitin-binding type-4 domain-containing protein	1,017	0,002	124,902	0,258	3,947	7,920E-05
CPIJ009131	Serpin B4		1,018	0,080	26,099	0,394	2,584	9,775E-03
CPIJ018159	Fibrinogen and fibronectin		1,019	0,002	97,947	0,264	3,863	1,118E-04
CPIJ002892	Putative uncharacterized protein	Arrestin C-terminal-like domain-containing protei	1,019	0,277	9,007	0,547	1,863	6,243E-02
CPIJ014221	CYP9M13P: cytochrome P450 9M13P		1,021	0,012	47,778	0,306	3,338	8,424E-04
CPIJ018231	carboxylesterase		1,023	0,000	234,363	0,201	5,101	3,371E-07
CPIJ004225	pancreatic triacylglycerol lipase precursor		1,023	0,000	1381,443	0,184	5,548	2,895E-08
CPIJ015646	N(4)-(Beta-N-acetylglucosaminyl)-L-asparaginase		1,024	0,167	14,777	0,465	2,204	2,755E-02
CPIJ016094	Putative uncharacterized protein	Osteopetrosis-associated transmembrane protein 1	1,024	0,015	43,376	0,315	3,255	1,132E-03
CPIJ002864	Tryptophan 2,3-dioxygenase		1,025	0,062	29,016	0,379	2,704	6,847E-03
CPIJ010230	CYP12F10: cytochrome P450 12F10		1,026	0,067	25,779	0,385	2,667	7,663E-03
CPIJ004613	importin subunit beta		1,027	0,431	5,206	0,691	1,486	1,373E-01
CPIJ016342	Putative uncharacterized protein	Chitin-binding type-2 domain-containing protein	1,027	0,006	95,957	0,287	3,577	3,469E-04
CPIJ002025	Putative uncharacterized protein	FYVE-type domain-containing protein	1,031	0,076	31,963	0,395	2,607	9,126E-03
CPIJ008243	Putative uncharacterized protein		1,031	0,015	66,743	0,316	3,265	1,094E-03
CPIJ011864	Zinc finger protein 14		1,035	0,158	17,176	0,463	2,236	2,535E-02
CPIJ013122	Phospholipase D2		1,037	0,009	69,105	0,302	3,439	5,845E-04
CPIJ012311	Histone deacetylase		1,039	0,001	258,510	0,246	4,217	2,476E-05
CPIJ005697	n-acetylgalactosaminyltransferase		1,040	0,008	44,927	0,300	3,465	5,307E-04
CPIJ001758	CYP4H38: cytochrome P450 4H38		1,041	0,000	311,361	0,238	4,380	1,187E-05
CPIJ003305	NAD(P) transhydrogenase, mitochondrial precursor		1,043	0,002	1424,059	0,265	3,935	8,320E-05
CPIJ000973	Putative uncharacterized protein	Paramyosin	1,046	0,254	9,006	0,544	1,925	5,428E-02
CPIJ010259	voltage and ligand gated potassium channel		1,050	0,048	28,637	0,373	2,814	4,896E-03
CPIJ015417	Xylulose kinase		1,053	0,089	22,424	0,416	2,531	1,136E-02
CPIJ006569	Glutaminase		1,054	0,088	27,108	0,415	2,537	1,118E-02
CPIJ001231	Putative uncharacterized protein		1,054	0,262	9,676	0,555	1,901	5,728E-02
CPIJ019347	Copper-transporting ATPase 1		1,056	0,041	27,643	0,367	2,877	4,017E-03
CPIJ002743	Putative uncharacterized protein	MD-2-related lipid-recognition domain-containing	1,057	0,000	2470,522	0,207	5,106	3,292E-07
CPIJ011963	Multidrug resistance-associated protein 1		1,057	0,236	13,064	0,534	1,982	4,750E-02
CPIJ002680	Glutathione S-transferase		1,058	0,199	13,896	0,503	2,101	3,564E-02
CPIJ018600	Putative uncharacterized protein	Potassium channel domain-containing protein	1,059	0,283	8,527	0,574	1,845	6,498E-02
CPIJ005565	Nucleosomal histone kinase 1		1,059	0,369	5,689	0,648	1,634	1,023E-01
CPIJ016645	Putative uncharacterized protein	WW domain-containing protein	1,062	0,234	9,956	0,534	1,990	4,662E-02
CPIJ000039	glucosyl/glucuronosyl transferases		1,065	0,000	136,638	0,244	4,362	1,287E-05
CPIJ000823	Putative uncharacterized protein		1,066	0,168	12,340	0,485	2,199	2,789E-02
CPIJ005956	CYP6BZ2: cytochrome P450 6BZ2		1,067	0,000	486,004	0,196	5,452	4,972E-08
CPIJ004099	ATP-dependent RNA helicase SUV3, mitochondrial precursor		1,068	0,343	7,398	0,629	1,698	8,947E-02
CPIJ009743	Troponin t, invertebrate		1,069	0,000	217,648	0,228	4,689	2,749E-06

CPIJ016310	Candidate tumor suppressor protein		1,069	0,243	11,740	0,547	1,956	5,042E-02
CPIJ001939	Putative uncharacterized protein		1,074	0,132	14,169	0,459	2,338	1,939E-02
CPIJ010609	Putative uncharacterized protein		1,075	0,341	6,111	0,631	1,705	8,828E-02
CPIJ015039	Synembryn		1,077	0,282	9,219	0,582	1,849	6,439E-02
CPIJ012207	Ammonium transporter 1		1,082	0,005	54,870	0,298	3,628	2,859E-04
CPIJ010052	Ankyrin repeat domain-containing protein 44		1,083	0,012	40,679	0,325	3,327	8,782E-04
CPIJ001335	Serine threonine-protein kinase		1,083	0,016	67,432	0,334	3,242	1,186E-03
CPIJ012511	Uncharacterized protein (Fragment)		1,084	0,003	88,021	0,287	3,778	1,579E-04
CPIJ004238	Mitochondrial brown fat uncoupling protein		1,084	0,070	21,892	0,409	2,648	8,095E-03
CPIJ005155	Putative uncharacterized protein	NTF2-related export protein	1,089	0,001	79,186	0,265	4,115	3,870E-05
CPIJ008671	Zinc-finger protein ZPR1		1,090	0,019	40,245	0,344	3,165	1,552E-03
CPIJ000698	Putative uncharacterized protein	C2H2-type domain-containing protein	1,096	0,139	13,099	0,474	2,312	2,076E-02
CPIJ005050	ATP-binding cassette sub-family A member 3		1,097	0,031	29,642	0,366	2,997	2,727E-03
CPIJ009478	CYP4D42: cytochrome P450 4D42		1,098	0,001	349,400	0,265	4,149	3,346E-05
CPIJ016881	Putative uncharacterized protein		1,102	0,276	7,336	0,590	1,867	6,188E-02
CPIJ001749	Ring canal kelch protein		1,103	0,040	27,315	0,381	2,897	3,772E-03
CPIJ003432	Aldehyde dehydrogenase		1,106	0,000	512,272	0,241	4,587	4,505E-06
CPIJ001040	Serine threonine kinase		1,107	0,221	9,975	0,544	2,034	4,191E-02
CPIJ001277	Putative uncharacterized protein		1,107	0,037	26,127	0,378	2,929	3,401E-03
CPIJ008353	Putative uncharacterized protein	Ninjurin a	1,107	0,000	457,945	0,204	5,417	6,071E-08
CPIJ001759	CYP4H40: cytochrome P450 4H40		1,107	0,000	552,667	0,210	5,277	1,311E-07
CPIJ014219	CYP9M10P: cytochrome P450 9M10-de1b		1,110	0,019	46,362	0,349	3,182	1,461E-03
CPIJ015980	Putative uncharacterized protein	Chitin-binding type-2 domain-containing protein	1,110	0,643	162,554	1,090	1,019	3,083E-01
CPIJ016229	gamma glutamyl transpeptidases		1,113	0,131	13,598	0,475	2,340	1,927E-02
CPIJ018465	Putative uncharacterized protein	Chitin-binding type-2 domain-containing protein	1,114	0,845	34,080	2,164	0,515	6,067E-01
CPIJ000024	Putative uncharacterized protein		1,116	0,225	9,217	0,551	2,025	4,291E-02
CPIJ005725	alpha-amylase A precursor		1,117	0,000	1405,428	0,203	5,504	3,708E-08
CPIJ019840	Actinin		1,117	0,012	43,754	0,334	3,341	8,349E-04
CPIJ002518	chymotrypsin 1 precursor		1,118	0,641	23,307	1,089	1,027	3,044E-01
CPIJ002236	Putative uncharacterized protein	Q rich salivary secreted protein	1,119	0,596	21,425	0,999	1,120	2,626E-01
CPIJ001189	Putative uncharacterized protein	C3H1-type domain-containing protein	1,119	0,167	11,803	0,508	2,204	2,750E-02
CPIJ011985	Putative uncharacterized protein	Solute carrier family 66 member 3	1,126	0,116	14,129	0,469	2,400	1,640E-02
CPIJ004556	Putative uncharacterized protein	E3 ubiquitin-protein ligase KCMF1	1,127	0,036	26,265	0,384	2,935	3,340E-03
CPIJ002077	Aspartyl/asparaginyl beta-hydroxylase		1,128	0,024	46,071	0,366	3,082	2,059E-03
CPIJ003989	laminin subunit alpha precursor		1,129	0,040	32,307	0,391	2,885	3,908E-03
CPIJ006761	Glucose-6-phosphate 1-dehydrogenase		1,131	0,001	68,598	0,279	4,048	5,170E-05
CPIJ017813	Spermine oxidase		1,132	0,101	15,429	0,458	2,472	1,345E-02
CPIJ016093	Putative uncharacterized protein	Osteopetrosis-associated transmembrane protein 1	1,136	0,014	41,536	0,345	3,289	1,005E-03

CPIJ011431	Putative uncharacterized protein		1,137	0,000	29072,141	0,105	10,868	1,639E-27
CPIJ009357	alkali metal ion/proton exchanger 3		1,138	0,081	20,706	0,442	2,578	9,948E-03
CPIJ002076	Aspartyl/asparaginyl beta-hydroxylase		1,143	0,066	18,905	0,427	2,676	7,444E-03
CPIJ005726	histone acetyltransferase ESA1		1,145	0,124	15,878	0,483	2,369	1,786E-02
CPIJ016046	hect E3 ubiquitin ligase, putative		1,147	0,210	9,355	0,554	2,069	3,851E-02
CPIJ020053	glutathione transferase AtGST, putative		1,150	0,002	90,070	0,300	3,838	1,243E-04
CPIJ014888	Putative uncharacterized protein	PHD finger protein 12	1,153	0,031	25,172	0,385	2,995	2,743E-03
CPIJ014220	CYP9M12: cytochrome P450 9M12		1,155	0,000	97,817	0,255	4,530	5,912E-06
CPIJ005743	Putative uncharacterized protein		1,156	0,016	31,373	0,359	3,225	1,260E-03
CPIJ006896	Titin		1,159	0,149	14,157	0,510	2,271	2,316E-02
CPIJ005632	Ganglioside induced differentiation associated protein		1,161	0,003	52,161	0,304	3,822	1,325E-04
CPIJ017722	gamma glutamyl transpeptidases		1,161	0,010	37,927	0,342	3,393	6,909E-04
CPIJ012328	GLI-Kruppel family member GLI4		1,164	0,318	6,323	0,659	1,765	7,750E-02
CPIJ007483	Putative uncharacterized protein		1,164	0,276	8,043	0,624	1,865	6,219E-02
CPIJ012368	Lipoprotein-releasing system ATP-binding protein lolD		1,165	0,218	8,584	0,570	2,045	4,082E-02
CPIJ009297	Putative uncharacterized protein	LIM zinc-binding domain-containing protein	1,165	0,054	21,385	0,422	2,760	5,784E-03
CPIJ003397	Aldo-keto reductase		1,165	0,226	8,643	0,577	2,020	4,338E-02
CPIJ002809	Putative uncharacterized protein	BAG domain-containing protein	1,166	0,262	7,622	0,614	1,899	5,761E-02
CPIJ019131	DEAD box ATP-dependent RNA helicase		1,166	0,276	7,492	0,625	1,865	6,213E-02
CPIJ000124	Putative uncharacterized protein		1,167	0,147	10,892	0,512	2,280	2,259E-02
CPIJ011575	Sugar transporter		1,170	0,218	12,496	0,572	2,045	4,085E-02
CPIJ003177	guanine nucleotide exchange factor, putative		1,170	0,282	6,386	0,632	1,851	6,419E-02
CPIJ015177	Serine/threonine-protein kinase OSR1		1,170	0,007	35,069	0,334	3,506	4,549E-04
CPIJ004209	Putative uncharacterized protein	Piezo non-specific cation channel R-Ras-binding [illegible]	1,174	0,202	10,515	0,561	2,092	3,648E-02
CPIJ014242	Putative uncharacterized protein		1,175	0,053	20,213	0,424	2,772	5,565E-03
CPIJ000191	Sugar transporter		1,181	0,000	105,702	0,242	4,884	1,037E-06
CPIJ000488	Putative uncharacterized protein		1,183	0,000	14065,078	0,111	10,649	1,754E-26
CPIJ019539	Putative uncharacterized protein	CCR4-NOT transcription complex subunit 4	1,185	0,205	9,092	0,568	2,084	3,719E-02
CPIJ001794	Putative uncharacterized protein	PEHE domain-containing protein	1,188	0,092	16,331	0,472	2,519	1,177E-02
CPIJ011562	14.5 kDa salivary peptide, putative		1,189	0,004	54,306	0,323	3,678	2,353E-04
CPIJ008557	Putative uncharacterized protein	Chitin-binding type-2 domain-containing protein	1,194	0,000	149,347	0,260	4,587	4,487E-06
CPIJ016736	cysteine-rich venom protein, putative		1,197	0,519	19,831	0,930	1,287	1,980E-01
CPIJ007727	Fibrinogen and fibronectin		1,197	0,000	196,772	0,216	5,547	2,911E-08
CPIJ017149	l(2) long form, putative		1,199	0,022	40,356	0,385	3,115	1,839E-03
CPIJ015480	Putative uncharacterized protein	C2H2-type domain-containing protein	1,199	0,316	5,147	0,678	1,768	7,698E-02
CPIJ005566	SPARC precursor		1,199	0,186	9,604	0,560	2,143	3,215E-02
CPIJ003589	Putative uncharacterized protein	Ubiquitin-like domain-containing protein	1,204	0,000	742,612	0,206	5,831	5,516E-09
CPIJ010495	carboxypeptidase N subunit 2 precursor		1,206	0,018	54,024	0,379	3,186	1,441E-03

CPIJ006929	Putative uncharacterized protein	Arginine-glutamic acid dipeptide repeats protein	1,214	0,205	7,694	0,583	2,082	3,732E-02
CPIJ008379	Putative uncharacterized protein	Metallothionein	1,215	0,002	456,902	0,315	3,861	1,128E-04
CPIJ013972	protein trithorax		1,216	0,164	10,373	0,549	2,216	2,670E-02
CPIJ007018	Putative uncharacterized protein		1,218	0,352	505,908	0,727	1,674	9,410E-02
CPIJ001475	Zinc finger protein 58		1,222	0,149	10,810	0,538	2,270	2,323E-02
CPIJ040863	Putative uncharacterized protein	Serpin domain-containing protein	1,222	0,106	11,853	0,500	2,444	1,452E-02
CPIJ002359	Myomesin		1,226	0,144	10,308	0,535	2,291	2,197E-02
CPIJ016657	short-chain dehydrogenase		1,229	0,087	14,535	0,484	2,541	1,105E-02
CPIJ018192	Putative uncharacterized protein	ADP/ATP translocase	1,229	0,000	137,339	0,246	4,991	5,992E-07
CPIJ020056	Cysteine dioxygenase		1,229	0,000	61,344	0,278	4,421	9,840E-06
CPIJ014520	Putative uncharacterized protein	G1/S-specific cyclin-D2	1,231	0,183	8,422	0,572	2,154	3,126E-02
CPIJ014779	Putative uncharacterized protein	PDZ domain-containing protein	1,237	0,047	19,963	0,438	2,823	4,761E-03
CPIJ003244	Putative uncharacterized protein	F-box domain-containing protein	1,243	0,252	5,884	0,643	1,933	5,327E-02
CPIJ019627	F-box/LRR-repeat protein 14		1,246	0,152	10,071	0,552	2,257	2,401E-02
CPIJ002937	trypsin delta/gamma precursor		1,248	0,754	69,112	1,642	0,760	4,470E-01
CPIJ017145	Putative uncharacterized protein		1,249	0,461	25,854	0,879	1,422	1,551E-01
CPIJ004411	CYP6Z12: cytochrome P450 6Z12		1,249	0,000	218,352	0,271	4,609	4,042E-06
CPIJ007673	hyp37.3 putativesecreted salivary gland protein		1,254	0,094	11,876	0,501	2,505	1,226E-02
CPIJ006120	microfibril-associated glycoprotein 4 precursor		1,255	0,023	37,518	0,405	3,098	1,950E-03
CPIJ003388	Putative uncharacterized protein		1,259	0,205	7,062	0,604	2,084	3,712E-02
CPIJ000256	peptidyl-alpha-hydroxyglycine alpha-amidating lyase 1 precursor		1,264	0,191	8,165	0,595	2,124	3,370E-02
CPIJ005824	Maternal tudor protein		1,264	0,000	61,748	0,279	4,529	5,932E-06
CPIJ019120	chitotriosidase-1 precursor		1,264	0,027	28,573	0,416	3,037	2,389E-03
CPIJ005777	synaptic vesicle protein, putative		1,267	0,703	12,289	1,428	0,887	3,751E-01
CPIJ017014	CYP6AG14: cytochrome P450 6AG14		1,268	0,001	45,121	0,306	4,146	3,390E-05
CPIJ015407	Xaa-Pro aminopeptidase 1		1,268	0,000	64,534	0,286	4,428	9,513E-06
CPIJ014179	Putative uncharacterized protein	Chitin-binding type-2 domain-containing protein	1,270	0,026	19,112	0,416	3,054	2,260E-03
CPIJ015428	CYP6Z10: cytochrome P450 6Z10		1,271	0,000	602,681	0,235	5,407	6,415E-08
CPIJ003267	sodium/chloride dependent amino acid transporter		1,272	0,000	66,499	0,290	4,381	1,179E-05
CPIJ006574	Putative uncharacterized protein	N-alpha-acetyltransferase 60	1,275	0,193	7,004	0,602	2,119	3,411E-02
CPIJ009407	Putative uncharacterized protein	Chitin-binding type-2 domain-containing protein	1,275	0,630	50,359	1,215	1,050	2,937E-01
CPIJ003974	Putative uncharacterized protein	E3 ubiquitin-protein ligase LRSAM1	1,277	0,007	35,826	0,363	3,519	4,330E-04
CPIJ001419	receptor guanylate cyclase, putative		1,281	0,148	9,010	0,563	2,275	2,293E-02
CPIJ010231	CYP12F9: cytochrome P450 12F9		1,281	0,001	83,031	0,300	4,271	1,948E-05
CPIJ007652	synaptic vesicle protein, putative		1,287	0,020	33,592	0,408	3,153	1,615E-03
CPIJ018575	fibroblast growth factor receptor 3 precursor		1,290	0,065	21,685	0,480	2,684	7,264E-03
CPIJ004339	CTL18: C-type lectin		1,294	0,634	66,146	1,242	1,042	2,976E-01
CPIJ012039	Putative uncharacterized protein	C2H2-type domain-containing protein	1,298	0,218	7,209	0,634	2,047	4,066E-02

CPIJ000348	pregnancy zone protein precursor		1,299	0,001	161,096	0,318	4,089	4,332E-05
CPIJ001265	Alkaline phosphatase		1,306	0,000	631,376	0,269	4,858	1,187E-06
CPIJ011185	deoxyribonuclease I, putative		1,312	0,000	115,006	0,290	4,531	5,881E-06
CPIJ006409	Alanine-glyoxylate aminotransferase		1,316	0,002	117,317	0,334	3,936	8,273E-05
CPIJ015854	Putative uncharacterized protein		1,318	0,081	12,691	0,511	2,579	9,904E-03
CPIJ000192	Putative uncharacterized protein		1,318	0,013	23,405	0,397	3,324	8,881E-04
CPIJ000323	Putative uncharacterized protein		1,319	0,010	25,869	0,387	3,410	6,492E-04
CPIJ013862	phosphoglucomutase		1,319	0,062	13,485	0,488	2,703	6,880E-03
CPIJ012373	virus-induced RNA, putative		1,321	0,078	14,443	0,509	2,597	9,393E-03
CPIJ019467	Zinc finger protein 572		1,322	0,171	7,431	0,604	2,189	2,860E-02
CPIJ009682	Translocator protein		1,325	0,189	6,939	0,622	2,131	3,312E-02
CPIJ001302	ryanodine receptor, putative		1,328	0,122	10,361	0,559	2,376	1,750E-02
CPIJ009742	Putative uncharacterized protein		1,332	0,018	19,657	0,416	3,202	1,363E-03
CPIJ003903	Putative uncharacterized protein	Leishmanolysin-like peptidase	1,334	0,111	9,207	0,551	2,419	1,554E-02
CPIJ006248	D-arabinose 1-dehydrogenase		1,335	0,001	53,267	0,325	4,108	3,990E-05
CPIJ012055	sodium/chloride dependent amino acid transporter, putative		1,336	0,027	24,449	0,440	3,036	2,396E-03
CPIJ006614	Putative uncharacterized protein		1,338	0,171	6,949	0,611	2,190	2,853E-02
CPIJ000853	Myosin heavy chain		1,342	0,000	451,725	0,263	5,107	3,278E-07
CPIJ008109	Putative uncharacterized protein	Nose resistant-to-fluoxetine protein N-terminal do	1,344	0,000	182,122	0,211	6,364	1,971E-10
CPIJ016224	anionic trypsin-2 precursor		1,345	0,026	22,200	0,441	3,050	2,289E-03
CPIJ017388	Zinc transporter		1,346	0,084	17,098	0,526	2,559	1,050E-02
CPIJ001742	Zinc carboxypeptidase		1,346	0,000	418,739	0,268	5,024	5,074E-07
CPIJ013259	myosin light chain 1, putative		1,354	0,000	485,916	0,232	5,828	5,614E-09
CPIJ006267	LRIM16: leucine-rich immune protein (TM)		1,355	0,018	21,603	0,424	3,198	1,386E-03
CPIJ002835	serine-pyruvate aminotransferase		1,357	0,016	42,009	0,419	3,241	1,193E-03
CPIJ002938	meprin A subunit beta precursor		1,357	0,000	408,700	0,263	5,159	2,480E-07
CPIJ015817	Putative uncharacterized protein		1,364	0,076	10,127	0,523	2,609	9,074E-03
CPIJ012040	ficolin-1 precursor		1,367	0,000	93,665	0,292	4,688	2,759E-06
CPIJ003893	Putative uncharacterized protein	Transposase Synechocystis PCC 6803 domain-cor	1,371	0,114	10,109	0,569	2,411	1,590E-02
CPIJ011382	chymotrypsin 1 precursor		1,373	0,005	31,275	0,380	3,616	2,988E-04
CPIJ006517	Putative uncharacterized protein		1,380	0,350	53,663	0,820	1,682	9,261E-02
CPIJ005655	Oxidoreductase		1,382	0,000	47,673	0,318	4,350	1,363E-05
CPIJ020016	Putative uncharacterized protein	Tubulin polyglutamylase TTLL4	1,386	0,132	7,964	0,593	2,338	1,940E-02
CPIJ005322	Putative uncharacterized protein		1,389	0,001	146,826	0,339	4,092	4,268E-05
CPIJ005432	NAD-dependent methanol dehydrogenase		1,391	0,003	27,539	0,368	3,775	1,599E-04
CPIJ000250	Putative uncharacterized protein	Microtubule-associated protein tau	1,395	0,166	7,851	0,631	2,210	2,714E-02
CPIJ005692	Putative uncharacterized protein		1,402	0,005	25,965	0,385	3,640	2,721E-04
CPIJ002358	titin, putative		1,402	0,000	122,673	0,261	5,379	7,494E-08
CPIJ010456	Cysteine dioxygenase		1,405	0,003	27,643	0,367	3,829	1,288E-04

CPIJ005641	Lethal(2)essential for life protein, l2efl		1,412	0,148	9,040	0,621	2,275	2,293E-02
CPIJ000420	protein mothers against dpp		1,413	0,059	15,680	0,518	2,728	6,379E-03
CPIJ008558	Putative uncharacterized protein	Chitin-binding type-2 domain-containing protein	1,416	0,000	959,878	0,305	4,650	3,326E-06
CPIJ011026	dehydrogenase/reductase SDR family member 7 precursor		1,425	0,000	58,824	0,284	5,026	4,997E-07
CPIJ015110	Synaptic vesicle protein		1,425	0,490	25,249	1,051	1,357	1,749E-01
CPIJ008490	Ankyrin repeat domain-containing protein 44		1,426	0,014	23,043	0,434	3,286	1,016E-03
CPIJ005492	Putative uncharacterized protein	Sushi domain-containing protein	1,431	0,000	113,836	0,248	5,759	8,445E-09
CPIJ002297	Sugar transporter		1,434	0,084	10,145	0,560	2,561	1,043E-02
CPIJ001861	Sodium/solute symporter		1,438	0,061	12,678	0,531	2,709	6,748E-03
CPIJ007535	Adhesive serine protease		1,439	0,082	13,592	0,560	2,572	1,011E-02
CPIJ002493	Cation efflux protein/ zinc transporter		1,441	0,019	20,895	0,454	3,173	1,508E-03
CPIJ007903	peritrophin-1 precursor		1,442	0,820	14,829	2,491	0,579	5,627E-01
CPIJ014296	Troponin i		1,453	0,000	265,946	0,225	6,452	1,101E-10
CPIJ008433	transmembrane emp24 domain-containing protein 1 precursor		1,466	0,078	8,939	0,564	2,598	9,389E-03
CPIJ012681	ADAM metalloprotease		1,470	0,012	18,102	0,439	3,348	8,146E-04
CPIJ011256	Putative uncharacterized protein		1,472	0,107	9,884	0,604	2,438	1,477E-02
CPIJ010068	ATP-binding cassette sub-family A member 7		1,472	0,000	497,576	0,226	6,517	7,169E-11
CPIJ004783	Putative uncharacterized protein		1,481	0,106	7,386	0,606	2,443	1,456E-02
CPIJ007777	Putative uncharacterized protein		1,482	0,094	13,217	0,591	2,508	1,212E-02
CPIJ014181	Putative uncharacterized protein	Chitin-binding type-2 domain-containing protein	1,503	0,000	1580,835	0,168	8,961	3,218E-19
CPIJ003787	Putative uncharacterized protein	Protein with signal anchor	1,509	0,224	93,528	0,744	2,028	4,255E-02
CPIJ007603	Putative uncharacterized protein	Chitin-binding type-2 domain-containing protein	1,512	0,095	9,065	0,605	2,500	1,241E-02
CPIJ003058	Troponin C		1,514	0,000	203,084	0,310	4,879	1,065E-06
CPIJ010541	CYP9J37: cytochrome P450 9J37		1,519	0,000	368,959	0,243	6,250	4,116E-10
CPIJ017511	chitotriosidase-1 precursor		1,522	0,000	779,857	0,306	4,968	6,755E-07
CPIJ011198	Threonine dehydratase/deaminase		1,522	0,473	74,451	1,095	1,390	1,646E-01
CPIJ010310	Putative uncharacterized protein	CHK kinase-like domain-containing protein	1,523	0,001	72,905	0,363	4,193	2,750E-05
CPIJ011550	tubulin alpha-2 chain		1,528	0,040	11,738	0,527	2,898	3,761E-03
CPIJ019182	Ribonuclease UK114		1,529	0,006	19,363	0,429	3,569	3,589E-04
CPIJ002534	Sodium/iodide cotransporter		1,530	0,004	32,889	0,417	3,672	2,407E-04
CPIJ001757	CYP4H39: cytochrome P450 4H39		1,530	0,000	127,621	0,249	6,142	8,155E-10
CPIJ016210	Putative uncharacterized protein	ZP domain-containing protein	1,531	0,075	9,308	0,585	2,618	8,839E-03
CPIJ006075	trypsin beta precursor		1,533	0,000	134,108	0,333	4,606	4,097E-06
CPIJ000377	Homogentisate 1,2-dioxygenase		1,536	0,027	11,851	0,505	3,040	2,362E-03
CPIJ001859	Sodium/solute symporter		1,536	0,013	21,973	0,465	3,306	9,478E-04
CPIJ016520	Suppressor of defective silencing		1,538	0,121	6,298	0,646	2,380	1,729E-02
CPIJ002740	Putative uncharacterized protein	MD-2-related lipid-recognition domain-containing	1,549	0,000	555,947	0,304	5,092	3,537E-07
CPIJ008266	Putative uncharacterized protein	Chitin-binding type-2 domain-containing protein	1,554	0,080	8,520	0,602	2,582	9,816E-03

CPIJ009656	Putative uncharacterized protein		1,561	0,000	33,892	0,349	4,474	7,671E-06
CPIJ002867	AMP dependent coa ligase, putative		1,570	0,201	229,696	0,749	2,097	3,600E-02
CPIJ018021	calcium-transporting atpase sarcoplasmic/endoplasmic reticulum type		1,575	0,228	130,614	0,782	2,014	4,400E-02
CPIJ003603	Putative uncharacterized protein	AN1-type domain-containing protein	1,578	0,001	27,773	0,380	4,154	3,260E-05
CPIJ000852	Myosin-Id		1,579	0,000	207,807	0,270	5,855	4,760E-09
CPIJ009553	Putative uncharacterized protein		1,590	0,003	25,081	0,422	3,763	1,676E-04
CPIJ011427	Putative uncharacterized protein	U2A'/phosphoprotein 32 family A C-terminal dom	1,594	0,036	14,553	0,543	2,936	3,323E-03
CPIJ016573	Aladin		1,598	0,043	10,075	0,559	2,857	4,272E-03
CPIJ001188	Putative uncharacterized protein		1,608	0,071	9,754	0,608	2,643	8,206E-03
CPIJ008467	Putative uncharacterized protein	Chitin-binding type-2 domain-containing protein	1,625	0,013	13,892	0,492	3,305	9,481E-04
CPIJ017226	Zinc finger protein 449		1,627	0,074	11,189	0,620	2,624	8,681E-03
CPIJ001230	Putative uncharacterized protein		1,629	0,008	15,761	0,467	3,488	4,869E-04
CPIJ011190	deoxyribonuclease I, putative		1,632	0,000	108,768	0,273	5,989	2,109E-09
CPIJ003277	U1 small nuclear ribonucleoprotein C		1,636	0,037	9,075	0,559	2,925	3,448E-03
CPIJ003868	Putative uncharacterized protein		1,650	0,001	25,700	0,387	4,260	2,046E-05
CPIJ010666	Putative uncharacterized protein		1,663	0,067	7,102	0,623	2,668	7,634E-03
CPIJ010173	Testis-specific protein pbs13		1,664	0,000	59,503	0,378	4,403	1,070E-05
CPIJ003384	deoxyribonuclease I, putative		1,668	0,341	77,178	0,980	1,703	8,865E-02
CPIJ018313	protein 5NUC precursor		1,668	0,000	154,123	0,267	6,254	3,990E-10
CPIJ040188	NA		1,673	0,104	6,278	0,682	2,454	1,412E-02
CPIJ018314	SALAPY1: salivary apyrase		1,675	0,000	328,534	0,235	7,118	1,093E-12
CPIJ004343	Putative uncharacterized protein	Farnesoic acid O-methyl transferase domain-conta	1,676	0,004	15,989	0,455	3,687	2,271E-04
CPIJ002138	chymotrypsinogen, putative		1,683	0,000	30,212	0,360	4,672	2,988E-06
CPIJ018233	carboxylesterase		1,707	0,000	9567,293	0,125	13,620	3,056E-42
CPIJ016344	Putative uncharacterized protein	Chitin-binding type-2 domain-containing protein	1,710	0,022	13,774	0,547	3,124	1,786E-03
CPIJ015734	Putative uncharacterized protein	Chitin-binding type-2 domain-containing protein	1,716	0,000	2392,343	0,235	7,290	3,099E-13
CPIJ002395	Dopachrome conversion enzyme		1,717	0,001	32,398	0,401	4,278	1,888E-05
CPIJ005898	Putative uncharacterized protein	Chitin-binding type-2 domain-containing protein	1,726	0,000	148,378	0,231	7,467	8,222E-14
CPIJ018848	Mitochondrial protein import protein MAS5		1,727	0,000	106,708	0,336	5,142	2,713E-07
CPIJ008555	Uncharacterized protein (Fragment)		1,737	0,046	7,619	0,614	2,827	4,704E-03
CPIJ012979	Crumbs		1,737	0,003	15,591	0,460	3,780	1,571E-04
CPIJ003274	vacuolar proton translocating ATPase 116 kDa subunit a isoform 1		1,742	0,329	73,267	1,008	1,728	8,393E-02
CPIJ012159	Sphingomyelin phosphodiesterase		1,752	0,300	28,652	0,969	1,808	7,053E-02
CPIJ009655	Elongation factor 1-alpha		1,754	0,001	28,292	0,435	4,036	5,431E-05
CPIJ004944	Origin recognition complex subunit 4		1,756	0,040	9,197	0,607	2,894	3,804E-03
CPIJ003983	Putative uncharacterized protein		1,761	0,392	31,817	1,114	1,580	1,140E-01
CPIJ039839	Putative uncharacterized protein	Cuticle protein	1,769	0,001	26,016	0,416	4,254	2,095E-05
CPIJ002361	sodium/solute symporter, putative		1,769	0,002	31,603	0,447	3,962	7,439E-05
CPIJ015813	Putative uncharacterized protein	chloroplast protein-transporting ATPase	1,774	0,002	20,635	0,460	3,856	1,152E-04

CPIJ001464	alpha-amylase A precursor		1,815	0,000	1152,509	0,273	6,653	2,870E-11
CPIJ000151	Sodium/solute symporter		1,816	0,043	10,074	0,635	2,858	4,270E-03
CPIJ001773	Synaptic vesicle protein		1,817	0,004	21,899	0,495	3,674	2,389E-04
CPIJ019593	Putative uncharacterized protein	CCHC-type domain-containing protein	1,820	0,000	72,232	0,304	5,990	2,105E-09
CPIJ004224	Pancreatic triacylglycerol lipase		1,823	0,000	430,502	0,224	8,128	4,375E-16
CPIJ006152	Putative uncharacterized protein		1,823	0,041	6,929	0,633	2,878	3,997E-03
CPIJ019606	asparagine synthetase, putative		1,830	0,001	30,913	0,452	4,044	5,264E-05
CPIJ008702	Putative uncharacterized protein	Gamma-interferon inducible lysosomal thiol reduc	1,839	0,000	30,038	0,374	4,922	8,558E-07
CPIJ008079	alpha-amylase 1 precursor		1,846	0,012	1219,730	0,552	3,342	8,323E-04
CPIJ003457	Putative uncharacterized protein	CUB domain-containing protein	1,862	0,003	14,392	0,494	3,770	1,630E-04
CPIJ007178	Putative uncharacterized protein	Peptidase C45 hydrolase domain-containing prote	1,863	0,000	73,361	0,352	5,296	1,185E-07
CPIJ009326	Endocuticle structural glycoprotein SgAbd-4		1,867	0,015	11,315	0,573	3,259	1,119E-03
CPIJ007938	Asparagine synthetase B		1,869	0,000	60,054	0,391	4,775	1,796E-06
CPIJ016326	Putative uncharacterized protein		1,870	0,072	326,123	0,711	2,633	8,475E-03
CPIJ005698	n-acetylgalactosaminyltransferase		1,874	0,000	19,137	0,433	4,331	1,485E-05
CPIJ007760	Putative uncharacterized protein		1,884	0,000	35,995	0,403	4,671	2,998E-06
CPIJ010307	Putative uncharacterized protein	CHK kinase-like domain-containing protein	1,894	0,000	190,858	0,219	8,668	4,402E-18
CPIJ017793	Elegaxobin-2		1,915	0,020	13,645	0,606	3,161	1,572E-03
CPIJ011346	Notch		1,918	0,001	16,610	0,458	4,190	2,788E-05
CPIJ000023	Putative uncharacterized protein		1,924	0,003	14,872	0,508	3,790	1,506E-04
CPIJ008523	serine-type enodpeptidase, putative		1,936	0,001	15,653	0,479	4,043	5,278E-05
CPIJ016301	Serine protease inhibitor A3G		1,941	0,009	12,925	0,566	3,430	6,038E-04
CPIJ015785	transferrin precursor		1,958	0,000	35,304	0,425	4,607	4,090E-06
CPIJ010516	Phosphoenolpyruvate carboxykinase		1,961	0,277	20,889	1,054	1,861	6,269E-02
CPIJ007828	bile salt-activated lipase precursor		1,964	0,003	13,030	0,521	3,767	1,653E-04
CPIJ004223	pancreatic triacylglycerol lipase precursor		1,972	0,000	1681,662	0,182	10,812	3,010E-27
CPIJ009976	Putative uncharacterized protein	Mannose-6-phosphate receptor domain-containing	1,973	0,000	25,356	0,401	4,919	8,703E-07
CPIJ010308	Putative uncharacterized protein	CHK kinase-like domain-containing protein	1,975	0,000	43,716	0,318	6,203	5,535E-10
CPIJ015482	Putative uncharacterized protein		1,979	0,035	6,202	0,670	2,952	3,156E-03
CPIJ017693	Putative uncharacterized protein		1,992	0,039	7,104	0,687	2,901	3,718E-03
CPIJ011298	Brachyurin		1,995	0,000	28,202	0,453	4,404	1,063E-05
CPIJ006967	Putative uncharacterized protein		1,999	0,040	7,509	0,693	2,886	3,897E-03
CPIJ012798	Putative uncharacterized protein	Cuticular protein	2,007	0,037	7,428	0,687	2,922	3,480E-03
CPIJ015858	Uncharacterized protein (Fragment)		2,014	0,006	11,805	0,561	3,588	3,334E-04
CPIJ009746	Putative uncharacterized protein		2,056	0,001	16,641	0,503	4,084	4,429E-05
CPIJ003349	abc transporter, putative		2,057	0,012	7,759	0,618	3,329	8,701E-04
CPIJ012831	Fibrinogen and fibronectin		2,057	0,000	50,881	0,402	5,122	3,030E-07
CPIJ018121	membrane-bound alkaline phosphatase precursor		2,071	0,129	222,917	0,881	2,351	1,875E-02

CPIJ003361	CYP6BY2: cytochrome P450 6BY2		2,080	0,000	59,626	0,366	5,683	1,321E-08
CPIJ009106	angiotensin-converting enzyme precursor		2,085	0,001	13,402	0,500	4,167	3,084E-05
CPIJ002604	Imaginal disc growth factor		2,091	0,015	8,438	0,641	3,259	1,117E-03
CPIJ011642	Putative uncharacterized protein		2,091	0,000	16,964	0,480	4,360	1,301E-05
CPIJ013793	Cuticle protein 8		2,107	0,000	87,448	0,286	7,365	1,774E-13
CPIJ013510	Putative uncharacterized protein		2,118	0,288	60,322	1,155	1,834	6,662E-02
CPIJ003562	Putative uncharacterized protein	AF-9 ANC1 homology domain-containing protein	2,126	0,022	7,331	0,682	3,117	1,827E-03
CPIJ011478	Lipase		2,153	0,036	5,774	0,733	2,938	3,306E-03
CPIJ009625	proclotting enzyme precursor		2,163	0,009	9,424	0,627	3,448	5,650E-04
CPIJ014743	GTP-binding protein-invertebrate		2,166	0,012	7,271	0,649	3,335	8,536E-04
CPIJ006792	spaetzle 1B		2,177	0,002	10,359	0,563	3,867	1,100E-04
CPIJ017789	Serine protease		2,178	0,000	24,050	0,482	4,519	6,223E-06
CPIJ017721	gamma-glutamyltranspeptidase precursor		2,182	0,000	15,563	0,502	4,349	1,367E-05
CPIJ013879	DNA polymerase theta		2,205	0,000	26,226	0,453	4,869	1,122E-06
CPIJ013933	Putative uncharacterized protein		2,213	0,000	25,967	0,429	5,161	2,456E-07
CPIJ000007	Chitinase		2,215	0,230	43,741	1,106	2,002	4,525E-02
CPIJ002609	protein serine/threonine kinase, putative		2,216	0,000	47,744	0,357	6,213	5,189E-10
CPIJ004417	4-hydroxyphenylpyruvate dioxygenase		2,222	0,000	22,807	0,416	5,348	8,910E-08
CPIJ002235	Putative uncharacterized protein		2,239	0,149	51,853	0,987	2,269	2,327E-02
CPIJ002629	sensory appendage protein, putative		2,252	0,000	67,736	0,394	5,718	1,075E-08
CPIJ008502	Putative uncharacterized protein	Chitin-binding type-2 domain-containing protein	2,252	0,441	8,677	1,535	1,468	1,422E-01
CPIJ013085	Sarcalumenin		2,280	0,000	32,406	0,454	5,016	5,267E-07
CPIJ011417	neurexin-4 precursor		2,304	0,004	8,022	0,627	3,676	2,374E-04
CPIJ000699	Vago: salivary Cys-rich secreted protein Vago		2,305	0,017	7,206	0,718	3,211	1,324E-03
CPIJ020229	CYP4D42: cytochrome P450 4D42		2,328	0,000	48,651	0,320	7,269	3,630E-13
CPIJ016534	Putative uncharacterized protein		2,357	0,004	7,927	0,631	3,733	1,892E-04
CPIJ010546	CYP9J34: cytochrome P450 9J34		2,377	0,000	1010,652	0,316	7,524	5,316E-14
CPIJ007082	TIM: timeless circadian protein		2,378	0,000	25,313	0,404	5,891	3,850E-09
CPIJ017285	Putative uncharacterized protein	F-box domain-containing protein	2,415	0,016	6,688	0,746	3,237	1,207E-03
CPIJ004230	Lipase		2,453	0,000	259,090	0,271	9,061	1,294E-19
CPIJ017065	NMDA-type glutamate receptor subunit 1		2,467	0,000	11,762	0,558	4,422	9,759E-06
CPIJ013609	Putative uncharacterized protein	Myofilin	2,468	0,020	40,434	0,782	3,154	1,609E-03
CPIJ001777	ataxia telangiectasia mutated, putative		2,468	0,001	10,409	0,593	4,162	3,150E-05
CPIJ013511	Putative uncharacterized protein		2,473	0,098	24,219	0,995	2,486	1,291E-02
CPIJ001560	calcium-binding protein, putative		2,490	0,045	48,721	0,878	2,836	4,570E-03
CPIJ019360	Predicted protein		2,499	0,001	9,939	0,588	4,248	2,160E-05
CPIJ011584	Putative uncharacterized protein		2,505	0,051	24,964	0,899	2,786	5,336E-03
CPIJ017156	Ankyrin repeat and SOCS box protein 13		2,508	0,000	19,433	0,490	5,124	2,996E-07
CPIJ002628	sensory appendage protein, putative		2,522	0,001	13,980	0,607	4,156	3,235E-05

CPIJ012013	serine protease inhibitor, serpin		2,531	0,000	14,308	0,576	4,392	1,122E-05
CPIJ010806	Putative uncharacterized protein	Peptidase M14 carboxypeptidase A domain-conta	2,613	0,012	48,556	0,781	3,345	8,238E-04
CPIJ017792	Serine protease		2,615	0,002	7,800	0,670	3,906	9,391E-05
CPIJ019874	salivary cysteine-rich peptide		2,653	0,000	25,753	0,420	6,323	2,559E-10
CPIJ002744	Putative uncharacterized protein	MD-2-related lipid-recognition domain-containing	2,661	0,000	171,611	0,335	7,933	2,135E-15
CPIJ002849	Putative uncharacterized protein	Single domain-containing protein	2,668	0,000	25,217	0,514	5,186	2,144E-07
CPIJ002659	Transmembrane protease		2,689	0,003	8,036	0,703	3,823	1,319E-04
CPIJ001747	tep3		2,729	0,000	39,444	0,382	7,154	8,449E-13
CPIJ017122	Putative uncharacterized protein	Superoxide dismutase copper/zinc binding domair	2,730	0,000	35,113	0,427	6,397	1,589E-10
CPIJ003372	Putative uncharacterized protein		2,765	0,002	8,134	0,698	3,959	7,528E-05
CPIJ040816	NA		2,802	0,001	7,960	0,687	4,082	4,464E-05
CPIJ016697	Leukocyte elastase inhibitor		2,805	0,001	8,869	0,664	4,226	2,377E-05
CPIJ007204	apolipophorins precursor		2,843	0,154	37,685	1,263	2,251	2,440E-02
CPIJ004730	Putative uncharacterized protein	Chitin-binding type-2 domain-containing protein	2,870	0,000	1558,105	0,280	10,251	1,168E-24
CPIJ004228	Lipase		2,927	0,000	2154,214	0,191	15,289	9,025E-53
CPIJ000094	Putative uncharacterized protein		3,166	0,001	7,020	0,742	4,266	1,988E-05
CPIJ014406	Putative uncharacterized protein		3,206	0,018	41,200	1,006	3,186	1,443E-03
CPIJ002370	Putative uncharacterized protein		3,304	0,000	7,062	0,767	4,309	1,639E-05
CPIJ011583	Putative uncharacterized protein		3,328	0,000	16,949	0,598	5,569	2,567E-08
CPIJ001282	Putative uncharacterized protein	Hexosyltransferase	3,376	0,000	8,132	0,720	4,689	2,740E-06
CPIJ005640	Heat shock protein 26		3,414	0,000	12,330	0,623	5,477	4,327E-08
CPIJ000495	Putative uncharacterized protein		3,446	0,001	380,044	0,846	4,072	4,653E-05
CPIJ010175	CYP9J48: cytochrome P450 9J48		3,455	0,000	487,500	0,748	4,618	3,879E-06
CPIJ003982	Putative uncharacterized protein		3,519	0,009	21,467	1,026	3,429	6,053E-04
CPIJ000494	Putative uncharacterized protein		3,609	0,001	814,795	0,865	4,172	3,023E-05
CPIJ012752	cysteine synthase		3,630	0,000	215,331	0,355	10,216	1,679E-24
CPIJ001812	Sugar transporter		3,642	0,000	23,129	0,550	6,623	3,527E-11
CPIJ000500	Putative uncharacterized protein		3,812	0,000	664,750	0,309	12,334	5,939E-35
CPIJ004731	Putative uncharacterized protein	Chitin-binding type-2 domain-containing protein	3,880	0,000	7,839	0,810	4,793	1,646E-06
CPIJ000093	Putative uncharacterized protein		4,390	0,000	9,343	0,855	5,137	2,786E-07
CPIJ006306	Ion channel nompc		4,429	0,000	21,873	0,608	7,282	3,283E-13
CPIJ016319	Endocuticle structural glycoprotein SgAbd-2		4,582	0,005	22,126	1,265	3,622	2,918E-04
CPIJ000496	Putative uncharacterized protein	Secreted protein	4,631	0,000	2351,552	0,785	5,897	3,694E-09
CPIJ010801	carboxypeptidase B precursor		4,666	0,000	42,604	0,490	9,532	1,540E-21
CPIJ016325	Putative uncharacterized protein	Pupal cuticle protein	5,024	0,000	22,178	0,813	6,182	6,342E-10
CPIJ012573	Actin		5,137	0,003	118,140	1,347	3,814	1,367E-04
CPIJ001979	Putative uncharacterized protein	Peptidase S1 domain-containing protein	5,943	0,000	7,371	1,289	4,611	4,003E-06
CPIJ000534	Putative uncharacterized protein		6,703	0,000	6,421	1,288	5,206	1,927E-07

CPIJ001820	larval serum protein 2 precursor	6,892	0,041	56,851	2,398	2,874	4,048E-03
CPIJ002522	farnesol dehydrogenase	7,344	0,000	18,691	1,231	5,968	2,405E-09
CPIJ013538	ficolin-1 precursor	7,644	0,000	22,331	1,231	6,209	5,326E-10
CPIJ000344	39S ribosomal protein L24, mitochondrial precursor	7,812	0,000	13,896	1,255	6,223	4,868E-10
CPIJ013861	Putative uncharacterized protein	8,552	0,000	21,898	1,226	6,975	3,053E-12
CPIJ009033	arylphorin subunit C223 precursor	9,714	NA	195,909	2,379	4,083	NA

Table S5. Gene enrichment analysis with Gene Ontology (GO) terms and InterPro protein domain descriptions using differentially expressed genes from two *Culex quinquefasciatus* Bin-resistant strains (REC and REC-2), compared to a susceptible strain (S). Genes with log2 fold change equal to or greater than 1, and false discovery rates (FDR) corrected P-values lower than 0.05 were used.
(A) REC X S up and down regulated genes.

Category	Term	Count	%	PValue	Genes	List Total	Pop Hits	Pop Total	Enrichment	Bonferroni	Benjamini	FDR
INTERPRO	IPR001128:Cytochrome P450	40	6,53	4,93E-17	CPIJ012685, CPIJ009474, CPIJ016849, CPIJ010480, CPIJ020229, CPIJ016848, CPIJ006721, CPIJ003376, CPIJ009478, CPIJ005957, CPIJ005958, CPIJ005959, CPIJ001757, CPIJ001758, CPIJ005955, CPIJ010548, CPIJ005956, CPIJ017014, CPIJ010546, CPIJ001759, CPIJ019673, CPIJ010541, CPIJ010231, CPIJ010230, CPIJ010175, CPIJ016854, CPIJ016855, CPIJ015428, CPIJ001380, CPIJ020199, CPIJ004411, CPIJ002535, CPIJ002536, CPIJ010538, CPIJ002537, CPIJ010537, CPIJ017243, CPIJ016852, CPIJ016853, CPIJ015223	546	204	14161	5,085	3,84E-14	3,85E-14	3,81E-14
INTERPRO	IPR017972:Cytochrome P450, conserved site	36	5,87	3,29E-16	CPIJ009474, CPIJ016849, CPIJ010480, CPIJ020229, CPIJ016848, CPIJ006721, CPIJ003376, CPIJ009478, CPIJ005957, CPIJ005958, CPIJ005959, CPIJ001757, CPIJ001758, CPIJ005955, CPIJ010548, CPIJ005956, CPIJ017014, CPIJ010546, CPIJ019673, CPIJ010541, CPIJ010231, CPIJ010230, CPIJ010175, CPIJ016854, CPIJ016855, CPIJ015428, CPIJ001380, CPIJ004411, CPIJ002535, CPIJ002536, CPIJ010538, CPIJ002537, CPIJ010537, CPIJ017243, CPIJ016852, CPIJ016853, CPIJ015223	546	173	14161	5,397	2,59E-13	1,28E-13	1,27E-13
GOTERM_MF_DIRECT	GO:0005506~iron ion binding	45	7,34	1,07E-15	CPIJ012685, CPIJ009474, CPIJ016849, CPIJ010480, CPIJ020229, CPIJ016848, CPIJ006721, CPIJ009637, CPIJ013919, CPIJ003376, CPIJ009478, CPIJ005957, CPIJ005958, CPIJ005959, CPIJ001757, CPIJ001758, CPIJ005955, CPIJ010548, CPIJ005956, CPIJ017014, CPIJ010546, CPIJ001759, CPIJ019673, CPIJ010541, CPIJ010231, CPIJ010230, CPIJ010175, CPIJ016854, CPIJ016855, CPIJ015428, CPIJ001380, CPIJ020056, CPIJ020199, CPIJ004411, CPIJ002535, CPIJ002536, CPIJ010538, CPIJ002537, CPIJ010537, CPIJ017243, CPIJ010456, CPIJ016852, CPIJ016853, CPIJ015223	412	251	9432	4,104	2,62E-13	2,52E-13	2,49E-13
GOTERM_MF_DIRECT	GO:0016705~oxidoreductase activity, acting on paired donc	40	6,53	5,75E-15	CPIJ012685, CPIJ009474, CPIJ016849, CPIJ010480, CPIJ020229, CPIJ016848, CPIJ006721, CPIJ003376, CPIJ009478, CPIJ005957, CPIJ005958, CPIJ005959, CPIJ001757, CPIJ001758, CPIJ005955, CPIJ010548, CPIJ005956, CPIJ017014, CPIJ010546, CPIJ001759, CPIJ019673, CPIJ010541, CPIJ010231, CPIJ010230, CPIJ010175, CPIJ016854, CPIJ016855, CPIJ015428, CPIJ001380, CPIJ020199, CPIJ004411, CPIJ002535, CPIJ002536, CPIJ010538, CPIJ002537, CPIJ010537, CPIJ017243, CPIJ016852, CPIJ016853, CPIJ015223	412	209	9432	4,381	1,36E-12	6,43E-13	6,34E-13
GOTERM_MF_DIRECT	GO:0020037~heme binding	43	7,01	8,17E-15	CPIJ012685, CPIJ009474, CPIJ016849, CPIJ010480, CPIJ020229, CPIJ016848, CPIJ006721, CPIJ003376, CPIJ009478, CPIJ002864, CPIJ005957, CPIJ005958, CPIJ005959, CPIJ001757, CPIJ001758, CPIJ005955, CPIJ010548, CPIJ005956, CPIJ017014, CPIJ010546, CPIJ001759, CPIJ019673, CPIJ010541, CPIJ010231, CPIJ010230, CPIJ010175, CPIJ016854, CPIJ016855, CPIJ015428, CPIJ007566, CPIJ001380, CPIJ020199, CPIJ004411, CPIJ001764, CPIJ002535, CPIJ002536, CPIJ010538, CPIJ002537, CPIJ010537, CPIJ017243, CPIJ016852, CPIJ016853, CPIJ015223	412	243	9432	4,051	1,94E-12	6,43E-13	6,34E-13
GOTERM_MF_DIRECT	GO:0004497~monooxygenase activity	40	6,53	9,69E-14	CPIJ012685, CPIJ009474, CPIJ016849, CPIJ010480, CPIJ020229, CPIJ016848, CPIJ006721, CPIJ003376, CPIJ009478, CPIJ005957, CPIJ005958, CPIJ005959, CPIJ001757, CPIJ001758, CPIJ005955, CPIJ010548, CPIJ005956, CPIJ017014, CPIJ010546, CPIJ001759, CPIJ019673, CPIJ010541, CPIJ010231, CPIJ010230, CPIJ010175, CPIJ016854, CPIJ016855, CPIJ015428, CPIJ001380, CPIJ020199, CPIJ004411, CPIJ002535, CPIJ002536, CPIJ010538, CPIJ002537, CPIJ010537, CPIJ017243, CPIJ016852, CPIJ016853, CPIJ015223	412	227	9432	4,034	2,29E-11	5,72E-12	5,65E-12
INTERPRO	IPR002401:Cytochrome P450, E-class, group I	30	4,89	5,70E-12	CPIJ012685, CPIJ009474, CPIJ016849, CPIJ020229, CPIJ016848, CPIJ006721, CPIJ003376, CPIJ009478, CPIJ005957, CPIJ005958, CPIJ005959, CPIJ001757, CPIJ001758, CPIJ005955, CPIJ005956, CPIJ010546, CPIJ010541, CPIJ010231, CPIJ010230, CPIJ010175, CPIJ016854, CPIJ015428, CPIJ001380, CPIJ004411, CPIJ002535, CPIJ010538, CPIJ002537, CPIJ010537, CPIJ017243, CPIJ015223	546	165	14161	4,716	4,44E-09	1,48E-09	1,47E-09
INTERPRO	IPR004045:Glutathione S-transferase, N-terminal	10	1,63	1,31E-05	CPIJ018627, CPIJ002678, CPIJ002675, CPIJ018626, CPIJ002676, CPIJ020053, CPIJ009240, CPIJ018629, CPIJ002680, CPIJ018624	546	39	14161	6,650	0,01013212	0,0025492	0,002523055
GOTERM_BP_DIRECT	GO:0006749~glutathione metabolic process	10	1,63	1,77E-05	CPIJ018627, CPIJ002678, CPIJ002675, CPIJ018626, CPIJ002676, CPIJ020053, CPIJ009240, CPIJ018629, CPIJ002680, CPIJ018624	206	40	5220	6,335	0,00283964	0,002826	0,002825995
INTERPRO	IPR004046:Glutathione S-transferase, C-terminal	9	1,47	3,42E-05	CPIJ018627, CPIJ002678, CPIJ002675, CPIJ018626, CPIJ002676, CPIJ020053, CPIJ009240, CPIJ018629, CPIJ018624	546	34	14161	6,865	0,02631467	0,00445015	0,004404504

GOTERM_CC_DIRECT	GO:0005789~endoplasmic reticulum membrane	15	2,45	6,49E-04	CPIJ016855, CPIJ010480, CPIJ005168, CPIJ003198, CPIJ005257, CPIJ006347, CPIJ011727, CPIJ005137, CPIJ002176, CPIJ001505, CPIJ010538, CPIJ002536, CPIJ010548, CPIJ017014, CPIJ018234, CPIJ019673	272	146	7637	2,885	0,03882279	0,03958363	0,039583632
GOTERM_MF_DIRECT	GO:0052689~carboxylic ester hydrolase activity	9	1,47	8,56E-04	CPIJ007828, CPIJ007824, CPIJ002073, CPIJ013917, CPIJ016341, CPIJ015373, CPIJ013918	412	47	9432	4,384	0,18289932	0,04038131	0,039867987
GOTERM_BP_DIRECT	GO:0005975~carbohydrate metabolic process	17	2,77	5,54E-04	CPIJ012204, CPIJ011854, CPIJ008074, CPIJ001041, CPIJ008853, CPIJ004324, CPIJ008531, CPIJ008079, CPIJ004323, CPIJ002066, CPIJ003661, CPIJ000007, CPIJ009935, CPIJ008959, CPIJ013082, CPIJ013173, CPIJ016362	206	162	5220	2,659	0,08538217	0,04433503	0,044335027
INTERPRO	IPR004119:Protein of unknown function DUF227	10	1,63	6,43E-04	CPIJ012697, CPIJ012699, CPIJ010972, CPIJ004173, CPIJ012700, CPIJ010308, CPIJ012702, CPIJ010307, CPIJ010305, CPIJ010313	546	63	14161	4,117	0,39398049	0,07161784	0,070883294
INTERPRO	IPR002403:Cytochrome P450, E-class, group IV	6	0,98	7,95E-04	CPIJ016855, CPIJ010480, CPIJ002536, CPIJ010538, CPIJ010548, CPIJ017014, CPIJ019673	546	20	14161	7,781	0,46167169	0,0774794	0,076684744
GOTERM_MF_DIRECT	GO:0008237~metallopeptidase activity	9	1,47	0,00217013	CPIJ012036, CPIJ001049, CPIJ039829, CPIJ009107, CPIJ001052, CPIJ009106, CPIJ001050, CPIJ002590, CPIJ004086	412	54	9432	3,816	0,40112777	0,08535841	0,084273349
GOTERM_CC_DIRECT	GO:0016021~integral component of membrane	142	23,16	0,00386851	CPIJ006361, CPIJ016849, CPIJ016848, CPIJ002043, CPIJ009637, CPIJ008944, CPIJ005957, CPIJ005958, CPIJ005959, CPIJ010629, CPIJ008946, CPIJ005955, CPIJ005956, CPIJ000426, CPIJ013173, CPIJ012364, CPIJ010068, CPIJ010067, CPIJ007581, CPIJ011962, CPIJ011602, CPIJ005168, CPIJ015809, CPIJ006776, CPIJ011727, CPIJ008679, CPIJ000670, CPIJ000032, CPIJ003267, CPIJ002176, CPIJ003661, CPIJ003305, CPIJ001764, CPIJ002336, CPIJ018192, CPIJ004238, CPIJ005728, CPIJ000037, CPIJ007505, CPIJ000039, CPIJ002858, CPIJ000316, CPIJ018234, CPIJ011040, CPIJ005050, CPIJ013752, CPIJ010480, CPIJ011457, CPIJ014208, CPIJ011854, CPIJ008283, CPIJ005697, CPIJ005214, CPIJ004489, CPIJ005692, CPIJ003552, CPIJ006267, CPIJ009939, CPIJ004369, CPIJ001812, CPIJ005612, CPIJ017039, CPIJ018126, CPIJ013401, CPIJ012036, CPIJ011346, CPIJ013923, CPIJ008853, CPIJ001380, CPIJ004373, CPIJ005468, CPIJ002591, CPIJ000611, CPIJ005229, CPIJ010538, CPIJ010537, CPIJ010259, CPIJ004817, CPIJ010412, CPIJ014970, CPIJ017721, CPIJ012204, CPIJ005191, CPIJ008107, CPIJ007652, CPIJ003579, CPIJ019193, CPIJ008109, CPIJ000988, CPIJ010546, CPIJ000906, CPIJ010145, CPIJ010541, CPIJ020199, CPIJ003102, CPIJ004795, CPIJ006575, CPIJ006617, CPIJ002777, CPIJ002535, CPIJ016093, CPIJ017065, CPIJ016097, CPIJ016094, CPIJ002536, CPIJ002537, CPIJ012055, CPIJ018314, CPIJ005492, CPIJ003198, CPIJ005257, CPIJ006347, CPIJ008647, CPIJ005137, CPIJ009976, CPIJ003512, CPIJ017592, CPIJ010842, CPIJ001859, CPIJ012065, CPIJ015730, CPIJ012066, CPIJ012068, CPIJ010175, CPIJ011026, CPIJ012511, CPIJ007566, CPIJ008812, CPIJ009225, CPIJ003007, CPIJ001747, CPIJ001505, CPIJ006912, CPIJ040083, CPIJ000415, CPIJ019789, CPIJ015102	272	3355	7637	1,188	0,21056429	0,11798962	0,117989625
INTERPRO	IPR015897:CHK kinase-like	9	1,47	0,00160762	CPIJ012697, CPIJ012699, CPIJ004173, CPIJ012700, CPIJ010308, CPIJ012702, CPIJ010307, CPIJ010305, CPIJ010313	546	58	14161	4,025	0,71445175	0,13112642	0,129781533
INTERPRO	IPR002213:UDP-glucuronosyl/UDP-glucosyltransferase	7	1,14	0,00169527	CPIJ004369, CPIJ000037, CPIJ000039, CPIJ013923, CPIJ004373, CPIJ000032, CPIJ003695	546	34	14161	5,340	0,73332802	0,13112642	0,129781533
INTERPRO	IPR002110:Ankyrin repeat	14	2,28	0,00184922	CPIJ010052, CPIJ008490, CPIJ013539, CPIJ003373, CPIJ015938, CPIJ019550, CPIJ006569, CPIJ006306, CPIJ009924, CPIJ017156, CPIJ000716, CPIJ018694, CPIJ018744	546	133	14161	2,730	0,76351606	0,13112642	0,129781533
INTERPRO	IPR002402:Cytochrome P450, E-class, group II	5	0,82	0,00211045	CPIJ016849, CPIJ001759, CPIJ016852, CPIJ020199, CPIJ016853	546	15	14161	8,645	0,80713812	0,13717951	0,135772545
INTERPRO	IPR019826:Carboxylesterase type B, active site	8	1,31	0,0025399	CPIJ007828, CPIJ007824, CPIJ002073, CPIJ013917, CPIJ016341, CPIJ013918	546	49	14161	4,234	0,8620825	0,15205234	0,150492832
INTERPRO	IPR006195:Aminoacyl-tRNA synthetase, class II	5	0,82	0,00272914	CPIJ016525, CPIJ017452, CPIJ010486, CPIJ015476, CPIJ015212	546	16	14161	8,105	0,88103311	0,15205234	0,150492832
GOTERM_MF_DIRECT	GO:0004180~carboxypeptidase activity	6	0,98	0,0088733	CPIJ009107, CPIJ009106, CPIJ008878, CPIJ004086, CPIJ008877, CPIJ006124	412	30	9432	4,579	0,87796463	0,2913986	0,287694381
GOTERM_MF_DIRECT	GO:0008194~UDP-glycosyltransferase activity	5	0,82	0,00987792	CPIJ000037, CPIJ000039, CPIJ013923, CPIJ000032, CPIJ003695	412	20	9432	5,723	0,90393967	0,2913986	0,287694381
GOTERM_CC_DIRECT	GO:0043231~intracellular membrane-bounded organelle	4	0,65	0,01756411	CPIJ014889, CPIJ016852, CPIJ016853	272	16	7637	7,019	0,66072078	0,357137	0,357137002
INTERPRO	IPR004843:Metallophosphoesterase domain	6	0,98	0,00705829	CPIJ012159, CPIJ001371, CPIJ009620, CPIJ018314, CPIJ018313	546	32	14161	4,863	0,99598576	0,3670312	0,363266774
GOTERM_BP_DIRECT	GO:0000027~ribosomal large subunit assembly	3	0,49	0,00873956	CPIJ009793, CPIJ008588, CPIJ010145	206	4	5220	19,005	0,75664971	0,46610972	0,466109721
INTERPRO	IPR011032:GroES-like	5	0,82	0,01249398	CPIJ003802, CPIJ003801, CPIJ013381, CPIJ007228, CPIJ005595	546	24	14161	5,403	0,99994422	0,57325305	0,567373529
INTERPRO	IPR024571:Domain of unknown function DUF3358	5	0,82	0,01249398	CPIJ012036, CPIJ001049, CPIJ039829, CPIJ001052, CPIJ001050	546	24	14161	5,403	0,99994422	0,57325305	0,567373529
GOTERM_MF_DIRECT	GO:0004177~aminopeptidase activity	5	0,82	0,02483333	CPIJ012036, CPIJ001049, CPIJ039829, CPIJ001052, CPIJ001050	412	26	9432	4,403	0,99735389	0,5967337	0,589148104
GOTERM_MF_DIRECT	GO:0016671~oxidoreductase activity, acting on a sulfur gro	3	0,49	0,02528533	CPIJ008702, CPIJ007525, CPIJ007524	412	6	9432	11,447	0,99762813	0,5967337	0,589148104
GOTERM_MF_DIRECT	GO:0051082~unfolded protein binding	8	1,31	0,02794706	CPIJ018848, CPIJ004080, CPIJ005508, CPIJ011246, CPIJ002450, CPIJ018377, CPIJ007376, CPIJ009388	412	68	9432	2,693	0,998756	0,59959136	0,591969439
INTERPRO	IPR014782:Peptidase M1, membrane alanine aminopeptidas	5	0,82	0,0144313	CPIJ012036, CPIJ001049, CPIJ039829, CPIJ001052, CPIJ001050	546	25	14161	5,187	0,99998792	0,59998038	0,593826737
INTERPRO	IPR003010:Carbon-nitrogen hydrolase	4	0,65	0,01502853	CPIJ007581, CPIJ014557, CPIJ017593, CPIJ017592	546	14	14161	7,410	0,99999247	0,59998038	0,593826737

INTERPRO	IPR002018:Carboxylesterase, type B	9	1,47	0,01538411	CPIJ007828, CPIJ007824, CPIJ002073, CPIJ013917, CPIJ016341, CPIJ013918, CPIJ008422	546	84	14161	2,779	0,99999431	0,59998038	0,593826737
INTERPRO	IPR004911:Gamma interferon inducible lysosomal thiol redu	3	0,49	0,02000595	CPIJ008702, CPIJ007525, CPIJ007524	546	6	14161	12,968	0,99999985	0,60017859	0,594022911
INTERPRO	IPR004364:Aminoacyl-tRNA synthetase, class II (D/K/N)	3	0,49	0,02000595	CPIJ017452, CPIJ015476, CPIJ015212	546	6	14161	12,968	0,99999985	0,60017859	0,594022911
INTERPRO	IPR023333:Proteasome B-type subunit	3	0,49	0,02000595	CPIJ008264, CPIJ001361, CPIJ017386	546	6	14161	12,968	0,99999985	0,60017859	0,594022911
INTERPRO	IPR005814:Aminotransferase class-III	3	0,49	0,02000595	CPIJ008729, CPIJ006409, CPIJ004400	546	6	14161	12,968	0,99999985	0,60017859	0,594022911
INTERPRO	IPR002334:Dol/Ves 1 allergen	3	0,49	0,02000595	CPIJ004228, CPIJ001035	546	6	14161	12,968	0,99999985	0,60017859	0,594022911
INTERPRO	IPR016050:Proteasome, beta-type subunit, conserved site	3	0,49	0,02000595	CPIJ008264, CPIJ001361, CPIJ017386	546	6	14161	12,968	0,99999985	0,60017859	0,594022911
GOTERM_CC_DIRECT	GO:0005777~peroxisome	5	0,82	0,04089751	CPIJ001556, CPIJ003456, CPIJ013919, CPIJ000670	272	37	7637	3,794	0,92169923	0,62368708	0,62368708
GOTERM_MF_DIRECT	GO:0004553~hydrolase activity, hydrolyzing O-glycosyl cor	6	0,98	0,03467925	CPIJ000007, CPIJ013082, CPIJ008531, CPIJ004324, CPIJ004323, CPIJ002066	412	42	9432	3,270	0,99975873	0,64699576	0,638771238
GOTERM_MF_DIRECT	GO:0016798~hydrolase activity, acting on glycosyl bonds	5	0,82	0,0356396	CPIJ012159, CPIJ012204, CPIJ011854, CPIJ013173, CPIJ016362	412	29	9432	3,947	0,99980924	0,64699576	0,638771238
GOTERM_BP_DIRECT	GO:0006457~protein folding	9	1,47	0,01663414	CPIJ018848, CPIJ004080, CPIJ005508, CPIJ011246, CPIJ002450, CPIJ018377, CPIJ007228, CPIJ007376, CPIJ009388	206	84	5220	2,715	0,9328362	0,66536568	0,665365675
GOTERM_CC_DIRECT	GO:0005783~endoplasmic reticulum	5	0,82	0,056254	CPIJ005690, CPIJ002043, CPIJ007327, CPIJ006776, CPIJ005974	272	41	7637	3,424	0,97074752	0,68629877	0,686298771
INTERPRO	IPR013780:Glycosyl hydrolase, family 13, all-beta	6	0,98	0,02391903	CPIJ012204, CPIJ011854, CPIJ013173, CPIJ008079, CPIJ015654, CPIJ002066	546	43	14161	3,619	0,99999999	0,69099426	0,683907137
GOTERM_MF_DIRECT	GO:0016740~transferase activity	11	1,79	0,04194791	CPIJ018627, CPIJ002678, CPIJ002675, CPIJ018626, CPIJ002676, CPIJ020053, CPIJ018629, CPIJ005168, CPIJ002680, CPIJ018624, CPIJ010486	412	123	9432	2,047	0,99995946	0,70096987	0,692059233
GOTERM_MF_DIRECT	GO:0008241~peptidyl-dipeptidase activity	3	0,49	0,04455317	CPIJ009107, CPIJ009106, CPIJ004086	412	8	9432	8,585	0,99997868	0,70096987	0,692059233
INTERPRO	IPR017853:Glycoside hydrolase, superfamily	10	1,63	0,025947	CPIJ012204, CPIJ000007, CPIJ011854, CPIJ013082, CPIJ001041, CPIJ013173, CPIJ016362, CPIJ008531, CPIJ008079, CPIJ002066	546	110	14161	2,358	1	0,72280935	0,715395917
GOTERM_BP_DIRECT	GO:0042254~ribosome biogenesis	4	0,65	0,02290052	CPIJ012888, CPIJ007556, CPIJ005818, CPIJ010674	206	16	5220	6,335	0,97600372	0,73281666	0,732816656
INTERPRO	IPR004365:Nucleic acid binding, OB-fold, tRNA/helicase-ty	3	0,49	0,02730157	CPIJ017452, CPIJ015476, CPIJ015212	546	7	14161	11,115	1	0,7343181	0,72678663
GOTERM_MF_DIRECT	GO:0016787~hydrolase activity	10	1,63	0,05276194	CPIJ007581, CPIJ017592, CPIJ011190, CPIJ003384, CPIJ005468, CPIJ011185, CPIJ018314, CPIJ015662, CPIJ007311	412	111	9432	2,062	0,99999722	0,77115764	0,761354788
GOTERM_MF_DIRECT	GO:0004298~threonine-type endopeptidase activity	3	0,49	0,0556631	CPIJ008264, CPIJ001361, CPIJ017386	412	9	9432	7,631	0,99999865	0,77115764	0,761354788
GOTERM_MF_DIRECT	GO:0071949~FAD binding	5	0,82	0,05881711	CPIJ001556, CPIJ009956, CPIJ013919, CPIJ000670	412	34	9432	3,367	0,99999939	0,77115764	0,761354788
INTERPRO	IPR001353:Proteasome, subunit alpha/beta	4	0,65	0,03007295	CPIJ008264, CPIJ001361, CPIJ017386, CPIJ007179	546	18	14161	5,764	1	0,78189665	0,773877201
GOTERM_BP_DIRECT	GO:0006629~lipid metabolic process	8	1,31	0,03254897	CPIJ004228, CPIJ002103, CPIJ004230, CPIJ010979, CPIJ001035, CPIJ004224, CPIJ004223	206	78	5220	2,599	0,99514412	0,83112017	0,831120168
GOTERM_BP_DIRECT	GO:0006807~nitrogen compound metabolic process	4	0,65	0,03636151	CPIJ007581, CPIJ014557, CPIJ017593, CPIJ017592	206	19	5220	5,335	0,99742856	0,83112017	0,831120168
GOTERM_BP_DIRECT	GO:0007017~microtubule-based process	4	0,65	0,04157476	CPIJ003259, CPIJ015623, CPIJ006724, CPIJ011550	206	20	5220	5,068	0,99892632	0,83149526	0,831495257
INTERPRO	IPR019742:Alpha-2-macroglobulin, conserved site	3	0,49	0,03548659	CPIJ001747	546	8	14161	9,726	1	0,86498561	0,856113961
INTERPRO	IPR001548:Peptidase M2, peptidyl-dipeptidase A	3	0,49	0,03548659	CPIJ009107, CPIJ009106, CPIJ004086	546	8	14161	9,726	1	0,86498561	0,856113961
GOTERM_CC_DIRECT	GO:0005730~nucleolus	6	0,98	0,08853239	CPIJ009793, CPIJ012888, CPIJ007556, CPIJ005818, CPIJ010674, CPIJ008588	272	67	7637	2,514	0,99649881	0,89737837	0,897378369
INTERPRO	IPR020837:Fibrinogen, conserved site	4	0,65	0,03970883	CPIJ006041, CPIJ007727, CPIJ014772, CPIJ012829	546	20	14161	5,187	1	0,93857226	0,928945881
GOTERM_MF_DIRECT	GO:0000166~nucleotide binding	5	0,82	0,0757798	CPIJ011687, CPIJ006734, CPIJ018314, CPIJ018313	412	37	9432	3,094	0,99999999	0,94126494	0,929299703
GOTERM_MF_DIRECT	GO:0004866~endopeptidase inhibitor activity	3	0,49	0,08032723	CPIJ001747	412	11	9432	6,244	1	0,94786127	0,935812181
INTERPRO	IPR006047:Glycosyl hydrolase, family 13, catalytic domain	5	0,82	0,0442756	CPIJ012204, CPIJ011854, CPIJ013173, CPIJ016362, CPIJ008079	546	35	14161	3,705	1	0,9495454	0,939806469
INTERPRO	IPR002890:Alpha-2-macroglobulin, N-terminal	3	0,49	0,044482	CPIJ001747	546	9	14161	8,645	1	0,9495454	0,939806469
INTERPRO	IPR020843:Polyketide synthase, enoylreductase	4	0,65	0,04504254	CPIJ003802, CPIJ003801, CPIJ013381, CPIJ005595	546	21	14161	4,940	1	0,9495454	0,939806469
INTERPRO	IPR001930:Peptidase M1, alanine aminopeptidase/leukotrie	4	0,65	0,04504254	CPIJ001049, CPIJ039829, CPIJ001052, CPIJ001050	546	21	14161	4,940	1	0,9495454	0,939806469
GOTERM_MF_DIRECT	GO:0017172~cysteine dioxygenase activity	2	0,33	0,08525576	CPIJ010456, CPIJ020056	412	2	9432	22,893	1	0,95811235	0,945932958
INTERPRO	IPR020845:AMP-binding, conserved site	5	0,82	0,04835328	CPIJ011600, CPIJ002867, CPIJ016639, CPIJ000426, CPIJ015670	546	36	14161	3,602	1	0,9925146	0,982334965
INTERPRO	IPR014710:RmlC-like jelly roll fold	5	0,82	0,05263128	CPIJ003198, CPIJ010259, CPIJ010456, CPIJ020056	546	37	14161	3,505	1	1	0,991014121
INTERPRO	IPR009048:Alpha-macroglobulin, receptor-binding	3	0,49	0,05421356	CPIJ001747	546	10	14161	7,781	1	1	0,991014121
INTERPRO	IPR008334:5'-Nucleotidase, C-terminal	3	0,49	0,05421356	CPIJ018314, CPIJ018313	546	10	14161	7,781	1	1	0,991014121
INTERPRO	IPR001599:Alpha-2-macroglobulin	3	0,49	0,05421356	CPIJ001747	546	10	14161	7,781	1	1	0,991014121
INTERPRO	IPR020846:Major facilitator superfamily domain	9	1,47	0,05658855	CPIJ001812, CPIJ014208, CPIJ008946, CPIJ000988, CPIJ008812, CPIJ008944, CPIJ004817, CPIJ007652	546	108	14161	2,161	1	1	0,991014121
INTERPRO	IPR025110:Domain of unknown function DUF4009	5	0,82	0,05710842	CPIJ011600, CPIJ002867, CPIJ016639, CPIJ000426, CPIJ015670	546	38	14161	3,413	1	1	0,991014121
INTERPRO	IPR006179:5'-Nucleotidase/apyrase	3	0,49	0,06461149	CPIJ018314, CPIJ018313	546	11	14161	7,073	1	1	0,991014121
INTERPRO	IPR002194:Chaperonin TCP-1, conserved site	3	0,49	0,06461149	CPIJ004080, CPIJ002450, CPIJ018377	546	11	14161	7,073	1	1	0,991014121
INTERPRO	IPR011626:A-macroglobulin complement component	3	0,49	0,06461149	CPIJ001747	546	11	14161	7,073	1	1	0,991014121
INTERPRO	IPR011625:Alpha-2-macroglobulin, N-terminal 2	3	0,49	0,06461149	CPIJ001747	546	11	14161	7,073	1	1	0,991014121
INTERPRO	IPR000808:Mrp, conserved site	2	0,33	0,07549341	CPIJ017390, CPIJ014142	546	2	14161	25,936	1	1	0,991014121
INTERPRO	IPR010300:Cysteine dioxygenase type I	2	0,33	0,07549341	CPIJ010456, CPIJ020056	546	2	14161	25,936	1	1	0,991014121
INTERPRO	IPR019172:Osteopetrosis-associated transmembrane protein	2	0,33	0,07549341	CPIJ016093, CPIJ016094	546	2	14161	25,936	1	1	0,991014121
INTERPRO	IPR003950:Potassium channel, voltage-dependent, ELK	2	0,33	0,07549341	CPIJ010259	546	2	14161	25,936	1	1	0,991014121
INTERPRO	IPR020904:Short-chain dehydrogenase/reductase, conservec	5	0,82	0,07697065	CPIJ016922, CPIJ011026, CPIJ002522, CPIJ007736, CPIJ005655	546	42	14161	3,088	1	1	0,991014121
GOTERM_BP_DIRECT	GO:0042412~taurine biosynthetic process	2	0,33	0,077009	CPIJ010456, CPIJ020056	206	2	5220	25,340	0,99999751	1	1

GOTERM_BP_DIRECT	GO:0006839~mitochondrial transport	2	0,33	0,077009	CPIJ004238, CPIJ004795	206	2	5220	25,340	0,99999751	1	1
GOTERM_BP_DIRECT	GO:0009166~nucleotide catabolic process	3	0,49	0,07820416	CPIJ018314, CPIJ018313	206	12	5220	6,335	0,99999798	1	1
INTERPRO	IPR017871:ABC transporter, conserved site	6	0,98	0,08047091	CPIJ011962, CPIJ016097, CPIJ008283, CPIJ012364, CPIJ010068, CPIJ010067	546	60	14161	2,594	1	1	0,991014121
INTERPRO	IPR013818:Lipase, N-terminal	6	0,98	0,08972793	CPIJ004228, CPIJ004230, CPIJ001035, CPIJ004224, CPIJ004223	546	62	14161	2,510	1	1	0,991014121
INTERPRO	IPR000175:Sodium:neurotransmitter symporter	4	0,65	0,09128847	CPIJ012065, CPIJ003267, CPIJ012055, CPIJ012066	546	28	14161	3,705	1	1	0,991014121
INTERPRO	IPR000734:Lipase	6	0,98	0,09455459	CPIJ004228, CPIJ004230, CPIJ001035, CPIJ004224, CPIJ004223	546	63	14161	2,470	1	1	0,991014121
INTERPRO	IPR002423:Chaperonin Cpn60/TCP-1	4	0,65	0,09904984	CPIJ004080, CPIJ002450, CPIJ018377, CPIJ009388	546	29	14161	3,577	1	1	0,991014121
INTERPRO	IPR008758:Peptidase S28	3	0,49	0,0991685	CPIJ008878, CPIJ008877, CPIJ006124	546	14	14161	5,558	1	1	0,991014121
INTERPRO	IPR001604:DNA/RNA non-specific endonuclease	3	0,49	0,0991685	CPIJ011190, CPIJ003384, CPIJ011185	546	14	14161	5,558	1	1	0,991014121
INTERPRO	IPR013149:Alcohol dehydrogenase, C-terminal	3	0,49	0,0991685	CPIJ003802, CPIJ003801, CPIJ013381	546	14	14161	5,558	1	1	0,991014121
INTERPRO	IPR000873:AMP-dependent synthetase/ligase	5	0,82	0,09982404	CPIJ011600, CPIJ002867, CPIJ016639, CPIJ000426, CPIJ015670	546	46	14161	2,819	1	1	0,991014121

Table S5. Gene enrichment analysis with Gene Ontology (GO) terms and InterPro protein domain descriptions using differentially expressed genes from two Culex quinquefasciatus Bin-resistant strains (REC and REC-2), compared to a susceptible strain (S). Genes with log2 fold change equal to or greater than 1, and false discovery rates (FDR) corrected P-values lower than 0.05 were used.
(B) REC-2 X S up and down regulated genes.

Category	Term	Count	%	PValue	Genes	List Tot	Pop Hits	Pop Total	Fold Enrichment	Bonferroni	Benjamini	FDR
GOTERM_MF_DIRECT	GO:0005506~iron ion binding	33	6,86	2,16E-11	[illegible] CPIJ001757, CPIJ001758, CPIJ005956, CPIJ017014, CPIJ010546, CPIJ001759, CPIJ014220, CPIJ010544, CPIJ019673, CPIJ014221, CPIJ010541, CPIJ010231, CPIJ010175, CPIJ017306, CPIJ016855, CPIJ011127, CPIJ014219, CPIJ019704, CPIJ015428, CPIJ003082, CPIJ003361, CPIJ020056, CPIJ020199, CPIJ004411, CPIJ010538,	306	251	9432	4,052	3,76E-09	3,76E-09	3,67E-09
INTERPRO	IPR001128:Cytochrome P450	28	5,82	5,23E-11	CPIJ016846, CPIJ020229, CPIJ016848, CPIJ009478, CPIJ001757, CPIJ001758, CPIJ005956, CPIJ017014, CPIJ010546, CPIJ001759, CPIJ014220, CPIJ010544, CPIJ019673, CPIJ014221, CPIJ010541, CPIJ010231, CPIJ010175, CPIJ016855, CPIJ011127, CPIJ014219, CPIJ019704, CPIJ015428, CPIJ003082, CPIJ003361, CPIJ020199, CPIJ004411, CPIJ010538, CPIJ019702, CPIJ016852, CPIJ016853	416	204	14161	4,672	3,00E-08	3,01E-08	2,90E-08
GOTERM_MF_DIRECT	GO:0016705~oxidoreductase activity, acting on paired donors, witl	28	5,82	6,44E-10	CPIJ016846, CPIJ020229, CPIJ016848, CPIJ009478, CPIJ001757, CPIJ001758, CPIJ005956, CPIJ017014, CPIJ010546, CPIJ001759, CPIJ014220, CPIJ010544, CPIJ019673, CPIJ014221, CPIJ010541, CPIJ010231, CPIJ010175, CPIJ016855, CPIJ011127, CPIJ014219, CPIJ019704, CPIJ015428, CPIJ003082, CPIJ003361, CPIJ020199, CPIJ004411, CPIJ010538, CPIJ019702, CPIJ016852, CPIJ016853	306	209	9432	4,129	1,12E-07	5,22E-08	5,10E-08
GOTERM_MF_DIRECT	GO:0004497~monooxygenase activity	29	6,03	9,00E-10	CPIJ016846, CPIJ020229, CPIJ016848, CPIJ012907, CPIJ009478, CPIJ001757, CPIJ001758, CPIJ005956, CPIJ017014, CPIJ010546, CPIJ001759, CPIJ014220, CPIJ010544, CPIJ019673, CPIJ014221, CPIJ010541, CPIJ010231, CPIJ010175, CPIJ016855, CPIJ011127, CPIJ014219, CPIJ019704, CPIJ015428, CPIJ003082, CPIJ003361, CPIJ020199, CPIJ004411, CPIJ010538, CPIJ019702, CPIJ016852, CPIJ016853	306	227	9432	3,938	1,57E-07	5,22E-08	5,10E-08
INTERPRO	IPR017972:Cytochrome P450, conserved site	25	5,20	2,35E-10	CPIJ001758, CPIJ005956, CPIJ017014, CPIJ010546, CPIJ014220, CPIJ010544, CPIJ019673, CPIJ014221, CPIJ010541, CPIJ010231, CPIJ010175, CPIJ016855, CPIJ011127, CPIJ019704, CPIJ015428, CPIJ003082, CPIJ003361, CPIJ004411, CPIJ010538, CPIJ019702, CPIJ016852, CPIJ016853	416	173	14161	4,919	1,35E-07	6,75E-08	6,52E-08
GOTERM_MF_DIRECT	GO:0020037~heme binding	28	5,82	1,80E-08	[illegible] CPIJ001758, CPIJ005956, CPIJ017014, CPIJ010546, CPIJ001759, CPIJ014220, CPIJ010544, CPIJ019673, CPIJ014221, CPIJ010541, CPIJ010231, CPIJ010175, CPIJ016855, CPIJ011127, CPIJ014219, CPIJ019704, CPIJ015428, CPIJ003082, CPIJ003361, CPIJ020199, [illegible]	306	243	9432	3,552	3,13E-06	7,84E-07	7,66E-07
GOTERM_CC_DIRECT	GO:0005576~extracellular region	32	6,65	4,48E-08	[illegible] CPIJ004223, CPIJ004228, CPIJ005898, CPIJ014181, CPIJ002604, CPIJ012180, CPIJ001777, CPIJ016342, CPIJ018326, CPIJ017511,	206	392	7637	3,026	2,37E-06	2,37E-06	2,33E-06
GOTERM_BP_DIRECT	GO:0006749~glutathione metabolic process	10	2,08	5,15E-07	CPIJ018627, CPIJ002678, CPIJ002675, CPIJ018626, CPIJ002676, CPIJ020053, CPIJ002679, CPIJ014052, CPIJ018624, CPIJ000031	135	40	5220	9,667	5,98E-05	5,92E-05	5,92E-05
INTERPRO	IPR002401:Cytochrome P450, E-class, group I	20	4,16	3,69E-07	CPIJ010231, CPIJ016846, CPIJ010175, CPIJ011127, CPIJ003082, CPIJ015428, CPIJ020229, CPIJ016848, CPIJ003361, CPIJ004411, CPIJ009478, CPIJ001757, CPIJ001758, CPIJ010538, CPIJ005956, CPIJ010546, CPIJ014220, CPIJ010544, CPIJ019702, CPIJ014221, CPIJ010541	416	165	14161	4,126	2,12E-04	7,07E-05	6,82E-05
INTERPRO	IPR004045:Glutathione S-transferase, N-terminal	10	2,08	1,42E-06	CPIJ018627, CPIJ002678, CPIJ002675, CPIJ018626, CPIJ002676, CPIJ020053, CPIJ002679, CPIJ014052, CPIJ018624, CPIJ000031	416	39	14161	8,728	8,15E-04	2,04E-04	1,97E-04
INTERPRO	IPR010987:Glutathione S-transferase, C-terminal-like	10	2,08	2,23E-06	CPIJ018627, CPIJ002678, CPIJ002675, CPIJ018626, CPIJ002676, CPIJ020053, CPIJ002679, CPIJ014052, CPIJ018624, CPIJ000031	416	41	14161	8,303	0,001278134	2,56E-04	2,47E-04
INTERPRO	IPR004046:Glutathione S-transferase, C-terminal	9	1,87	4,73E-06	CPIJ018627, CPIJ002678, CPIJ002675, CPIJ018626, CPIJ002676, CPIJ020053, CPIJ002679, CPIJ014052, CPIJ018624	416	34	14161	9,011	0,002710165	4,53E-04	4,37E-04
GOTERM_CC_DIRECT	GO:0005615~extracellular space	11	2,29	4,00E-05	CPIJ000348, CPIJ001747, CPIJ006930, CPIJ006792, CPIJ015785, CPIJ016697, CPIJ012013, CPIJ016301	206	78	7637	5,228	0,002115452	0,0010588	0,001039
INTERPRO	IPR002402:Cytochrome P450, E-class, group II	6	1,25	4,97E-05	CPIJ014219, CPIJ019704, CPIJ001759, CPIJ016852, CPIJ020199, CPIJ016853	416	15	14161	13,616	0,028124428	0,0040824	0,00394
GOTERM_MF_DIRECT	GO:0008061~chitin binding	16	3,33	1,43E-04	[illegible] CPIJ004730, CPIJ019363, CPIJ005898, CPIJ002604, CPIJ014181, CPIJ019120, CPIJ014193, CPIJ016342, CPIJ017511, CPIJ016344,	306	155	9432	3,182	0,02465092	0,0049916	0,004877

INTERPRO	IPR002557:Chitin binding domain	14	2,91	1,55E-04	CPIJ015734, CPIJ011623, CPIJ008557, CPIJ008558, CPIJ004731, CPIJ00	416	134	14161	3,557	0,085138716	0,0111414	0,010754
GOTERM_MF_DIRECT	GO:0004556~alpha-amylase activity	5	1,04	4,32E-04	CPIJ005064, CPIJ005060, CPIJ005725, CPIJ008079, CPIJ001464	306	12	9432	12,843	0,07243363	0,0125291	0,012241
GOTERM_CC_DIRECT	GO:0043231~intracellular membrane-bounded organelle	5	1,04	7,12E-04	CPIJ019704, CPIJ014889, CPIJ016852, CPIJ016853	206	16	7637	11,585	0,037046414	0,0125789	0,012342
INTERPRO	IPR006046:Alpha amylase	5	1,04	2,98E-04	CPIJ005064, CPIJ005060, CPIJ005725, CPIJ008079, CPIJ001464	416	12	14161	14,184	0,157483272	0,0171635	0,016566
INTERPRO	IPR006048:Alpha-amylase, C-terminal all beta	5	1,04	2,98E-04	CPIJ005064, CPIJ005060, CPIJ005725, CPIJ008079, CPIJ001464	416	12	14161	14,184	0,157483272	0,0171635	0,016566
INTERPRO	IPR008930:Terpenoid cyclases/protein prenyltransferase a-a toroid	5	1,04	7,68E-04	CPIJ000348, CPIJ001747, CPIJ016574	416	15	14161	11,347	0,356446496	0,0401226	0,038727
INTERPRO	IPR019742:Alpha-2-macroglobulin, conserved site	4	0,83	0,001254	CPIJ000348, CPIJ001747	416	8	14161	17,020	0,513304658	0,0569847	0,055003
INTERPRO	IPR017853:Glycoside hydrolase, superfamily	11	2,29	0,001448	CPIJ019363, CPIJ005064, CPIJ002604, CPIJ019120, CPIJ008904, CPIJ005060, CPIJ005725, CPIJ013173, CPIJ008079, CPIJ001464, CPIJ017511	416	110	14161	3,404	0,564627237	0,0569847	0,055003
INTERPRO	IPR015897:CHK kinase-like	8	1,66	0,001462	CPIJ012697, CPIJ012699, CPIJ017118, CPIJ012700, CPIJ010308, CPIJ012702, CPIJ010307, CPIJ010310	416	58	14161	4,695	0,568228758	0,0569847	0,055003
INTERPRO	IPR013780:Glycosyl hydrolase, family 13, all-beta	7	1,46	0,001487	CPIJ005064, CPIJ008904, CPIJ005060, CPIJ005725, CPIJ013173, CPIJ008079, CPIJ001464	416	43	14161	5,542	0,574256476	0,0569847	0,055003
INTERPRO	IPR002890:Alpha-2-macroglobulin, N-terminal	4	0,83	0,00184	CPIJ000348, CPIJ001747	416	9	14161	15,129	0,652531455	0,0661218	0,063822
INTERPRO	IPR004119:Protein of unknown function DUF227	8	1,66	0,002374	CPIJ012697, CPIJ012699, CPIJ017118, CPIJ012700, CPIJ010308, CPIJ012702, CPIJ010307, CPIJ010310	416	63	14161	4,323	0,744492273	0,077824	0,075117
INTERPRO	IPR009048:Alpha-macroglobulin, receptor-binding	4	0,83	0,002572	CPIJ000348, CPIJ001747	416	10	14161	13,616	0,771903367	0,077824	0,075117
INTERPRO	IPR001599:Alpha-2-macroglobulin	4	0,83	0,002572	CPIJ000348, CPIJ001747	416	10	14161	13,616	0,771903367	0,077824	0,075117
INTERPRO	IPR002213:UDP-glucuronosyl/UDP-glucosyltransferase	6	1,25	0,002913	[illegible] CPIJ020264	416	34	14161	6,007	0,812553686	0,0801805	0,077392
INTERPRO	IPR003172:MD-2-related lipid-recognition (ML) domain	5	1,04	0,002928	CPIJ002743, CPIJ002744, CPIJ012180, CPIJ002740, CPIJ018326	416	21	14161	8,105	0,814245091	0,0801805	0,077392
INTERPRO	IPR006047:Glycosyl hydrolase, family 13, catalytic domain	6	1,25	0,003318	CPIJ005064, CPIJ005060, CPIJ005725, CPIJ013173, CPIJ008079, CPIJ001464	416	35	14161	5,836	0,851549901	0,0828846	0,080002
INTERPRO	IPR011626:A-macroglobulin complement component	4	0,83	0,00346	CPIJ000348, CPIJ001747	416	11	14161	12,378	0,863197054	0,0828846	0,080002
INTERPRO	IPR011625:Alpha-2-macroglobulin, N-terminal 2	4	0,83	0,00346	CPIJ000348, CPIJ001747	416	11	14161	12,378	0,863197054	0,0828846	0,080002
INTERPRO	IPR005829:Sugar transporter, conserved site	6	1,25	0,003762	CPIJ012675, CPIJ001812, CPIJ017472, CPIJ000191, CPIJ017473	416	36	14161	5,673	0,885056161	0,0865201	0,083511
GOTERM_MF_DIRECT	GO:0004866~endopeptidase inhibitor activity	4	0,83	0,004554	CPIJ000348, CPIJ001747	306	11	9432	11,209	0,548082208	0,1132064	0,110604
INTERPRO	IPR001314:Peptidase S1A, chymotrypsin-type	19	3,95	0,007404	CPIJ006075, CPIJ007385, CPIJ015106, CPIJ017789, CPIJ007384, CPIJ019428, CPIJ017738, CPIJ011616, CPIJ009625, CPIJ002156, CPIJ002138, CPIJ002139, CPIJ002659, CPIJ017793, CPIJ001979, CPIJ017792, CPIJ011382, CPIJ016224, CPIJ011298	416	324	14161	1,996	0,985954226	0,1637318	0,158037
INTERPRO	IPR001254:Peptidase S1	22	4,57	0,00788	[illegible] CPIJ019428, CPIJ017738, CPIJ008523, CPIJ011616, CPIJ009625, CPIJ002156, CPIJ002138, CPIJ002139, CPIJ002659, CPIJ006967, CPIJ017793, CPIJ001979, CPIJ011381, CPIJ017792, CPIJ011382, CPIJ016224, CPIJ011298	416	402	14161	1,863	0,989334854	0,1678052	0,161969
INTERPRO	IPR013818:Lipase, N-terminal	7	1,46	0,009419	CPIJ004228, CPIJ011478, CPIJ004230, CPIJ004225, CPIJ004224, CPIJ004223	416	62	14161	3,843	0,995625479	0,1934198	0,186692
INTERPRO	IPR009003:Trypsin-like cysteine/serine peptidase domain	22	4,57	0,009757	CPIJ006075, CPIJ007385, CPIJ015106, CPIJ017789, CPIJ007384, CPIJ019428, CPIJ017738, CPIJ008523, CPIJ011616, CPIJ009625, CPIJ002156, CPIJ002138, CPIJ002139, CPIJ002659, CPIJ006967, CPIJ017793, CPIJ001979, CPIJ011381, CPIJ017792, CPIJ011382, CPIJ016224, CPIJ011298	416	411	14161	1,822	0,996403693	0,1934519	0,186723
INTERPRO	IPR000734:Lipase	7	1,46	0,01016	CPIJ004228, CPIJ011478, CPIJ004230, CPIJ004225, CPIJ004224, CPIJ004223	416	63	14161	3,782	0,997153864	0,1947375	0,187964
GOTERM_BP_DIRECT	GO:0006629~lipid metabolic process	8	1,66	0,00364	CPIJ004228, CPIJ002103, CPIJ011478, CPIJ004230, CPIJ004225, CPIJ004224, CPIJ004223	135	78	5220	3,966	0,3449553	0,2093205	0,20932
INTERPRO	IPR004911:Gamma interferon inducible lysosomal thiol reductase (	3	0,62	0,011885	CPIJ008702, CPIJ007525, CPIJ007524	416	6	14161	17,020	0,998954	0,2201117	0,212456
INTERPRO	IPR001734:Sodium/solute symporter	4	0,83	0,012515	CPIJ002534, CPIJ002361, CPIJ000151, CPIJ001859	416	17	14161	8,010	0,999274797	0,2201117	0,212456
INTERPRO	IPR005828:General substrate transporter	7	1,46	0,012632	CPIJ012675, CPIJ001773, CPIJ001812, CPIJ017472, CPIJ000191, CPIJ017473	416	66	14161	3,610	0,999322595	0,2201117	0,212456
INTERPRO	IPR019826:Carboxylesterase type B, active site	6	1,25	0,013968	CPIJ007828, CPIJ018231, CPIJ018233, CPIJ013917, CPIJ013918	416	49	14161	4,168	0,999688583	0,2304508	0,222435
INTERPRO	IPR020846:Major facilitator superfamily domain	9	1,87	0,014027	CPIJ012675, CPIJ001773, CPIJ001812, CPIJ017472, CPIJ000191, CPIJ017473, CPIJ004817, CPIJ007652	416	108	14161	2,837	0,999699107	0,2304508	0,222435
INTERPRO	IPR011051:RmlC-like cupin domain	3	0,62	0,016318	CPIJ000377, CPIJ010456, CPIJ020056	416	7	14161	14,589	0,999920841	0,2606416	0,251576
INTERPRO	IPR002181:Fibrinogen, alpha/beta/gamma chain, C-terminal globu	8	1,66	0,017354	CPIJ006120, CPIJ012831, CPIJ007727, CPIJ013538, CPIJ012040, CPIJ014772, CPIJ012829, CPIJ018159	416	91	14161	2,993	0,999956745	0,2625869	0,253453
INTERPRO	IPR014716:Fibrinogen, alpha/beta/gamma chain, C-terminal globu	8	1,66	0,017354	CPIJ006120, CPIJ012831, CPIJ007727, CPIJ013538, CPIJ012040, CPIJ014772, CPIJ012829, CPIJ018159	416	91	14161	2,993	0,999956745	0,2625869	0,253453

GOTERM_CC_DIRECT	GO:0016021~integral component of membrane	106	22,04	0,01999	CPIJ010068, CPIJ017306, CPIJ011602, CPIJ005322, CPIJ000151, CPIJ000670, CPIJ003267, CPIJ003305, CPIJ002336, CPIJ018192, CPIJ000039, CPIJ005050, CPIJ014327, CPIJ011457, CPIJ005697, CPIJ009656, CPIJ005698, CPIJ005214, CPIJ009812, CPIJ005699, CPIJ005611, CPIJ005692, CPIJ006267, CPIJ004646, CPIJ001773, CPIJ004369, CPIJ001812, CPIJ010762, CPIJ016344, CPIJ010495, CPIJ012675, CPIJ011346, CPIJ014219, CPIJ004492, CPIJ003163, CPIJ004373, CPIJ006677, CPIJ005468, CPIJ008456, CPIJ002077, CPIJ010538, CPIJ008859, CPIJ019347, CPIJ010259, CPIJ004817, CPIJ017721, CPIJ007010, CPIJ002361, CPIJ004141, CPIJ007652, CPIJ000225, CPIJ008109, CPIJ019996, CPIJ010546, CPIJ010544, CPIJ012681, CPIJ014221, CPIJ010541, CPIJ008353, CPIJ000191, CPIJ002493, CPIJ012979, CPIJ000192, CPIJ009449, CPIJ020199, CPIJ006617, CPIJ002534, CPIJ016093, CPIJ002659, CPIJ017065, CPIJ016097, CPIJ016094, CPIJ019125, CPIJ012055, CPIJ018314, CPIJ005492, CPIJ011417, CPIJ006347, CPIJ009976, CPIJ006220, CPIJ000881, CPIJ009698, CPIJ001335, CPIJ017472, CPIJ017592, CPIJ001859, CPIJ017473, CPIJ012065, CPIJ012066, CPIJ010175, CPIJ011026, CPIJ012511, CPIJ003082, CPIJ003007, CPIJ001747,	206	3355	7637	1,171	0,657062529	0,2648686	0,259871
GOTERM_MF_DIRECT	GO:0016671~oxidoreductase activity, acting on a sulfur group of d	3	0,62	0,014343	CPIJ008702, CPIJ007525, CPIJ007524	306	6	9432	15,412	0,919035595	0,2744354	0,268127
GOTERM_MF_DIRECT	GO:0004180~carboxypeptidase activity	5	1,04	0,015138	CPIJ010495, CPIJ009106, CPIJ008878, CPIJ008877, CPIJ008873	306	30	9432	5,137	0,929646513	0,2744354	0,268127
GOTERM_MF_DIRECT	GO:0004806~triglyceride lipase activity	7	1,46	0,015772	CPIJ004228, CPIJ011478, CPIJ004230, CPIJ004225, CPIJ004224, CPIJ004223	306	63	9432	3,425	0,9371011	0,2744354	0,268127
GOTERM_MF_DIRECT	GO:0015020~glucuronosyltransferase activity	4	0,83	0,019056	CPIJ004369, CPIJ004373, CPIJ010762, CPIJ020264	306	18	9432	6,850	0,964835679	0,3014295	0,2945
GOTERM_BP_DIRECT	GO:0005975~carbohydrate metabolic process	11	2,29	0,008366	CPIJ005060, CPIJ005725, CPIJ013173, CPIJ008079, CPIJ001464, CPIJ017511	135	162	5220	2,626	0,622613181	0,3206791	0,320679
INTERPRO	IPR014710:RmlC-like jelly roll fold	5	1,04	0,022395	CPIJ000377, CPIJ010259, CPIJ010456, CPIJ020056	416	37	14161	4,600	0,999997741	0,3228108	0,311583
INTERPRO	IPR003663:Sugar/inositol transporter	4	0,83	0,022456	CPIJ012675, CPIJ001812, CPIJ000191	416	21	14161	6,484	0,999997822	0,3228108	0,311583
GOTERM_MF_DIRECT	GO:0016491~oxidoreductase activity	13	2,70	0,028011	CPIJ000840, CPIJ005656, CPIJ007736, CPIJ008859, CPIJ008447, CPIJ005655, CPIJ013919	306	199	9432	2,014	0,992870565	0,4061632	0,396826
INTERPRO	IPR008334:5'-Nucleotidase, C-terminal	3	0,62	0,032996	CPIJ018314, CPIJ018313	416	10	14161	10,212	0,999999996	0,4627543	0,446659
INTERPRO	IPR020904:Short-chain dehydrogenase/reductase, conserved site	5	1,04	0,03389	CPIJ016922, CPIJ011026, CPIJ002522, CPIJ007736, CPIJ005655	416	42	14161	4,052	0,999999997	0,4639767	0,447838
GOTERM_MF_DIRECT	GO:0004252~serine-type endopeptidase activity	22	4,57	0,034865	[illegible] CPIJ019428, CPIJ017738, CPIJ008523, CPIJ011616, CPIJ009625, CPIJ002156, CPIJ002138, CPIJ002139, CPIJ002659, CPIJ006967, CPIJ017793, CPIJ001979, CPIJ011381, CPIJ017792, CPIJ011382, CPIJ016224, CPIJ011298	306	425	9432	1,596	0,997918712	0,4666531	0,455925
INTERPRO	IPR011583:Chitinase II	4	0,83	0,035647	CPIJ019363, CPIJ002604, CPIJ019120, CPIJ017511	416	25	14161	5,447	0,999999999	0,4766695	0,46009
INTERPRO	IPR002018:Carboxylesterase, type B	7	1,46	0,036629	CPIJ007828, CPIJ018231, CPIJ018233, CPIJ013917, CPIJ013918, CPIJ008422	416	84	14161	2,837	1	0,4786737	0,462024
INTERPRO	IPR001223:Glycoside hydrolase, family 18, catalytic domain	4	0,83	0,039446	CPIJ019363, CPIJ002604, CPIJ019120, CPIJ017511	416	26	14161	5,237	1	0,4945018	0,477302
INTERPRO	IPR006179:5'-Nucleotidase/apyrase	3	0,62	0,03956	CPIJ018314, CPIJ018313	416	11	14161	9,284	1	0,4945018	0,477302
GOTERM_MF_DIRECT	GO:0016740~transferase activity	9	1,87	0,045779	CPIJ002523, CPIJ018627, CPIJ002678, CPIJ002675, CPIJ018626, CPIJ002676, CPIJ020053, CPIJ002679, CPIJ018624	306	123	9432	2,255	0,999712319	0,5484221	0,535815
GOTERM_MF_DIRECT	GO:0051119~sugar transmembrane transporter activity	3	0,62	0,047278	CPIJ012675, CPIJ001812	306	11	9432	8,406	0,999781155	0,5484221	0,535815
GOTERM_CC_DIRECT	GO:0005861~troponin complex	2	0,42	0,052969	CPIJ014296, CPIJ009743	206	2	7637	37,073	0,9441137	0,5614701	0,550876
INTERPRO	IPR000175:Sodium:neurotransmitter symporter	4	0,83	0,047632	CPIJ012065, CPIJ003267, CPIJ012055, CPIJ012066	416	28	14161	4,863	1	0,5827294	0,562461
INTERPRO	IPR019172:Osteopetrosis-associated transmembrane protein 1 prec	2	0,42	0,057755	CPIJ016093, CPIJ016094	416	2	14161	34,041	1	0,6511577	0,628509
INTERPRO	IPR003950:Potassium channel, voltage-dependent, ELK	2	0,42	0,057755	CPIJ010259	416	2	14161	34,041	1	0,6511577	0,628509
INTERPRO	IPR010300:Cysteine dioxygenase type I	2	0,42	0,057755	CPIJ010456, CPIJ020056	416	2	14161	34,041	1	0,6511577	0,628509
INTERPRO	IPR001978:Troponin	2	0,42	0,057755	CPIJ014296, CPIJ009743	416	2	14161	34,041	1	0,6511577	0,628509
GOTERM_MF_DIRECT	GO:0017172~cysteine dioxygenase activity	2	0,42	0,063631	CPIJ010456, CPIJ020056	306	2	9432	30,824	0,999989241	0,6572212	0,642113
GOTERM_MF_DIRECT	GO:0052689~carboxylic ester hydrolase activity	5	1,04	0,06455	CPIJ007828, CPIJ018233, CPIJ013917, CPIJ013918	306	47	9432	3,279	0,999990931	0,6572212	0,642113
GOTERM_MF_DIRECT	GO:0016787~hydrolase activity	8	1,66	0,067988	CPIJ017592, CPIJ001128, CPIJ011190, CPIJ005468, CPIJ011185, CPIJ011373, CPIJ018314	306	111	9432	2,222	0,999995221	0,6572212	0,642113
INTERPRO	IPR008758:Peptidase S28	3	0,62	0,0618	CPIJ008878, CPIJ008877, CPIJ008873	416	14	14161	7,294	1	0,6704739	0,647153
INTERPRO	IPR003010:Carbon-nitrogen hydrolase	3	0,62	0,0618	CPIJ007583, CPIJ017593, CPIJ017592	416	14	14161	7,294	1	0,6704739	0,647153
GOTERM_CC_DIRECT	GO:0005777~peroxisome	4	0,83	0,07607	CPIJ013919, CPIJ003059, CPIJ000670	206	37	7637	4,008	0,984904025	0,671955	0,659277
GOTERM_MF_DIRECT	GO:0016811~hydrolase activity, acting on carbon-nitrogen (but no	2	0,42	0,093917	CPIJ007583, CPIJ017593	306	3	9432	20,549	0,999999965	0,7963488	0,778042
GOTERM_MF_DIRECT	GO:0022857~transmembrane transporter activity	10	2,08	0,094628	CPIJ002534, CPIJ001773, CPIJ017472, CPIJ000191, CPIJ002361, CPIJ000151, CPIJ001859, CPIJ017473, CPIJ004817, CPIJ007652	306	168	9432	1,835	0,999999969	0,7963488	0,778042
GOTERM_MF_DIRECT	GO:0071949~FAD binding	4	0,83	0,096111	CPIJ013919, CPIJ003059, CPIJ000670	306	34	9432	3,626	0,999999977	0,7963488	0,778042
INTERPRO	IPR006091:Acyl-CoA oxidase/dehydrogenase, central domain	3	0,62	0,078453	CPIJ011633, CPIJ016453, CPIJ003059	416	16	14161	6,383	1	0,8201867	0,791658

INTERPRO	IPR009100:Acyl-CoA dehydrogenase/oxidase	3	0,62	0,078453	CPIJ011633, CPIJ016453, CPIJ003059	416	16	14161	6,383	1	0,8201867	0,791658
INTERPRO	IPR012101:Biotinidase, eukaryotic	2	0,42	0,085372	CPIJ007583, CPIJ017593	416	3	14161	22,694	1	0,8612092	0,831254
INTERPRO	IPR008547:Protein of unknown function DUF829, TMEM53	2	0,42	0,085372	CPIJ019996, CPIJ006220	416	3	14161	22,694	1	0,8612092	0,831254
INTERPRO	IPR002110:Ankyrin repeat	8	1,66	0,096202	CPIJ010052, CPIJ008490, CPIJ006306, CPIJ009924, CPIJ013539, CPIJ017156, CPIJ018694, CPIJ018744	416	133	14161	2,048	1	0,9229965	0,890892
INTERPRO	IPR001173:Glycosyl transferase, family 2	3	0,62	0,096313	CPIJ005697, CPIJ005698, CPIJ005699	416	18	14161	5,673	1	0,9229965	0,890892
INTERPRO	IPR001353:Proteasome, subunit alpha/beta	3	0,62	0,096313	CPIJ000897, CPIJ017386, CPIJ007179	416	18	14161	5,673	1	0,9229965	0,890892
GOTERM_BP_DIRECT	GO:0009166~nucleotide catabolic process	3	0,62	0,03648	CPIJ018314, CPIJ018313	135	12	5220	9,667	0,986577043	1	1
GOTERM_BP_DIRECT	GO:0042412~taurine biosynthetic process	2	0,42	0,050687	CPIJ010456, CPIJ020056	135	2	5220	38,667	0,997603908	1	1
GOTERM_BP_DIRECT	GO:0006807~nitrogen compound metabolic process	3	0,62	0,084177	CPIJ007583, CPIJ017593, CPIJ017592	135	19	5220	6,105	0,999962833	1	1

Table S6. Dataset of the RT-qPCR assays for the RNAseq validation in pool of twenty fourth instar larvae of *Culex quinquefasciatus* from two Bin-resistant strains (REC, REC-2) compared to a susceptible strain (S). Samples in triplicates were used. Ct. Cycle threshold. Rq. Relative quantification. Log2FC. Log2 fold change.

**Gene/sample**	**Ct**	**ΔCt**	**Rq**	**Log2FC**	**Average**
CPIJ013173 Cqm1					
S1	22.566	9.767	1	0	**-0.63**
S2	21.938	9.731	1.025	0.0356239	
S3	22.796	10.438	0.628	-0.6711635	
REC 1	33.633	20.04	0.001	-9.9657843	**-9.63**
REC 2	32.89	20.236	0.001	-9.9657843	
REC 3	31.05	18.514	0.002	-8.9657843	
REC2 1	28.484	15.849	0.015	-6.0588937	**-5.4**
REC2 2	27.428	14.801	0.031	-5.011588	
REC2 3	27.477	14.946	0.028	-5.1584294	
CPIJ017593 Pantetheinase precursor					
S1	22.342	9.543	1	0	-0.6
S2	22.202	9.996	0.731	-0.4520567	
S3	23.263	10.904	0.389	-1.3621579	
REC 1	25.819	12.226	0.156	-2.6803821	-2.74
REC 2	24.898	12.244	0.154	-2.6989977	
REC 3	24.938	12.402	0.138	-2.8572598	
REC2 1	27.312	14.676	0.028	-5.1584294	-4.09
REC2 2	26.287	13.66	0.058	-4.1078033	
REC2 3	25.088	12.557	0.124	-3.011588	
CPIJ012066 Sodium/chloride dependent amino acid transporter					
S1	22.238	8.673	1	0	-2.32
S3	20.493	8.194	1.394	-4.6438562	
REC 1	35.043	19.548	0.001	-9.9657843	-9.96
REC 2	33.98	19.418	0.001	-9.9657843	
REC 3	34.097	19.832	0.000		
REC2 1	30.128	17.659	0.002	-8.9657843	-9.29
REC2 2	31.296	18.133	0.001	-9.9657843	
REC2 3	30.221	18.029	0.002	-8.9657843	
CPIJ010801 Carboxypeptidase B precursor					
S1	25.643	13.298	1	0	**-0.04**
S2	26.091	13.528	0.853	-0.2293824	
S3	25.636	13.214	1.06	0.0840643	
REC 1	21.884	9.11	18.226	4.1879261	**5.16**
REC 2	20.676	7.604	51.758	5.69371	
REC 3	20.288	7.691	48.729	5.6067087	
REC2 1	21.989	9.358	15.353	3.9404487	**3.97**
REC2 2	22.038	9.279	16.214	4.0191681	
REC2 3	21.915	9.343	15.512	3.9553128	
CPIJ002522 Farnesol dehydrogenase					
S1	30.979	18.634	1	0	**-0.04**

S2	30.344	17.781	1.806	0.8527979	
S3	30.391	17.97	1.585	0.6644828	
REC 2	23.688	10.617	259.056	8.0171202	**7.99**
REC 3	23.26	10.664	250.773	7.9702382	
REC2 2	25.303	12.544	68.135	6.0903242	**5.55**
REC2 3	26.193	13.62	32.305	5.0136856	

Table S7. Statistics of the RT-qPCR assays for the relative quantification of the transcripts of *Culex quinquefasciatus* fourth instar larvae from two Bin-resistant strains (REC, REC-2) compared to a susceptible one (S). Transcripts are from five differentially expressed genes (DEGs) revealed by RNAseq and transcripts assessed in individual larvae.

Gene	Analysis	*P*[1]	t-test
**DEGs[2] (RNAseq)**			
	S x REC	< 0.0001	t(4)= 23.27
Cqm1	S x REC-2	0.0002	t(4)= 12.99
	REC x REC-2	0,0008	t(4)= 9.038
	S x REC	0.0061	t(4)= 5.293
Pan	S x REC-2	0.0091	t(4)= 4.726
	REC x REC-2	0,0964	t(4)= 2.165
	S x REC	0.0006	t(2)= 42.59
NaCl	S x REC-2	0.0003	t(3)= 20.47
	REC x REC-2	0.2191	t(3)= 1.549
	S x REC	0.0005	t(4)= 10.49
CbB	S x REC-2	< 0.0001	t(4)= 41.56
	REC x REC-2	0,0714	t(4)= 2.438
	S x REC	0.0002	t(3)= 22.39
Far	S x REC-2	0.0024	t(3)= 9.667
	REC x REC-2	0.0454	t(2)= 4.531
**Pant (individual larvae)**			
	S x REC	< 0.0001	t(8)= 69.35
Pan	S x REC-2	< 0.0001	t(7.2)= 73.40
	REC x REC-2	0.06	t(1.9)= 65.68

[1] $p \leq 0.05$ is significantly different, for t-test. [2] Cqm1-*Culex quinquefasciatus* maltase 1 (CPIJ013173); Pan-pantetheinase (CPIJ017593); NaCl-Sodium/chloride dependent amino acid transporter putative (CPIJ012066); CbB-Carboxypeptidase B precursor (CPIJ010801); Far-Farnesol dehydrogenase (CPIJ002522).

Table S8. Genes of *Culex quinquefasciatus* pantetheinases found in Vectorbase (www.vectorbase.org accessed on 20 September 2023), their location and identity towards CPIJ017593.

Identity	Description	Genomic sequence	Location Start-End (strand)	Genomic sequence	Transcript lenght (bp)	AA[1] (No.)	ID[2] (%)	LFC[3] RNA-seq	
								REC	REC-2
CPIJ017593	Pantetheinase precursor	DS232811	111820-114238 (+)	DS232811	1551	516	Reference	-8.8	-8.2
CPIJ007582	Vanin-like protein 1[4]	DS231994	288993-290747 (+)	DS231994	1581	526	96	ND[5]	-0.67
CPIJ007581	Vanin-like protein 1	DS232811	99896-108188 (-)	DS232811	1566	521	64	1.12	ND
CPIJ017592	Vanin-like protein 1	DS231994	275664-280417 (-)	DS231994	1668	555	62	-7.9	-8.3
CPIJ007583	Pantetheinase precursor	DS231994	322817-324748 (+)	DS231994	1563	520	55	ND	-1.96
CPIJ007584	Uncharacterized protein	DS231994	356529-358371 (+)	DS231994	1485	494	31	-0.51	-0.08

[1] Amino acids; [2] Identity considering the global alignment; [3] Log2 fold change; [4] Vanin-like protein 1 precursor putative. [5] Non detected.

Table S9. Dataset of the RT-qPCR assays for the relative quantification of the pantheteinase transcripts (CPIJ017593) in individual *Culex quinquefasciatus* fourth instar larvae from two Bin-resistant strains (REC, REC-2) compared to a susceptible strain (S). Ct. Cycle threshold. Rq. Relative quantification. A. Average. SD. Standard deviation. R. Reference sample.

S										REC									REC 2								
N	Ct 18S	Wells	Ct Pant	Wells	ΔCt	ΔΔCt	Rq	Log2 FC	Ct 18S	Wells	Ct Pant	Wells	ΔCt	ΔΔCt	Rq	Log2 FC	Ct 18S	Wells	Ct Pant	Wells	ΔCt	ΔΔCt	Rq	Log2 FC
R	9.47	A1A2	28.03	E1E2	18.56	0.00	1.00	0.00	9.47	A1A2	28.03	E1E2	18.56	0.00	1.00	0.00	9.47	A1A2	28.03	E1E2	18.56	0.00	1.00	0.00
1	13.39	A3A4	30.14	E3E4	16.74	-1.82	3.52	1.82	12.53	C3C4	31.63	G3G4	19.10	0.54	0.69	-0.54	12.53	A3A4	35.87	E3E4	23.33	4.77	0.04	-4.77
2	12.28	A5A6	27.67	E5E6	15.40	-3.16	8.94	3.16	11.15	C5C6	29.85	G5G6	18.70	0.14	0.91	-0.14	11.15	A5A6	33.01	E5E6	21.87	3.31	0.10	-3.31
3	10.69	A7A8	27.74	E7E8	17.05	-1.52	2.86	1.52	13.70	C7C8	33.64	G7G8	19.94	1.38	0.39	-1.38	11.55	A7A8	34.98	E7E8	23.43	4.87	0.03	-4.87
4	12.15	A9A10	32.18	E9E10	20.03	1.47	0.36	-1.47	9.85	C9C10	38.05	G9G10	28.19	9.63	0.001	-9.63	11.25	A9A10	32.30	E9E10	21.04	2.48	0.18	-2.48
5	9.67	A11A12	29.58	E11E12	19.91	1.35	0.39	-1.35	10.07	C11C12	36.63	G11G12	26.56	8.00	0.004	-8.00	11.86	A11A12	33.16	E11E12	21.30	2.74	0.15	-2.74
6	11.92	B3B4	31.61	F3F4	19.69	1.13	0.46	-1.13	10.35	D1D2	33.62	H1H2	23.27	4.71	0.04	-4.71	12.59	B3B4	31.90	F3F4	19.31	0.75	0.60	-0.75
7	9.64	B5B6	28.38	F5F6	18.74	0.18	0.88	-0.18	12.64	D3D4	34.10	H3.H4	21.47	2.91	0.13	-2.91	13.66	B5B6	33.85	F5F6	20.20	1.64	0.32	-1.64
8	13.04	B7B8	32.25	F7F8	19.21	0.65	0.64	-0.65	13.06	D5D6	36.69	H5H6	23.63	5.07	0.03	-5.07	12.70	B7B8	35.70	F7F8	23.01	4.45	0.05	-4.45
9	13.72	B9B10	32.87	F9F10	19.15	0.59	0.66	-0.59	13.41	D7D8	37.72	H7H8	24.32	5.76	0.02	-5.76	11.88	B9B10	32.96	F9F10	21.07	2.51	0.18	-2.51
10	12.61	B11B12	31.84	F11F12	19.23	0.67	0.63	-0.67	10.35	D9D10	34.67	H9H10	24.31	5.75	0.02	-5.75	12.01	B11B12	35.41	F11F12	23.40	4.84	0.04	-4.84
11	11.02	A3A4	28.51	E3E4	17.49	-1.07	2.10	1.07	10.77	C1C2	36.03	G1G2	25.25	6.69	0.01	-6.69	12.46	C1C2	34.47	G1G2	22.01	3.45	0.09	-3.45
12	13.70	A5A6	31.64	E5E6	17.94	-0.62	1.54	0.62	11.72	C3C4	35.41	G3G4	23.69	5.13	0.03	-5.13	12.98	C3C4	35.94	G3G4	22.95	4.39	0.05	-4.39
13	13.94	A7A8	30.69	E7E8	16.75	-1.81	3.52	1.81	11.99	C5C6	31.82	G5G6	19.84	1.34	0.40	-1.34	11.25	C7C8	32.77	G7G8	21.52	2.96	0.13	-2.96
14	8.32	A9A10	28.50	E9E10	20.18	1.62	0.33	-1.62	12.75	C7C8	33.44	G7G8	20.70	2.20	0.22	-2.20	13.32	C9C10	35.37	G9G10	22.05	3.49	0.09	-3.49
15	10.43	A11A12	29.71	E11E12	19.28	0.72	0.61	-0.72	12.84	C9C10	34.20	G9G10	21.36	2.86	0.14	-2.86	13.71	C11C12	33.11	G11G12	19.39	0.83	0.56	-0.83
16	11.95	B1B2	28.74	F1F2	16.79	-1.77	3.42	1.77	13.12	C11C12	35.52	G11G12	22.40	3.90	0.07	-3.90	11.32	D1D2	30.70	H1H2	19.38	0.82	0.57	-0.82
17	11.68	B3B4	27.91	F3F4	16.23	-2.33	5.01	2.33	10.91	D3D4	32.94	H3.H4	22.02	3.52	0.09	-3.52	13.01	D3D4	32.11	H3H4	19.10	0.54	0.69	-0.54
18	11.22	B5B6	30.17	F5F6	18.94	0.38	0.77	-0.38	10.76	D5D6	32.89	H5H6	22.13	3.63	0.08	-3.63	11.98	D5D6	33.07	H5H6	21.09	2.53	0.17	-2.53
19	11.17	B7B8	25.97	F7F8	14.80	-3.76	13.57	3.76	14.15	D7D8	38.09	H7H8	23.94	5.44	0.02	-5.44	11.92	D7D8	32.71	H7H8	20.80	2.24	0.21	-2.24
20	10.64	B9B10	30.20	F9F10	19.56	1.00	0.50	-1.00	10.99	B3B4	30.24	F3F4	19.25	0.75	0.60	-0.75	12.98	D9D10	35.57	H9H10	22.59	4.03	0.06	-4.03
21	14.49	B11B12	30.08	F11F12	15.58	-2.98	7.87	2.98	11.54	B1B2	31.08	F1F2	19.54	1.04	0.49	-1.04	11.04	D11D12	31.32	H11H12	20.27	1.71	0.30	-1.71
22	12.90	A1A2	30.49	E1E2	17.59	-0.97	1.96	0.97	12.72	B3B4	31.84	F3F4	19.12	0.62	0.65	-0.62	11.83	A3A4	31.42	E3E4	19.59	1.03	0.49	-1.03
23	11.50	A3A4	30.01	E3E4	18.51	-0.05	1.04	0.05	10.34	B5B6	32.11	F5F6	21.77	3.27	0.10	-3.27	10.45	A5A6	29.78	E5E6	19.33	0.77	0.59	-0.77
24	9.32	A1A2	28.06	E1E2	18.75	0.18	0.88	-0.18	9.88	B7B8	30.89	F7F8	21.02	2.52	0.17	-2.52	10.62	A7A8	31.07	E7E8	20.45	1.89	0.27	-1.89
25	8.39	A5A6	24.09	E5E6	15.70	-2.86	7.28	2.86	12.83	B3B4	31.85	F3F4	19.02	0.52	0.70	-0.52	10.58	A11A12	29.77	E11E12	19.20	0.64	0.64	-0.64
26	8.01	A7A8	28.18	E7E8	20.17	1.61	0.33	-1.61	10.31	B5B6	32.15	F5F6	21.84	3.34	0.10	-3.34	10.71	B1B2	29.71	F1F2	19.00	0.44	0.74	-0.44
27	12.37	A1A2	28.24	E1E2	15.86	-2.70	6.49	2.70	9.96	B7B8	31.27	F7F8	21.30	2.80	0.14	-2.80	11.43	B7B8	30.98	F78	19.56	1.00	0.50	-1.00

28	8.66	A3A4	25.95	E3E4	17.29	-1.27	2.41	1.27	12.64	B1B2	31.13	F1F2	18.49	-0.01	1.01	0.01	11.45	B11B12	30.33	F11F12	18.88	0.32	0.80	-0.32
29	8.31	A7A8	25.92	E7E8	17.61	-0.95	1.93	0.95	12.80	B3B4	34.52	F3F4	21.71	3.21	0.11	-3.21	10.80	C1C2	29.40	G1G2	18.61	0.05	0.97	-0.05
30	13.52	A1A2	27.80	E1E2	14.28	-4.28	19.41	4.28	12.25	B5B6	33.06	F5F6	20.81	2.31	0.20	-2.31	11.55	C3C4	30.67	G3G4	19.12	0.56	0.68	-0.56
31	10.90	A3A4	27.77	E3E4	16.87	-1.69	3.22	1.69	11.49	B7B8	32.24	F7F8	20.76	2.26	0.21	-2.26	10.98	C5C6	29.91	G5G6	18.93	0.37	0.77	-0.37
32	11.01	A5A6	30.48	E5E6	19.46	0.90	0.53	-0.90	13.23	G1G2	31.57	C1C2	18.34	-0.16	1.12	0.16	11.11	C7C8	30.13	G7G8	19.02	0.46	0.73	-0.46
33	9.20	A7A8	28.65	E7E8	19.45	0.89	0.54	-0.89	12.26	G5G6	32.51	C5C6	20.26	1.76	0.30	-1.76	11.32	C9C10	30.04	G9G10	18.73	0.17	0.89	-0.17
34	9.21	A9A10	28.52	E9E10	19.31	0.75	0.59	-0.75	11.37	G7G8	31.46	C7C8	20.09	1.59	0.33	-1.59								
35	10.24	A1A2	27.44	E1E2	17.21	-1.35	2.55	1.35	10.94	H3H4	32.67	D3D4	21.73	3.23	0.11	-3.23								
36	9.93	A3A4	24.81	E3E4	14.87	-3.69	12.87	3.69	11.27	H5H6	30.80	D5D6	19.54	1.04	0.49	-1.04								
37	8.90	A5A6	23.91	E5E6	15.01	-3.55	11.73	3.55	12.08	H7H8	30.70	D7D8	18.62	0.12	0.92	-0.12								
38	9.95	A7A8	24.42	E7E8	14.46	-4.10	17.11	4.10	12.36	H9H10	32.65	D9D10	20.29	1.79	0.29	-1.79								
39	11.04	E1E2	31.52	A1A2	20.48	1.92	0.26	-1.92																
40	10.47	E3E4	30.79	A3A4	20.32	1.76	0.29	-1.76																
41	12.08	E5E6	30.88	A4A6	18.80	0.24	0.85	-0.24																
42	10.93	E7E8	27.36	A7A8	16.44	-2.12	4.36	2.12																
43	10.68	F1F2	29.53	B1B2	18.85	0.29	0.82	-0.29																
44	11.87	F5F6	28.90	B5B6	17.02	-1.54	2.90	1.54																
A	11.07		28.86		17.80		3.61	**0.76**	11.77		33.20		21.43		0.30	**-2.91**	11.82		32.41		20.59		0.38	**-2.03**
SD	1.68		2.22		1.79		4.68	**1.79**	1.18		2.20		2.28		0.31	**2.27**	0.90		2.08		1.55		0.29	**1.55**

Figure S1. Principal Component Analysis (PCA) of the expression profile generated by RNA-seq comparing the *Culex quinquefasciatus* larvae from the Bin-resistant strains (REC and REC-2) and the susceptible strain (S). **(A)** Comparison between REC (replicates A11. A12. A13 in red) and S (replicates S1, S2, S3 in blue). **(B)** Comparison between REC-2 (replicates A21. A22. A23 in red) and S (replicates S1, S2, S3 in blue).

A

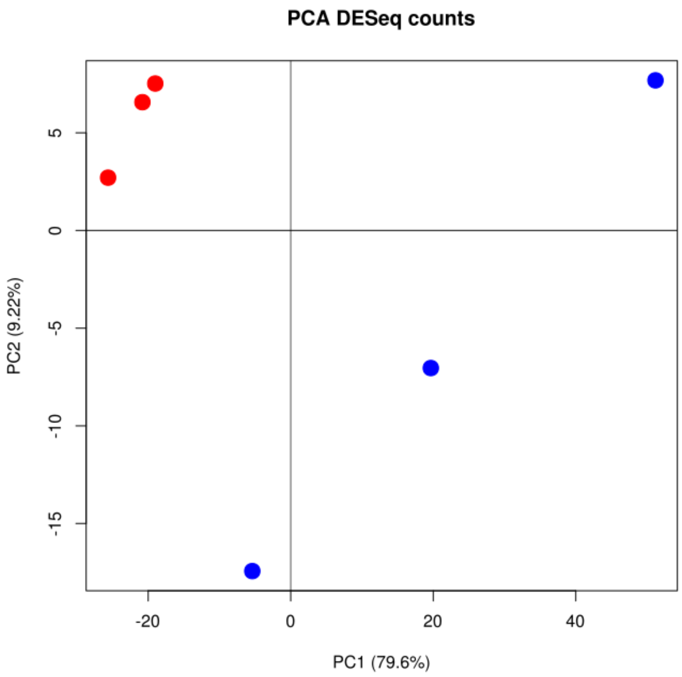

B

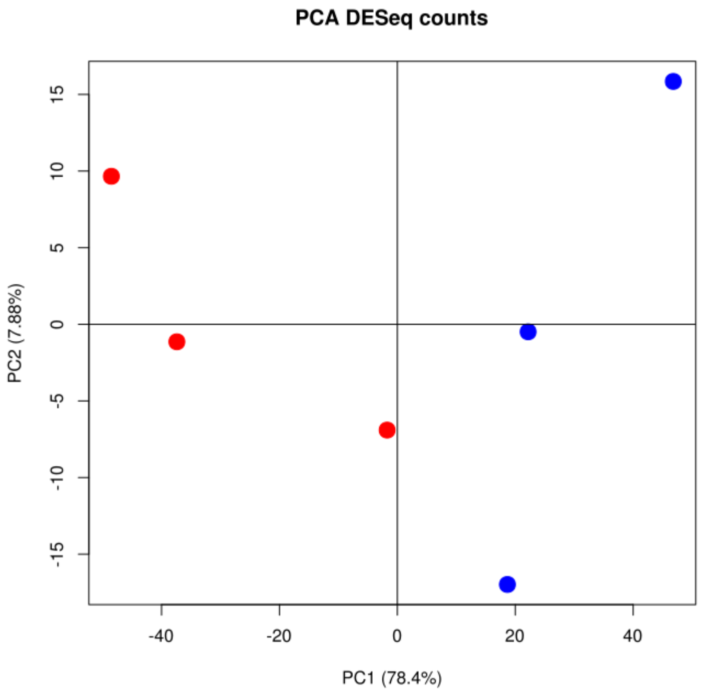

Figure S2. Gene enrichment analysis with Gene Ontology (GO) terms and InterPro protein domain descriptions using genes, with Log2 fold change ≥ 1 and false discovery rates corrected *p*-values ≤ 0.05, found with the two colonies of Bin-resistant *Culex quinquefasciatus* larvae (REC and REC-2) compared to a susceptible colony (S), obtained by RNA-seq. The fold-enrichment of genes in each term is represented by bars and the number of genes is on the right. The complete GO dataset is available in Table S5.

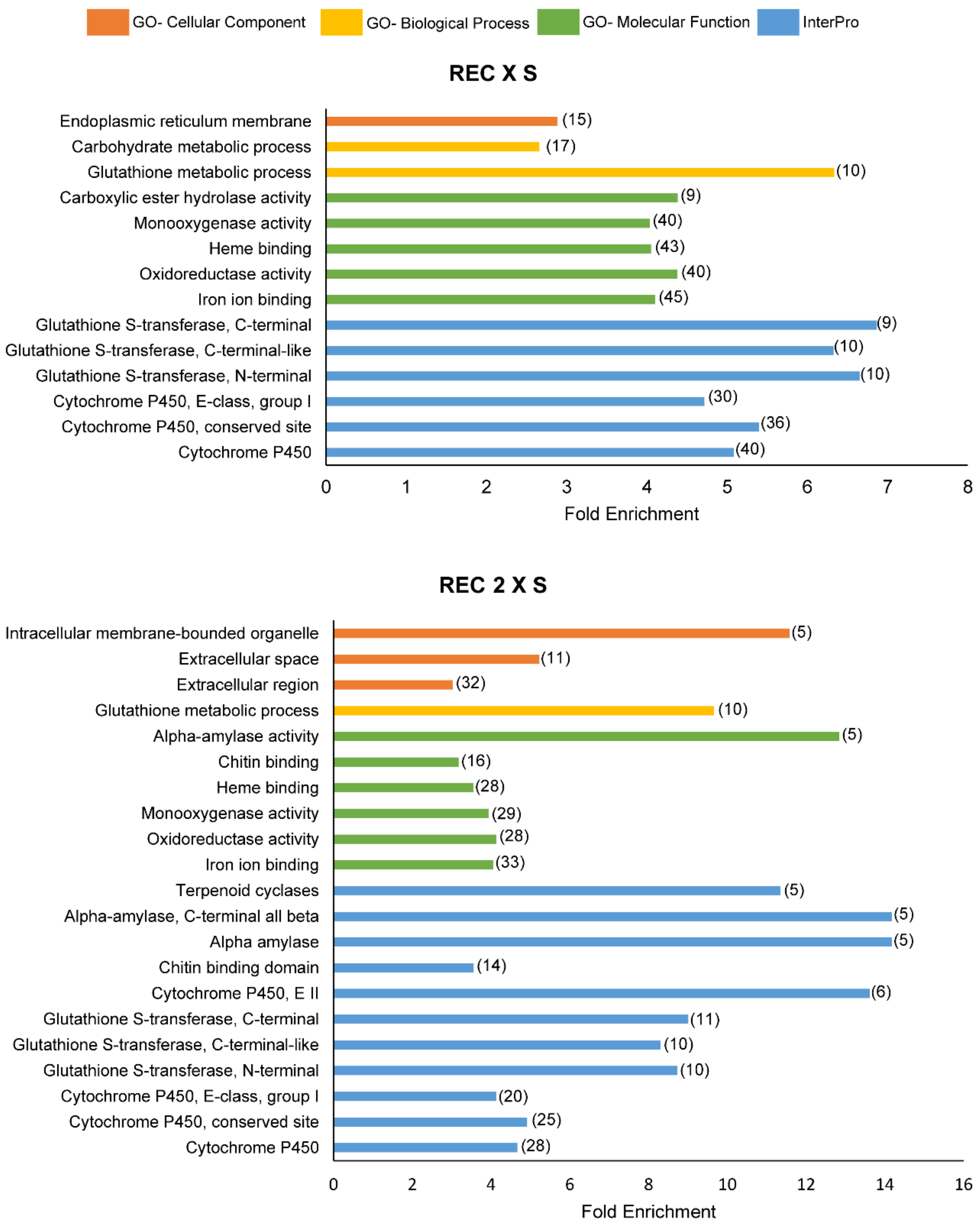

Figure S3. Immunodetection of recombinant *Culex quinquefasciatus* pantetheinase (CPIJ017593) produced in *Escherichia coli* (1) and *Sf*9 cells (2). Proteins were immunoblotted using polyclonal antibodies raised against the *E. coli* recombinant pantetheinase. Molecular weight markers (kDa) are on the left. Arrows show ~ 70 kDa and ~ 45 kDa proteins.

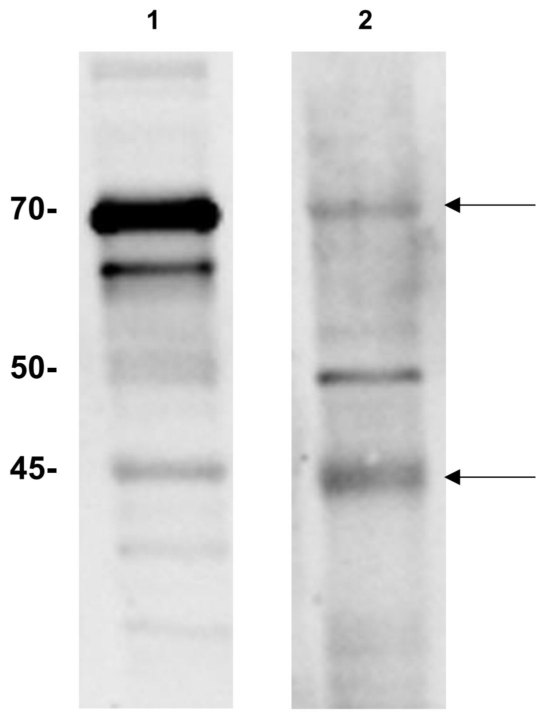

Figure 6 A and B- Coomassie and Western blot

Coomassie (A)

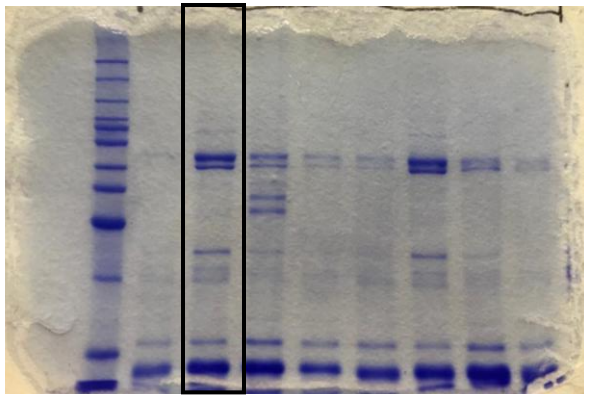

Anti-His (A)

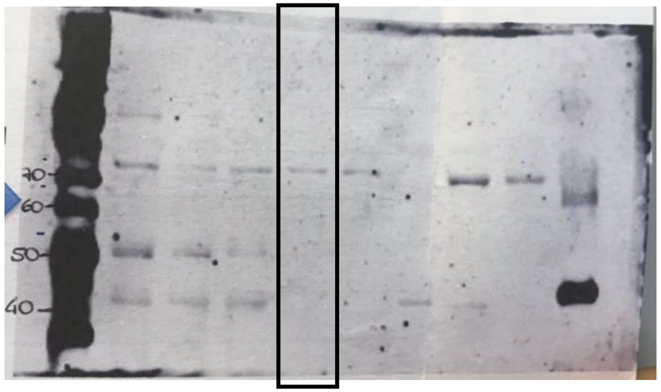

Anti-Pan (A)

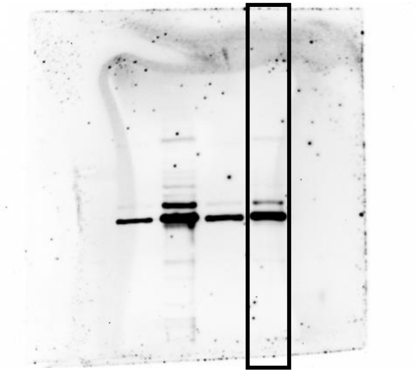

Coomassie (B)

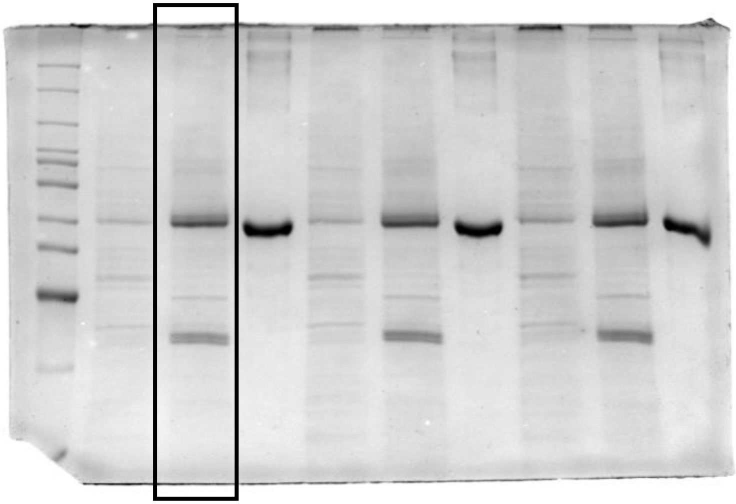

Anti-His (B)

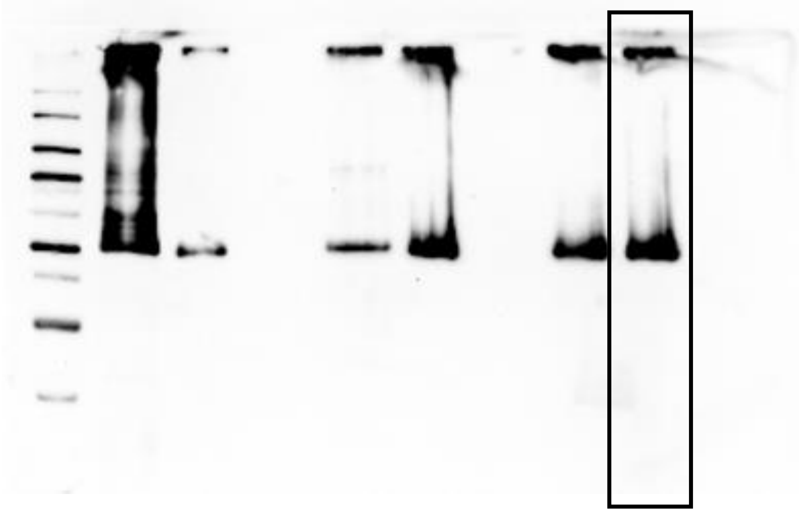

Anti-Pan (B)

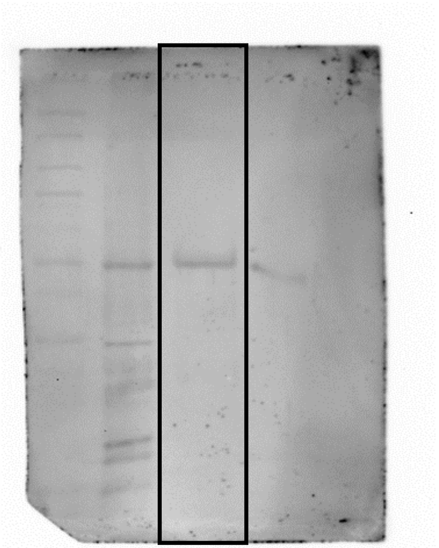

Figure 6 C- Coomassie (glycosylation)

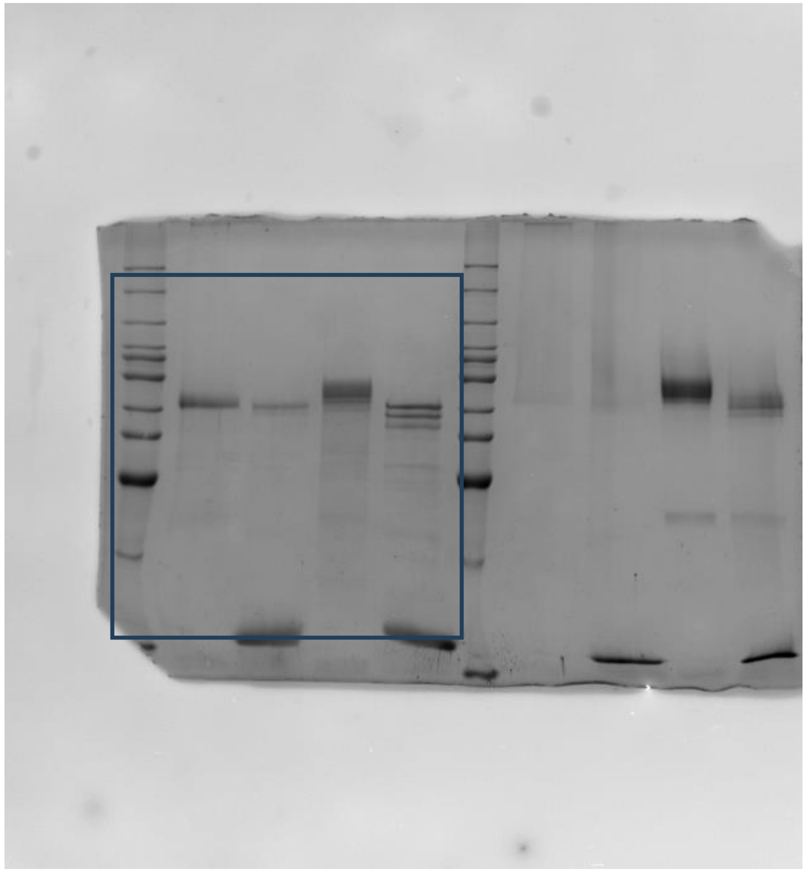

Figure 7 B- Western blot (BBMF)

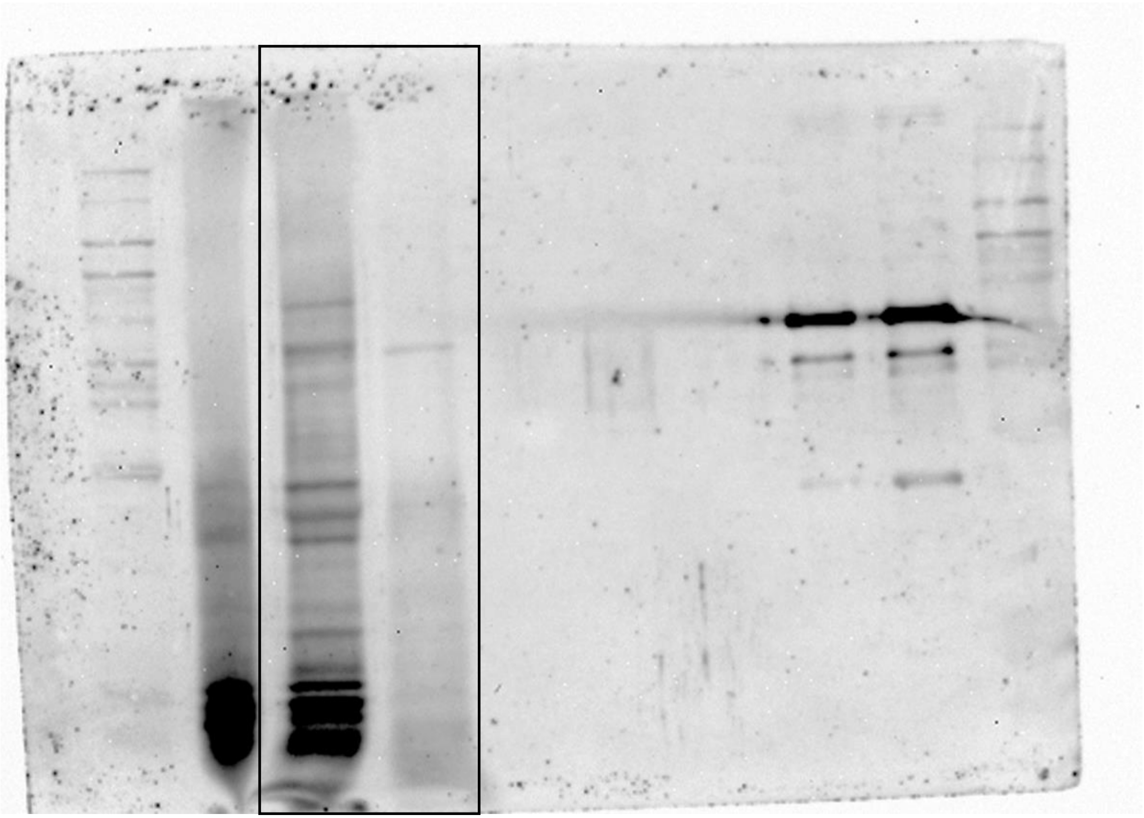

Figure S3- Western blot (45 kDa protein in SF9 e *E.Coli*)

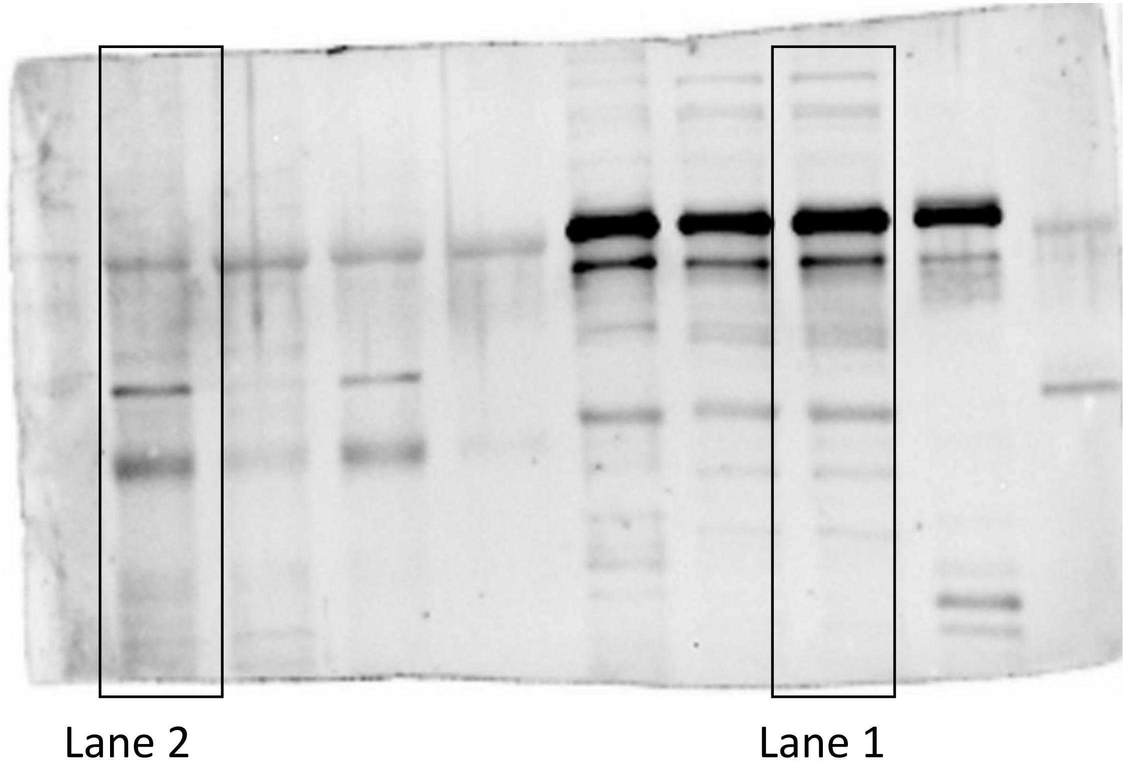

Supplement: Supplementary file 1 [file biomolecules-14-00033-s001.zip › biomolecules-2665646-supplementary.pdf]
